# Integrating Network Pharmacology and Experimental Validation to Elucidate the Mechanism of Yiqi Yangyin Decoction in Suppressing Non-Small-Cell Lung Cancer

**DOI:** 10.1155/2023/4967544

**Published:** 2023-02-20

**Authors:** Pengfei Jiao, Yingrui Wang, Gaofei Ren, Dan Chu, Yameng Li, Tianqing Sang

**Affiliations:** ^1^Department of General Diseases, The First Affiliated Hospital of Zhengzhou University, Zhengzhou 450000, China; ^2^Department of Oncology, The First Affiliated Hospital of Henan University of Chinese Medicine, Zhengzhou 450000, China; ^3^Department of Endocrinology, The First Affiliated Hospital of Zhengzhou University, Zhengzhou 450000, China

## Abstract

Yiqi Yangyin Decoction (YYD) is a classic traditional Chinese medicine (TCM) formulation to treat lung cancer in clinic. Nevertheless, the active ingredients, key targets, and molecular mechanisms for YYD are still poorly understood. This study is focused on elucidating the pharmacological mechanism of YYD in non-small-cell lung cancer (NSCLC) by using a combined network pharmacology approach and biological experiment validation. Online bioinformatics tools showed that 40 bioactive compounds and 229 putative targets of YYD were associated with anti-NSCLC activity. Protein-Protein Interaction (PPI) network demonstrated AKT1, SRC, JUN, TP53, and EGFR as the top five key targets for YYD against NSCLC. Through enrichment analysis, YYD was found to affect cell proliferation and apoptosis in NSCLC possibly by PI3K-AKT signaling. Molecular docking confirmed a strong binding between the main compounds (quercetin or luteolin) and EGFR. As demonstrated by CCK-8, EdU, and colony formation assays, we found a significant inhibition of YYD on cell proliferation. Moreover, YYD treatment induced cell cycle arrest by affecting p53, p21, and cyclin D1 expression. YYD administration enhanced apoptosis by changing the expression of cleaved caspase-3, Bax, and Bcl-2. Mechanistically, YYD resulted in a significant inactivation of EGFR-PI3K-AKT signaling. Furthermore, EGFR activator significantly reversed YYD-mediated proliferation inhibition and apoptosis. YYD also showed an inhibitory effect on tumor growth in mice. Together, YYD might target the EGFR-PI3K-AKT pathway to repress NSCLC progression.

## 1. Introduction

Lung cancer is the second most commonly diagnosed cancer and is also the most prevalent cause of cancer deaths according to GLOBOCAN in 2020 [1]. Non-small-cell lung cancer (NSCLC) and small cell lung cancer (SCLC) are the major histological subtypes for lung cancer, representing 85% and 15% of cases, respectively [2]. Due to the inconspicuous symptoms at the early stage, most lung cancer patients progress to a late-stage disease accompanied with a worse prognosis. As reported, the 5-year relative survival rate for lung cancer patients is 19% and is higher for NSCLC (23%) than SCLC (6%) [3]. What is worse, a survival chance of less than 10% was found in patients with IVA-IVB disease [4]. The severe adverse reaction and drug resistance make chemotherapy no longer the optimal choice for patients with advanced and metastatic NSCLC [5]. The emergence of targeted therapy and immune checkpoint inhibitors improves long-term survival outcomes in advanced NSCLC, but only a small fraction of the population benefit from these agents [6]. Therefore, there is an urgent need to search for effective medications with lower toxicity and less drug resistance for NSCLC.

Traditional Chinese medicine (TCM) is a common alternative for malignant tumors due to the characteristics of the abundant resources, low toxicity, and multitarget [7]. TCM hinders NSCLC progression by increasing the body's anticancer immunity or suppressing tumor cell growth, proliferation, invasion, and migration [8]. Yiqi Yangyin Decoction (YYD), an empirical TCM prescription for replenishing Qi and nourishing Yin, has been applied for lung cancer treatment in clinical practice. YYD is mainly used to regulate the imbalance by “strengthening the body” and “eliminating evil.” YYD is composed of seven herbs: Panax Ginseng C. A. Mey (Ren Shen), Polygonati Rhizoma (Huang Jing), Ophiopogon Japonicus (Mai Dong), Lilii Bulbus (Bai He), Adenophorae Ae Radix (Nan Sha Shen), Trichosanthis Radix (Tian Hua Fen), and Agrimonia Eupatoria (Xian He Cao) at the ratio of 4 : 5 : 5 : 10 : 5:5 : 10. The majority of herbs in this formula have been reported to exert anticancer activities by different pharmacological mechanisms [9–14]. YYD increased the chemosensitivity in advanced ovarian cancer [15] and leukemia stem cells [16]. Clinically, YYD was found to enhance the apatinib sensitivity in mid-advanced NSCLC [17]. YYD also promoted cisplatin-induced tumor eradication and suppressed interleukin-7 reduction in NSCLC [18]. However, the biological functions and pharmacological mechanisms of YYD in inhibiting NSCLC have not been comprehensively investigated with appropriate methods.

TCM is a multitarget, multichannel, and multilink system with complex chemical compositions. Thus, conventional pharmacological approaches are unable to systematically identify the action mechanism of TCM. Network pharmacology is an effective approach to predict the potential mechanism of drug action in disease therapy on the basis of a network perspective and systems biology [19]. Network pharmacology is employed to screen active ingredients, predict herb targets, understand biological foundations, and elucidate molecular pathways in TCM research [20]. In this study, we intend to explore the anticancer activities and molecular mechanisms of YYD in NSCLC with network pharmacology and experimental validation.

## 2. Materials and Methods

### 2.1. Identification of Chemical Ingredients in YYD

Traditional Chinese Medicine Systems Pharmacology Database (TCMSP) was used to search the active compounds in YYD. Oral bioavailability (OB) ≥ 30%, drug-likeness (DL) ≥ 0.18, and half-life (HL) ≥ 4 h were regarded as screening threshold for the qualified herbal compounds.

### 2.2. Prediction of Candidate Targets for YYD Compounds

The compound-related targets were obtained from the TCMSP, PharmMapper (norm fit ≥ 0.7), TargetNet (probability ≥ 0.5), and SwissTargetPrediction (probability ≥ 0.5). We used the UniProt database to standardize the official gene symbols corresponding to the proteins. After integrating the duplicate items, we gained the candidate targets of YYD compounds.

### 2.3. Collecting the Targets of YYD against NSCLC

The protein-coding genes associated with NSCLC were retrieved from GeneCards (relevance score ≥ 30, category: protein coding), DisGeNET, and DrugBank. The overlapping genes from compound- and NSCLC-related targets were defined as the potential therapeutic targets for YYD to combat NSCLC.

### 2.4. Protein-Protein Interaction (PPI) Network Establishment

The STRING database and Cytoscape 3.7.2 software were used to establish and analyze the PPI network. The CytoNCA plugin was used to identify the core targets in this network based on key topological parameters. The MCODE plugin was applied to assess the clustered modules in this network.

### 2.5. Enrichment Analysis

The GO function and KEGG pathway enrichment for the hub genes were conducted by using DAVID. GO items and relevant pathways with *p* < 0.05 indicated a superior prediction for the biological processes and mechanisms of YYD in NSCLC treatment.

### 2.6. Network Construction and Analysis

The herb-compound-target, compound-target-disease, and compound-target-pathway networks were established by using the Cytoscape 3.7.2 software.

### 2.7. Molecular Docking

The 3D molecule structures of compounds (luteolin and quercetin) for molecular docking were downloaded from PubChem database and saved as a PDB file. The RCSB PDB database was applied to acquire the protein crystal structures of AKT1 (PDB ID: 6NPZ), EGFR (PDB ID: 2RGP), and TP53 (PDB ID 7DHZ). The protein structures were imported to PyMOL 2.5 software to get rid of water molecules, salt ions, and other undesirable molecules. Then, docking boxes were defined to enclose the entire protein structure. Subsequently, we used ADFRsuite 1.0 for the conversion of small molecules and receptor proteins to PDBQT format. At last, AutoDock Vina 1.1.2 software was run for molecular docking. PyMOL 2.5 software was used for visualizing the best scoring conformation.

### 2.8. YYD Preparation

YYD consists of Panax Ginseng C. A. Mey (PG, Ren Shen, 12 g), Polygonati Rhizoma (PR, Huang Jing, 15 g), Ophiopogon Japonicus (OJ, Mai Dong, 15 g), Lilii Bulbus (LB, Bai He, 30 g), Adenophorae Ae Radix (AAR, Nan Sha Shen, 15 g), Trichosanthis Radix (TR, Tian Hua Fen, 15 g), and Agrimonia Eupatoria (AE, Xian He Cao, 30 g). To prepare YYD, all crude herbs were soaked in 10 volumes of distilled water for 30 min and then were decocted for 1 h for 2 times. The herbal extract was centrifuged at 1000 rpm for 30 min to collect the supernatant. The extraction procedure was repeated two times. Then, the supernatants were mixed and evaporated to obtain a powder. Finally, the dry powder was dissolved in DMSO to 125 mg/mL, filtered with a 0.22 *μ*m filter, and reserved at −20°C. The mass spectra of YYD were performed and established for quality control.

### 2.9. Cell Culture and Treatment

Human NSCLC cells (A549 and H1975) were cultured in RPMI-1640 medium containing 10% FBS in a humidified 5% CO_2_ atmosphere at 37°C. EGFR activation was performed by using specific agonist treatment (NSC228155, 100 *μ*M, Selleckchem).

### 2.10. Cell Counting Kit-8 (CCK-8) Assay

CCK-8 (Beyotime, Shanghai, China) was used to measure cell viability. NSCLC cells were seeded in 96-well plates to allow for adhesion overnight. Then, cells were treated with YYD (0, 7.8125, 15.625, 31.25, 62.5, 125, 250, 500, and 1000 *μ*g/ml) for 24 h or 48 h. After adding 10 *μ*l of CCK-8 solution to each well, we put the plates to the incubator for 2 h of incubation at 37°C. The absorbance at 450 nm was examined.

### 2.11. EdU Assay

NSCLC cells were seeded into 96-well plates and maintained overnight for cell attachment. Then, cell culture was performed in a medium containing YYD (0, 125, and 250 *μ*g/ml) for 48 h. Cell-Light™ EdU Apollo 567 In Vitro Kit (RiboBio, Guangzhou, China) was applied to detect the ability of cell proliferation. Briefly, EdU solution was added to each well and maintained at 37°C for 2 h. After discarding the medium, we subjected the cells to 4% paraformaldehyde for fixation, 0.5% Triton X-100 for permeabilization, and 1× Apollo staining solution for staining. Thereafter, cells were counterstained with 1× Hoechst 33342 nuclear staining dye for 30 min. The EdU-positive cells were analyzed under a fluorescence microscope.

### 2.12. Colony Formation Assay

NSCLC cells were inoculated in 6-well plates and treated with indicated doses of YYD (0, 125, and 250 *μ*g/ml) for 48 h. Next, the YYD-containing medium was removed. Cells were kept in a fresh complete medium for an incubation period of 9 days, with refreshing of medium every 3 days. After being fixed with 4% paraformaldehyde and stained with 0.1% crystal violet, cells were subjected to an optical microscope for counting colonies.

### 2.13. Flow Cytometry Assay of Cell Cycle and Apoptosis

NSCLC cells were treated with specified dosages of YYD (0, 125, and 250 *μ*g/ml) for 48 h. For cell cycle analysis, cells were collected and subjected to 70% ethanol overnight at −20°C. The next day, cells were washed with PBS, incubated with RNase A, and stained with propidium iodide (PI). For apoptosis analysis, the harvested cells were stained with 5 *μ*l annexin V-FITC and 5 *μ*l PI for 15 min in a dark place. A FACScan flow cytometer (BD Biosciences, San Jose, CA, USA) was applied to measure cell cycle distribution and apoptosis.

### 2.14. Western Blot Assay

YYD-treated cells were lysed using RIPA lysis buffer (CWBIO, Beijing, China). The protein samples (40 *μ*g) were loaded onto a 10% SDS-PAGE for separation and then transferred into a PVDF membrane. The membranes were incubated with the primary antibodies overnight at 4°C with constant stirring. Subsequently, the appropriate secondary antibody was added for another 2 h of incubation at room temperature. At last, an enhanced chemiluminescence reagent was used to visualize the immunoreactive bands, and ImageJ software was used to calculate the protein intensity. Primary antibodies against p53, p21, cyclin D1, Bax, Bcl-2, cleaved caspase-3, EGFR, p-EGFR, PI3K, p-PI3K, AKT, p-AKT, and GAPDH were purchased from Cell Signaling Technology (Danvers, MA, USA).

### 2.15. Nude Mouse Xenograft Assay

Male BALB/c nude mice (aged 4–5 weeks, weighing 20 ± 2 g) were maintained in a specific pathogen-free room with appropriate temperature and humidity and enough food and water. After one week of acclimatization, 1 × 10^7^ A549 cells were subcutaneously injected into the right armpits of nude mice. Six days later, mice were randomly allocated into 4 groups: control group, YYD (25 mg/kg) group, YYD (50 mg/kg) group, and afatinib (10 mg/kg) group. Mice in the YYD group were intragastrically administered with 25 or 50 mg/kg YYD daily. Mice in the afatinib group were intragastrically administered with 10 mg/kg afatinib daily. Mice in the control group were intragastrically given equal volumes of PBS. Tumor volumes were calculated according to the formula *V* (mm^3^) = 0.5 × length × width^2^. After medication for 21 days, mice were euthanized and subcutaneous tumors were removed.

### 2.16. Immunohistochemical Staining

The xenograft tumors were fixed with 4% formaldehyde, paraffin-embedded, and cut into 4 *μ*m slices. Then, immunohistochemical staining was performed according to a previously published reference [21]. The slices were incubated with primary antibodies against p-EGFR, Ki-67, cyclin D1, and Bax (Cell Signaling Technology) at 4°C overnight. The next day, the slices were further incubated with appropriate secondary antibody for 1 h at room temperature. After being stained with 3,3-diaminobenzidine, the slices were observed with a microscope.

### 2.17. Statistical Assay

Data are analyzed using GraphPad Prism 7 software and described as mean ± SD. Student's *t*-test and one-way ANOVA were used for analyzing statistical significance. A *p* value of less than 0.05 indicates a statistically significant result.

## 3. Results

### 3.1. Identification of Bioactive Compounds and Targets in YYD

**Figure 1:**
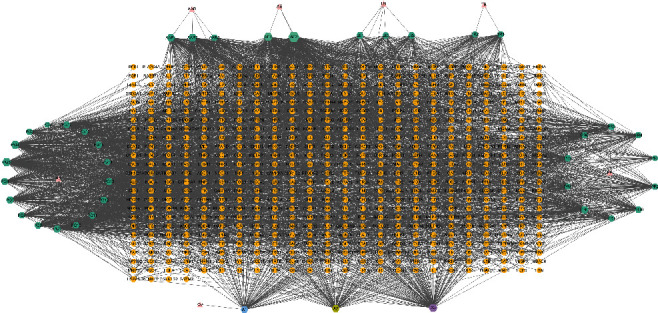
Herb-compound-target network for YYD. Triangles represent 7 herbs, hexagons represent 40 bioactive compounds, and circles represent 580 targets.

According to the criteria of OB ≥ 30%, DL ≥ 0.18, and HL ≥ 4 in TCMSP, a total of 40 bioactive compounds of YYD were obtained, 20 of which belong to Panax Ginseng C. A. Mey (PG), 3 to Agrimonia Eupatoria (AE), 2 to Trichosanthis Radix (TR), 11 to Polygonati Rhizoma (PR), 5 to Lilii Bulbus (LB), 4 to Adenophorae Ae Radix (AAR), and 1 to Ophiopogon Japonicus (OJ) (Table 1). TCMSP, PharmMapper, TargetNet, and SwissTargetPrediction were used to predict the targets of 40 bioactive compounds. After high-possibility screening and overlap elimination, we finally achieved 580 targets (Supplementary Table 1). A “herb-compound-target” network was established, as presented in Figure 1. This network consists of 627 nodes (7 herbs, 40 bioactive compounds, and 580 targets) and 3996 edges. On the basis of degree value, we acquired the top five ingredients including AE2 (quercetin), PG5 (arachidonate), AE1 (luteolin), PG4 (dianthramine), and A3 (kaempferol).

### 3.2. Target Identification of YYD on NSCLC

**Figure 2:**
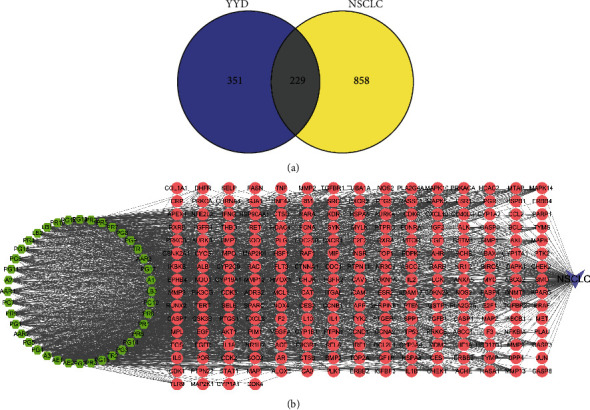
Identifying the potential targets for YYD against NSCLC. (a) Venn diagram showing the common targets for YYD and NSCLC. (b) The compound-target-disease network of YYD against NSCLC. The green hexagons represent bioactive compounds, the red circles represent targets, and the purple V shapes represent NSCLC.

By searching GeneCards, DisGeNET, and DrugBank, we obtained 1087 NSCLC-associated targets (Supplementary Table 2). By comparing the targets of compounds with those NSCLC-related targets, a total of 229 overlapping genes were identified (Figure 2(a) and Supplementary Table 3). Then, a “compound-target-disease” network was constructed by the Cytoscape software. As shown in Figure 2(b), this network contained 270 nodes (40 compound nodes, 229 target nodes, and 1 disease node) and 2143 edges. Based on the degree value, the top ten bioactive compounds were AE2 (quercetin), AE1 (luteolin), A3 (kaempferol), PG4 (dianthramine), PG5 (arachidonate), AAR2 (ethyl oleate (NF)), PG1 (chrysanthemaxanthin), PR9 (baicalein), PG7 (ginsenoside rh2), and AAR1 (mandenol). These compounds were considered the main bioactive components for YYD to treat NSCLC.

### 3.3. PPI Network Analysis

**Figure 3:**
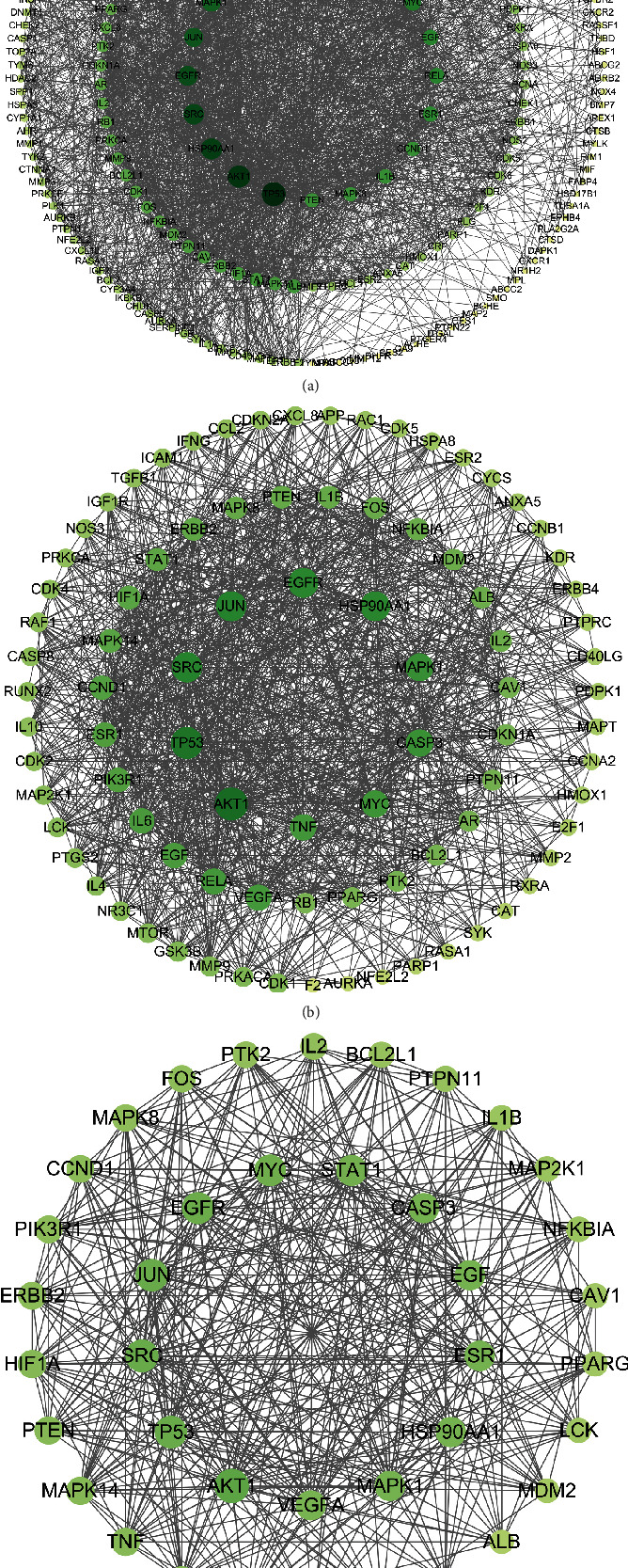
Identification of hub genes for YYD to treat NSCLC. (a) The PPI network of overlapping genes of YYD targets and NSCLC-associated targets. (b) PPI network of important targets derived from (a). (c) PPI network of hub genes.

The 229 common genes were entered into the STRING database and then were visualized by Cytoscape software. After hiding the disconnected nodes, we obtained a PPI network harboring 228 nodes and 2307 edges (Figure 3(a)). On the basis of the degree centrality (DC), betweenness centrality (BC), and closeness centrality (CC), two rounds of screening for hub genes were performed. Targets with BC, CC, and DC greater than the median values were selected to build the core network for YYD against NSCLC. With DC ≥ 16, BC ≥ 84.05, and CC ≥ 0.44, the first screening result was a network including 88 nodes and 1133 edges (Figure 3(b)). According to DC ≥ 22, BC ≥ 28.8, and CC ≥ 0.57, the second screening ended up with 39 nodes and 421 edges (Figure 3(c)). The nodes with a higher degree might be more important in the pharmacological processes. The detailed parameters for these core targets are displayed in Supplementary Table 4. These data suggested that AKT1, SRC, JUN, TP53, EGFR, MYC, STAT1, ESR1, HSP90AA1, and CASP3 are probably the most important targets of YYD to suppress NSCLC.

### 3.4. Enrichment Assay and “Compound-Target-Pathway” Network

**Figure 4:**
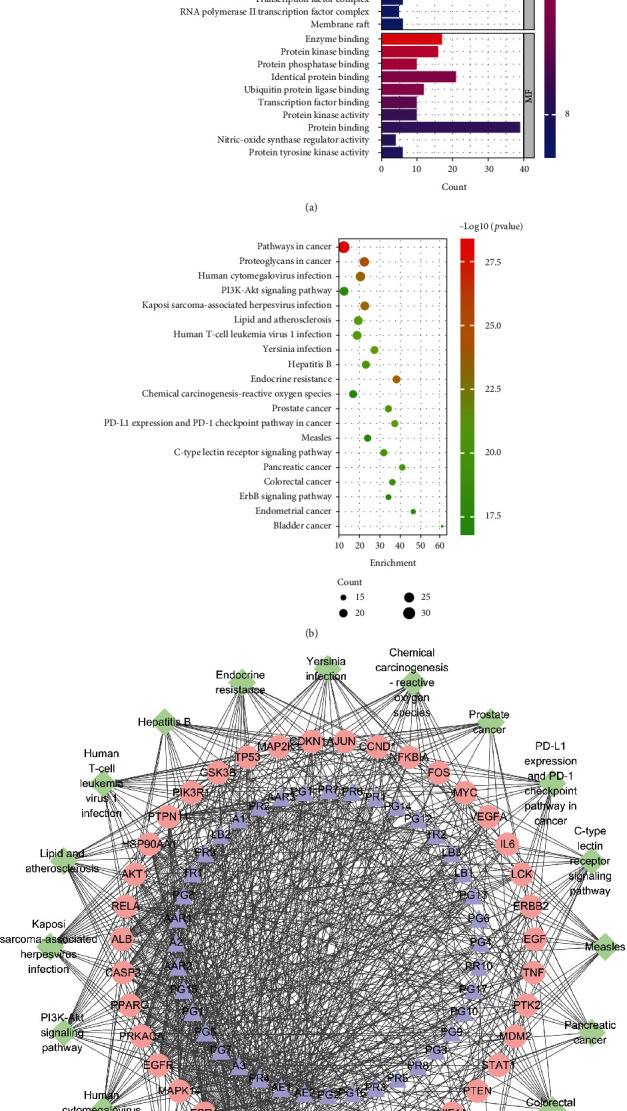
Biological process and pathway enrichment. (a, b) GO and KEGG analysis for the hub genes. (c) A “compound-target-pathway” network to disclose the interaction among the active components, core targets, and corresponding pathways.

To identify the biological characteristics of core targets of YYD against NSCLC, the Database for Annotation, Visualization, and Integrated Discovery (DAVID) was applied to perform GO enrichment analysis. With *p* < 0.05 and count ≥ 2, a total of 475 GO terms, including 367 for biological processes (BPs), 34 for cell components (CCs), and 74 for molecular functions (MFs), were obtained (Supplementary Table 5). The top ten BP, CC, and MF terms are exhibited in Figure 4(a), indicating that YYD might regulate cell apoptosis, response to drug, cellular response to hypoxia, and cell proliferation by enzyme binding, protein binding, and protein kinase binding in the cytosol, nucleus, and nucleoplasm. To further understand the potential pathways associated with the anticancer effects of YYD in NSCLC, KEGG analysis was performed for these core targets (Supplementary Table 6). The top 20 significantly enriched pathways of YYD against NSCLC were shown as a bubble chart (Figure 4(b)), including pathways in cancer, proteoglycans in cancer, PD-L1 expression and PD-1 checkpoint pathway in cancer, PI3K-AKT signaling pathway, chemical carcinogenesis-reactive oxygen species, and ErbB signaling pathway. To clarify the relationships among compounds, core targets, and signaling pathways, a “compound-target-pathway” network was built (Figure 4(c)). There existed 99 nodes (40 compounds, 39 targets, and 20 pathways) and 789 edges in the network, suggesting that YYD exerts anticancer activity in NSCLC through multiple compounds, targets, and pathways. AE2 (quercetin) and AE1 (luteolin) were regarded as the most important compounds. MAPK8, MAPK1, SRC, ESR1, MAPK14, EGFR, PRKACA, CASP3, PPARG, and ALB were identified as relatively high-degree targets. The PI3K-AKT signaling pathway was considered to play a vital role in the treatment of NSCLC. These data indicated that YYD might affect NSCLC cell proliferation and apoptosis by manipulating the PI3K-AKT pathway.

### 3.5. Clustering Analysis in the PPI Network

Cluster analysis was performed in the PPI network of 229 common targets, and a total of 7 topological modules were obtained (Supplementary Table 7). The most important 3 modules are shown in Supplementary Figure 1A. There were 50 targets and 343 edges in module 1, 32 targets and 123 edges in module 2, and 27 targets and 77 edges in module 3. Functionally, targets in module 1 were mainly enriched in cell cycle, cell proliferation, apoptosis, response to drug, and inflammatory response; targets in module 2 were mainly involved in cellular response to hypoxia, apoptosis, and response to drug; targets in module 3 were mainly associated with apoptosis, response to drug, NF-*κ*B transcription factor activity, cell proliferation, and inflammatory response (Supplementary Figure 1B). As described by KEGG analysis, targets in module 1 were mainly associated with pathways in cancer, PI3K-AKT signaling pathway, cellular senescence, TNF signaling pathway, and IL-17 signaling pathway; targets in module 2 were mainly related to pathways in cancer, p53 signaling pathway, and apoptosis; targets in module 2 were mainly correlated with pathways in cancer, Ras signaling pathway, MAPK signaling pathway, and apoptosis (Supplementary Figure 1C).

### 3.6. Molecular Docking

**Figure 5:**
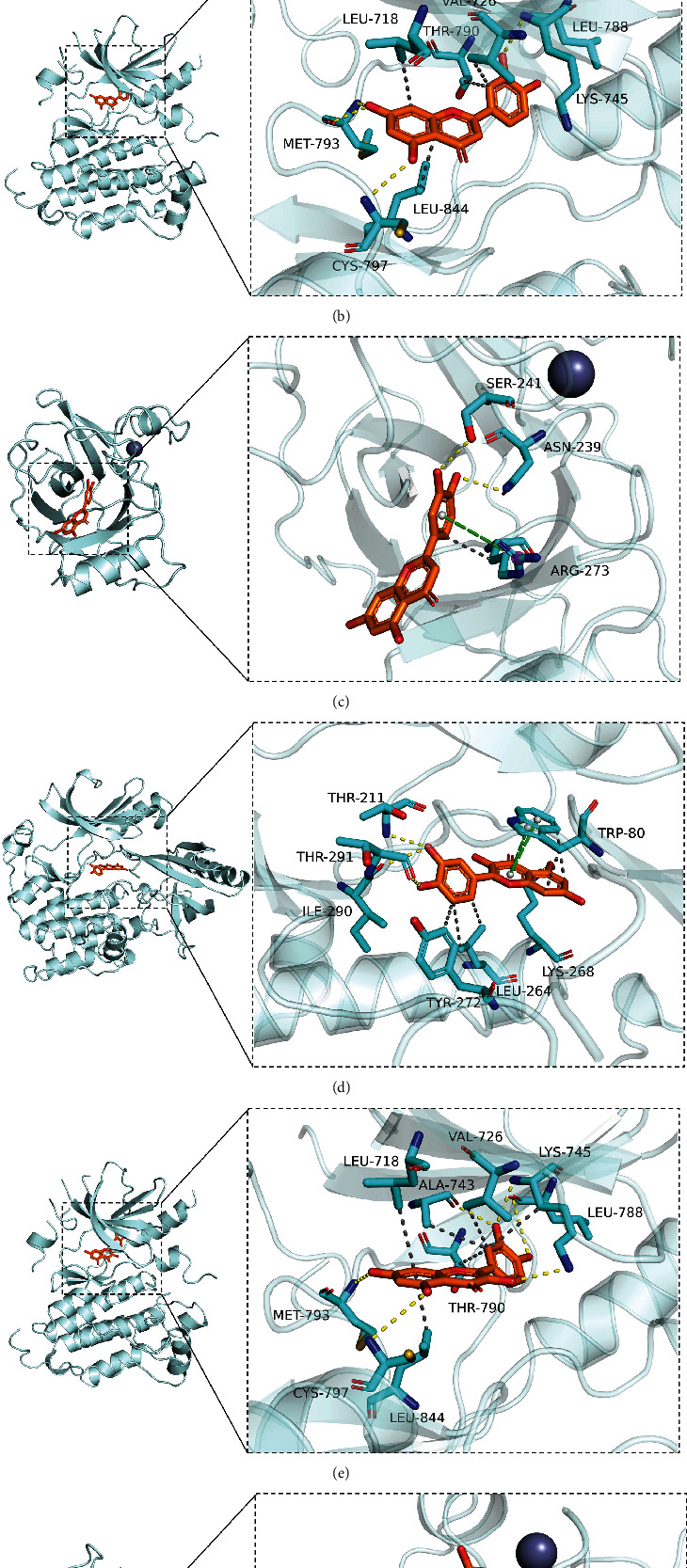
Molecular docking. (a–c) Molecular docking of luteolin and AKT1, EGFR, and TP53. (d–f) Molecular docking of quercetin and AKT1, EGFR, and TP53.

By comparing the top five targets in PPI network and the targets involved in the PI3K-AKT signaling pathway, three common targets (AKT1, TP53, and EGFR) were obtained. Then, molecular docking was performed between these 3 targets and the two most important compounds including AE2 (quercetin) and AE1 (luteolin). As shown in Figures 5(a)–5(c), luteolin possesses stable binding sites in AKT1, EGFR, and TP53 with the binding energy of −9.9, −8.9, and −6.8 kcal/mol. Similarly, quercetin can strongly bind to AKT1, EGFR, and TP53 with the binding energy of −9.9, −8.9, and −7.2 kcal/mol (Figures 5(d)–5(f)). Above all, we concluded that YYD might exert anticancer roles in NSCLC by targeting EGFR to deactivate PI3K-AKT signaling.

### 3.7. YYD Suppresses Cell Proliferation and Enhances Apoptosis in NSCLC

**Figure 6:**
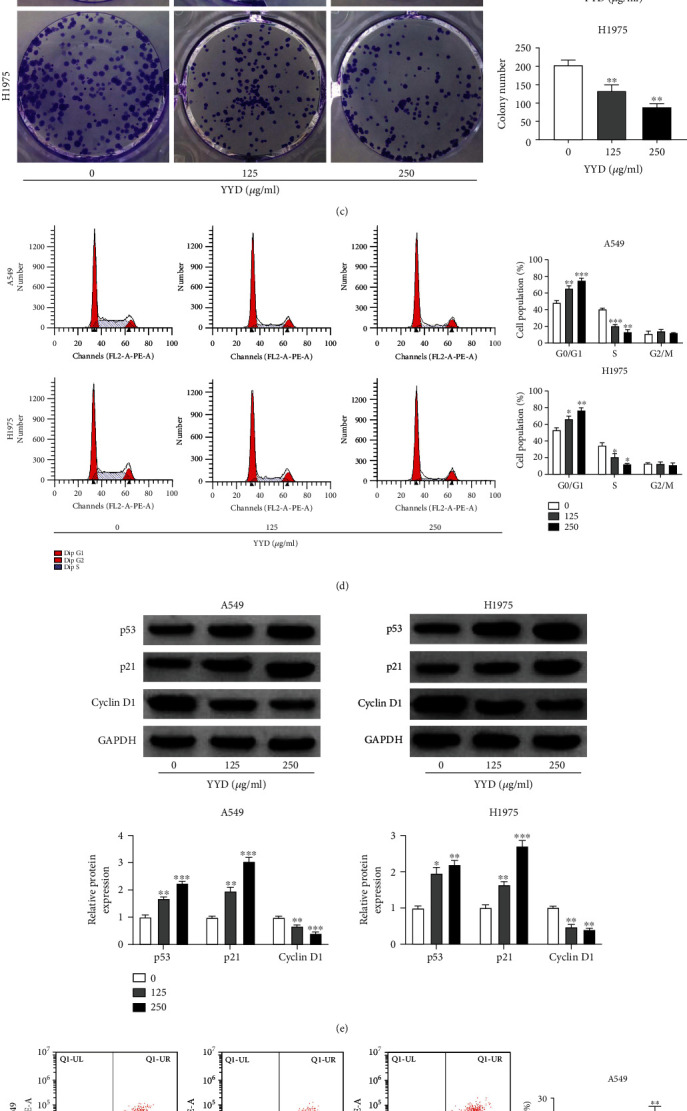
YYD suppresses NSCLC cell proliferation. (a) A549 and H1975 cells were treated with different doses of YYD for 24 or 48 h. Then, cell viability was assessed by CCK-8 assay. (b, c) EdU and colony formation assays were used to determine the cell proliferation in the presence of specified doses of YYD for 48 h. (d) After treatment with indicated concentrations of YYD for 48 h, cells were subjected to cell cycle analysis. (e) Western blot assays of p53, p21, and cyclin D1 in YYD-treated cells. (f) Flow cytometry analysis of apoptosis in cells treated with YYD. (g) Apoptosis-related proteins in YYD-treated cells were quantified by western blot. ^∗^*p* < 0.05, ^∗∗^*p* < 0.01, and ^∗∗∗^*p* < 0.001 versus the nontreated group.

To investigate the effects of YYD on NSCLC predicted by network pharmacological analysis, we performed a series of function experiments in A549 and H1975 cells treated with indicated doses of YYD for 24 h or 48 h. As demonstrated by the CCK-8 assay, YYD treatment resulted in a significant decrease of cell viability in a dose-dependent manner (Figure 6(a)). The IC_50_ values of YYD were 531.7 and 497.6 *μ*g/ml for A549 and H1975 cells at 24 h and 289.2 and 277.4 *μ*g/ml for A549 and H1975 cells at 48 h. Then, cells were maintained in a YYD-containing (0, 125, and 250 *μ*g/ml) medium for 48 h. EdU and colony forming assays further confirmed the decrease of EdU-positive cells and colony number with increasing concentrations of YYD (Figures 6(b) and 6(c)). As depicted by flow cytometry assay, YYD resulted in the increase of cells at the G0/G1 phase and the decrease of cells at the S phase (Figure 6(d)). Moreover, YYD treatment resulted in increased of p53 and p21 expression and decreased cyclin D1 expression (Figure 6(e)). As shown in Figure 6(f), YYD dose-dependently increased cell apoptosis. Consistently, increased Bax and cleaved caspase-3 protein expression and decreased Bcl-2 protein level were found in YYD-treated cells (Figure 6(g)). All these data suggested that YYD inhibited NSCLC cell proliferation and induced apoptosis.

### 3.8. YYD Inactivates EGFR-PI3K-AKT Signaling in NSCLC Cells

**Figure 7:**
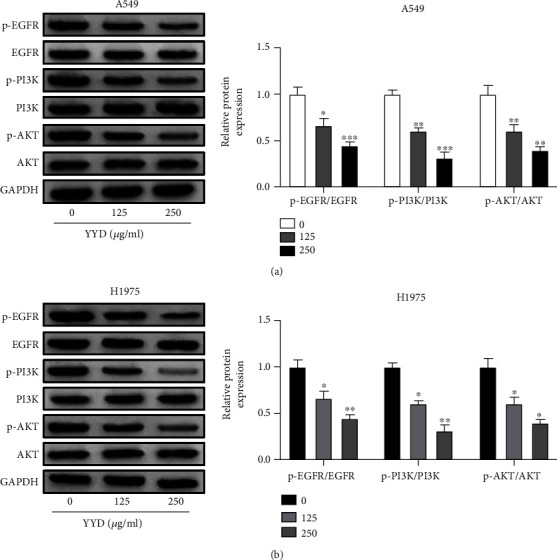
YYD inactivates EGFR-PI3K-AKT signaling in NSCLC cells. (a, b) Effects of YYD (0, 125, and 250 *μ*g/ml) on the expression levels of p-EGFR, EGFR, p-PI3K, PI3K, p-AKT, and AKT were determined with western blot assays. ^∗^*p* < 0.05, ^∗∗^*p* < 0.01, and ^∗∗∗^*p* < 0.001 versus the nontreated group.

As described by western blot, YYD dose-dependently repressed the phosphorylation levels of EGFR, PI3K, and AKT, but the levels of total EGFR, PI3K, and AKT were not changed (Figures 7(a) and 7(b)). Together, YYD led to the inactivation of EGFR-PI3K-AKT signaling in NSCLC.

### 3.9. YYD Inhibits NSCLC Cell Proliferation by Targeting EGFR to Deactivate PI3K-AKT Signaling

**Figure 8:**
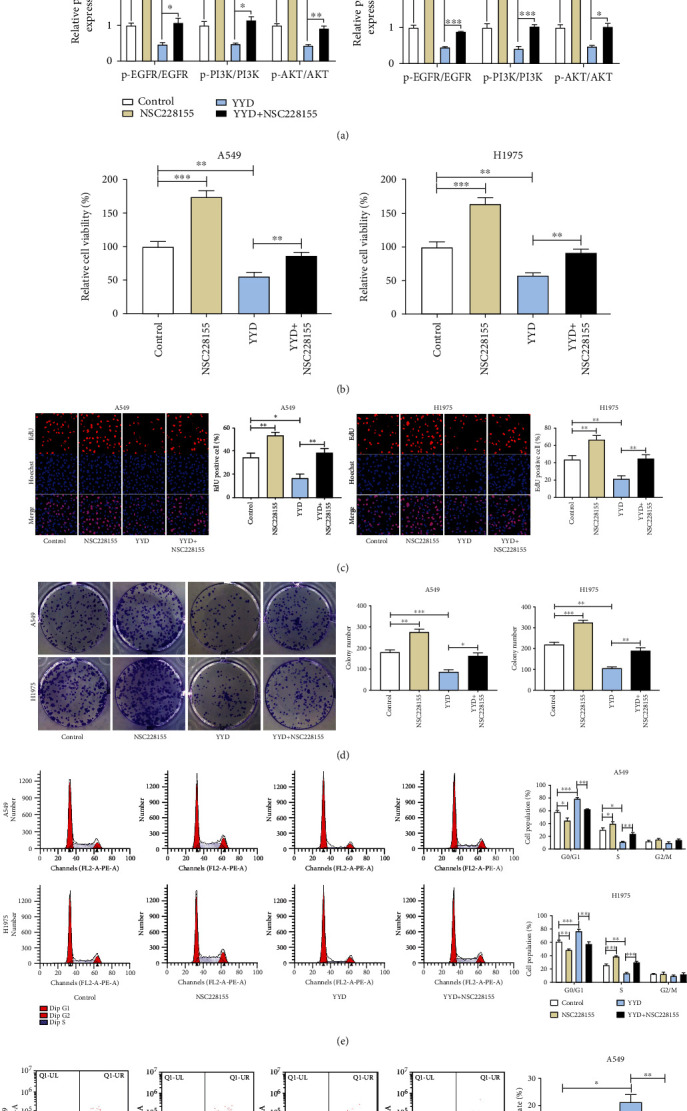
EGFR-PI3K-AKT signaling is responsible for the anticancer activity of YYD in NSCLC. Cells were treated with 250 *μ*g/ml YYD, 100 *μ*M NSC228155, or simultaneously treated with 250 *μ*g/ml YYD and 100 *μ*M NSC228155. (a) Western blot assays were performed to examine the related protein expression. (b–d) Cell proliferation capability was determined. (e, f) Cell cycle distribution and apoptosis were examined. ^∗^*p* < 0.05, ^∗∗^*p* < 0.01, and ^∗∗∗^*p* < 0.001 versus the control or YYD group.

Then, we further explored whether EGFR-PI3K-AKT signaling was implicated in the anticancer activity of YYD. Cells were treated with 250 *μ*g/ml YYD or 100 *μ*M NSC228155 (a specific EGFR agonist). As presented in Figure 8(a), the phosphorylation levels of EGFR, PI3K, and AKT were increased upon NSC228155 treatment. Moreover, YYD-induced decrease of p-EGFR, p-PI3K, and p-AKT expression was significantly weakened by NSC228155. Functionally, YYD-mediated suppression of cell proliferation was significantly attenuated due to EGFR activation (Figures 8(b)–8(d)). Consistently, the addition of EGFR agonists abated YYD-induced cell cycle arrest (Figure 8(e)). Furthermore, NSC228155 treatment abrogated YYD-induced apoptosis in NSCLC cells (Figure 8(f)). The above results suggested that YYD repressed NSCLC cell proliferation by inactivating the EGFR-PI3K-AKT pathway.

### 3.10. YYD Inhibits Tumor Growth *In Vivo*

**Figure 9:**
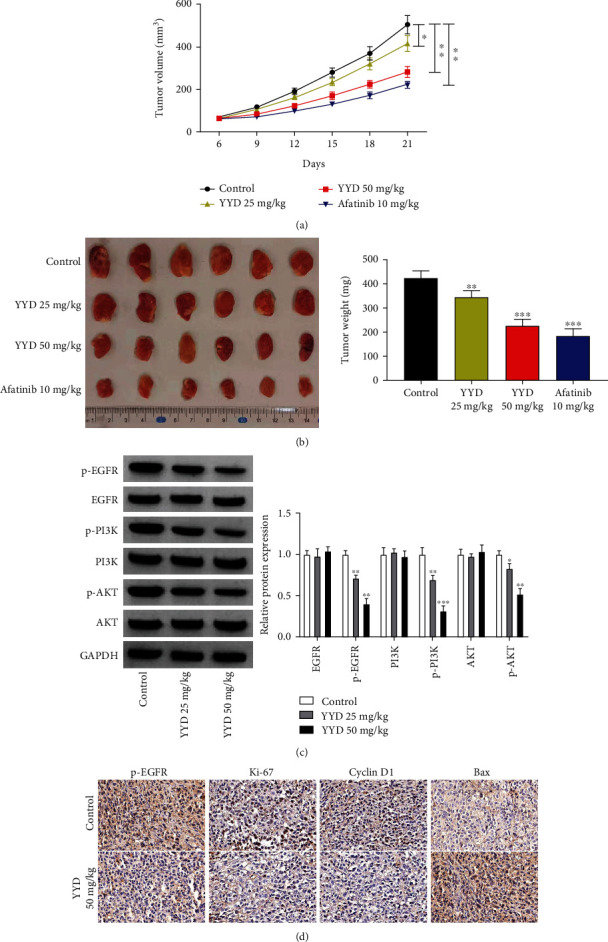
YYD inhibits NSCLC tumor growth. A549 cells were subcutaneously implanted into nude mice to establish the transplantation tumor models of NSCLC. After 6 days, mice were intragastrically administered with YYD daily. (a) Tumor growth was examined every 3 days. (b) Tumor weight. (c) The expression of proteins associated with EGFR-PI3K-AKT signaling. (d) IHC staining for p-EGFR, Ki-67, cyclin D1, and Bax in xenograft tumors. ^∗^*p* < 0.05, ^∗∗^*p* < 0.01, and ^∗∗∗^*p* < 0.001 versus the control group.

To investigate the antitumor effects of YYD in NSCLC *in vivo*, xenograft models were obtained in nude mice by inoculating A549 cells. Compared with the control group, YYD administration significantly decreased tumor size and weight in a dose-dependent manner albeit it was not as potent as afatinib (Figures 9(a) and 9(b)). Moreover, decreased p-EGFR, p-PI3K, and p-AKT expression was observed in xenograft tumors of YYD-treated mice (Figure 9(c)). IHC staining showed that YYD administration led to decreased p-EGFR, Ki-67, and cyclin D1 expression and increased Bax expression (Figure 9(d)). Together, YYD suppressed NSCLC tumorigenesis *in vivo*.

## 4. Discussion

Despite enormous survival benefits brought by comprehensive treatment, the overall cure and survival rates for NSCLC are still unsatisfactory [22]. TCM displays extensive pharmacological activities in human cancer by synergistic or contradictory effects among multiple compositions [23]. TCM is reported to exert anticancer activity in lung cancer by inducing apoptosis and/or autophagy, inhibiting metastasis, impacting immune reaction, and enhancing the therapeutic effect of EGFR-TKIs [24]. Network pharmacology is becoming a comprehensive and powerful approach to understanding TCM involving multiple herbs, multiple components, multitargets, and multipathways [25]. YYD has been found to improve the clinical symptoms of NSCLC patients; however, the detailed pharmacological mechanisms remain unknown, especially regarding its active compounds and key targets. Herein, we used network pharmacology integrated with experimental validation to elucidate the potential mechanism by which YYD inhibited NSCLC.

According to the TCMSP database, we retrieved a total of 40 bioactive compounds for YYD with OB ≥ 30%, DL ≥ 0.18, and HL ≥ 4. The “compound-target-disease” network showed that these 40 compounds might affect 229 NSCLC-related targets. Based on the network, AE2 (quercetin) was the most significant compound, followed by AE1 (luteolin) and A3 (kaempferol). Quercetin, a kind of flavonoid compound, is reported to exert antitumor activities by regulating the cell cycle, cell proliferation, apoptosis, angiogenesis, metastasis, and autophagy [26]. Quercetin has been widely discovered as a tumor suppressor and chemosensitizer in lung cancer [27–29]. Luteolin, a natural flavonoid found in multiple plants, prevents tumor development in various types of human malignancies by inhibiting several signals and transcription pathways essential for cancer cells [30]. Luteolin induces G2/M phase arrest and suppresses epithelial-mesenchymal transition (EMT) in NSCLC cells via downregulating AIM2 expression [31]. Luteolin repressed cell metastasis in lung cancer via Src/FAK and its downstream Rac1, Cdc42, and RhoA pathways [32]. Kaempferol, a well-characterized natural flavonol, is found to exert anticancer potential by regulating a series of cellular events via acting on intracellular or extracellular targets [33, 34]. Kaempferol promotes NSCLC cell apoptosis by downregulating Nrf2 mRNA expression and interfering Nrf2 downstream signaling [35]. Kaempferol represses TGF-*β*1-induced metastasis in lung cancer by inactivating AKT1-mediated phosphorylation of Smad3 [36]. Thus, YYD might suppress NSCLC progression by these main compounds.

Then, the PPI network was constructed to identify the key targets involved in the anti-NSCLC effects of YYD. Based on the degree values calculated by CytoNCA, AKT1, SRC, JUN, TP53, and EGFR were screened as the most potential targets to combat NSCLC. GO and KEGG assays demonstrated that YYD might affect cell apoptosis and proliferation by the PI3K-AKT signaling pathway, PD-L1 expression and PD-1 checkpoint pathway in cancer, and ErbB signaling pathway. By comparing the top five targets in the PPI network and the targets implicated in the PI3K-AKT signaling pathway, we acquired three common targets (AKT1, TP53, and EGFR). Moreover, molecular docking analysis indicated the strong binding between these 3 targets and the two most important compounds including AE2 (quercetin) and AE1 (luteolin). AKT serine/threonine kinase serves as an oncogenic protein, and its phosphorylation mediates the function of different downstream proteins associated with cellular proliferation, metabolism, metastasis, and angiogenesis [37]. AKT is a key node associated with multiple signaling pathways, including the PI3K signaling [38]. EGFR, a transmembrane receptor tyrosine kinase, is overexpressed in many solid tumors, such as lung cancer, breast cancer, and glioblastoma [39]. The overexpression or overactivation of EGFR leads to the stimulation of its downstream signaling cascades, including the PI3K-AKT pathway, which induces cell growth, cell cycle progression, cell motility, and angiogenesis and blocks apoptosis [40]. The hyperactivation of the PI3K/AKT pathway is observed in different tumors and is strongly linked to tumorigenesis, immune microenvironment, and chemoresistance of cancer cells [41]. According to the data from network pharmacology analysis and literature researches, we concluded that YYD might affect cell proliferation and apoptosis by targeting EGFR to inactivate the PI3K-AKT signaling pathway.

Subsequently, the effects of YYD on NSCLC cell proliferation and apoptosis were validated. The *in vitro* results showed that YYD treatment reduced cell viability, EdU-positive cells, and colony formation ability, induced cell cycle arrest, and enhanced apoptosis in a dose-dependent manner. Moreover, cell cycle-related proteins p53 and p21 were increased, and cyclin D1 was decreased due to the increase of YYD concentration. Apoptosis-related proteins cleaved caspase-3 and Bax were increased, and Bcl-2 was decreased in YYD-treated cells. The *in vivo* experiments also confirmed the suppressive effect of YYD administration on tumor growth. These data certified the antiproliferative and proapoptotic activity of YYD in NSCLC.

The EGFR-PI3K-AKT signaling is reported to be implicated in the occurrence and development of various malignant tumors, including NSCLC. For example, SKA3 promotes lung adenocarcinoma metastasis by binding to EFGR and activating PI3K-AKT signaling [42]. JMJD8 exerts carcinogenic activity in NSCLC cells by maintaining EGFR stability and stimulating the downstream PI3K/AKT signaling pathway [43]. Cyclooxygenase-2 induces gefitinib resistance in NSCLC through the EGFR/PI3K/AKT axis [44]. Here, we found that YYD treatment resulted in the suppression of EGFR-PI3K-AKT signaling. Moreover, activation of EGFR by NSC228155 significantly reversed the inhibition of YYD on cell proliferation and apoptosis. These data suggested that YYD suppressed NSCLC cell proliferation and promoted apoptosis by inactivating the EGFR-PI3K-AKT signaling pathway. There are also several limitations to this study. First, the effects of YYD on NSCLC cell migration and invasion were not discussed. Second, the relationship between YYD and chemotherapeutic sensitivity needs to be further addressed. Yiqi Yangyin Decoction has clinically been used to inhibit NSCLC progression in our hospital and has obtained some very good curative effect. In our future research, we will further optimize the drug composition. Also, we will try to explore the combination administration of Yiqi Yangyin Decoction and some other chemotherapeutic agents or targeted drugs in NSCLC patients.

In conclusion, our results showed that YYD suppressed NSCLC cell proliferation and promoted apoptosis *in vitro* and hindered tumor growth *in vivo*. Moreover, EGFR-PI3K-AKT signaling was demonstrated to be responsible for the anticancer activity of YYD in NSCLC. Combination of the network pharmacology method and experimental validation in this study provides a powerful tool to understand the action mechanism of TCM.

## Figures and Tables

**Table 1:** Active compounds of YYD.

Mark	Compound name	MOL ID	OB (%)	DL	HL	Herb
A1	Stigmasterol	MOL000449	43.83	0.76	5.57	PG/OJ/LB
A2	Beta-sitosterol	MOL000358	36.91	0.75	5.36	PG/PR/LB/AAR
A3	Kaempferol	MOL000422	41.88	0.24	14.74	PG/AE
PG1	Chrysanthemaxanthin	MOL004492	38.72	0.58	17.47	PG
PG2	Celabenzine	MOL005314	101.88	0.49	8.15	PG
PG3	Deoxyharringtonine	MOL005317	39.27	0.81	7.9	PG
PG4	Dianthramine	MOL005318	40.45	0.2	5.14	PG
PG5	Arachidonate	MOL005320	45.57	0.2	7.56	PG
PG6	Frutinone A	MOL005321	65.9	0.34	19.1	PG
PG7	Ginsenoside rh2	MOL005344	36.32	0.56	11.08	PG
PG8	Ginsenoside-Rh4_qt	MOL005348	31.11	0.78	6.97	PG
PG9	Girinimbin	MOL005356	61.22	0.31	8.17	PG
PG10	Gomisin B	MOL005357	31.99	0.83	7.81	PG
PG11	Malkangunin	MOL005360	57.71	0.63	4.09	PG
PG12	Panaxadiol	MOL005376	33.09	0.79	6.34	PG
PG13	Suchilactone	MOL005384	57.52	0.56	9.03	PG
PG14	Alexandrin_qt	MOL005399	36.91	0.75	5.53	PG
PG15	Ginsenoside Rg5_qt	MOL005401	39.56	0.79	5.65	PG
PG16	Fumarine	MOL000787	59.26	0.83	23.46	PG
PG17	Inermin	MOL003648	65.83	0.54	11.73	PG
AE1	Luteolin	MOL000006	36.16	0.25	15.94	AE
AE2	Quercetin	MOL000098	46.43	0.28	14.4	AE
TR1	Spinasterol	MOL004355	42.98	0.76	5.32	TR
TR2	Schottenol	MOL006756	37.42	0.75	5.63	TR
PR1	Sitosterol	MOL000359	36.91	0.75	5.37	PR
PR2	Methylprotodioscin_qt	MOL003889	35.12	0.86	5.48	PR
PR3	(2R)-7-Hydroxy-2-(4-hydroxyphenyl)chroman-4-one	MOL004941	71.12	0.18	18.09	PR
PR4	Diosgenin	MOL000546	80.88	0.81	4.14	PR
PR5	4′,5-Dihydroxyflavone	MOL006331	48.55	0.19	18.01	PR
PR6	Sibiricoside A_qt	MOL009760	35.26	0.86	5.44	PR
PR7	Zhonghualiaoine 1	MOL009766	34.72	0.78	5.25	PR
PR8	Liquiritigenin	MOL001792	32.76	0.18	17.89	PR
PR9	Baicalein	MOL002714	33.52	0.21	16.25	PR
PR10	3′-Methoxydaidzein	MOL002959	48.57	0.24	17.04	PR
LB1	26-Dihydroxy-choleslen-16	MOL009449	32.43	0.8	6.02	LB
LB2	26-Dihydroxy-5-cholesten-16	MOL009465	35.11	0.81	4.15	LB
LB3	26-Dihydroxy-cholestan-16	MOL009471	32.43	0.8	5.73	LB
AAR1	Mandenol	MOL001494	42	0.19	5.39	AAR
AAR2	Ethyl oleate (NF)	MOL002883	32.4	0.19	4.85	AAR
AAR3	Cycloartenol acetate	MOL003479	41.11	0.8	6.9	AAR

## Data Availability

The datasets used and/or analyzed during the current study are available from the corresponding author on reasonable request.

## Supplementary Materials

Supplementary 1 Supplementary Figure 1: cluster analysis for putative genes of YYD against NSCLC. (A) Cluster analysis of PPI network. (B) The GO-BP analysis for core targets in different modules. (C) The KEGG pathway enrichment analysis for hub genes in different modules.

Supplementary 2 Supplementary Table 1: target genes of 40 bioactive components in YYD.

Supplementary 3 Supplementary Table 2: targets associated with NSCLC.

Supplementary 4 Supplementary Table 3: the overlapping genes for compound targets and NSCLC-related targets.

Supplementary 5 Supplementary Table 4: parameters of the core genes in PPI network.

Supplementary 6 Supplementary Table 5: the detailed information for GO terms with *p* < 0.05 and count ≥ 2.

Supplementary 7 Supplementary Table 6: the detailed pathway information of YYD against NSCLC.

Supplementary 8 Supplementary Table 7: cluster analysis for PPI network.
