# Supplementary figures and images for: Integrating Network Pharmacology and Experimental Validation to Elucidate the Mechanism of Yiqi Yangyin Decoction in Suppressing Non-Small-Cell Lung Cancer
Source: Biomed Res Int. 2023 Feb 20;2023:4967544. doi: 10.1155/2023/4967544 (PMC9980286; doi:10.1155/2023/4967544)

A

Module 1

Module 2

Module 3

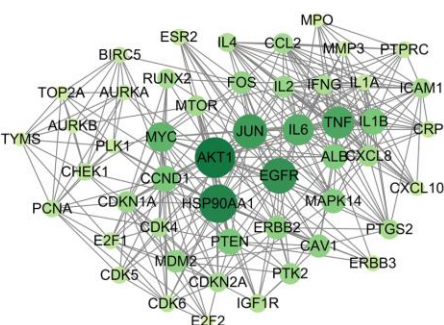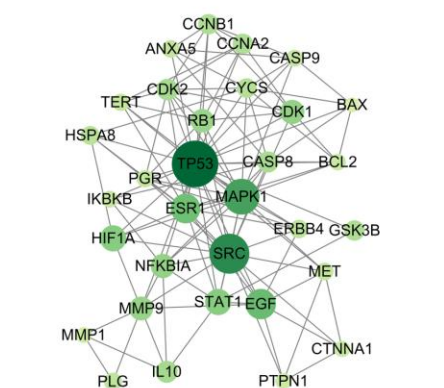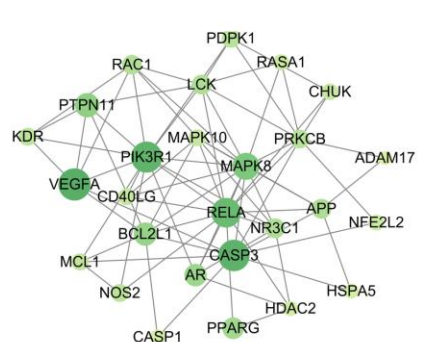

B

Module 1

Module 2

Module 3

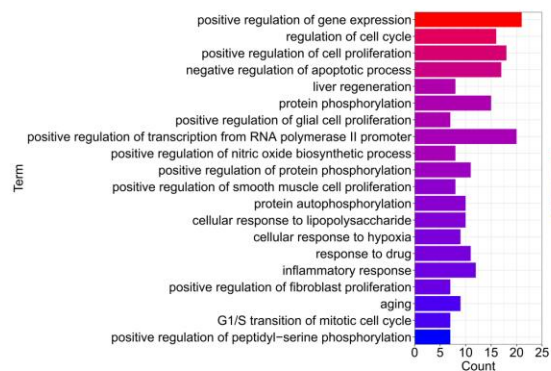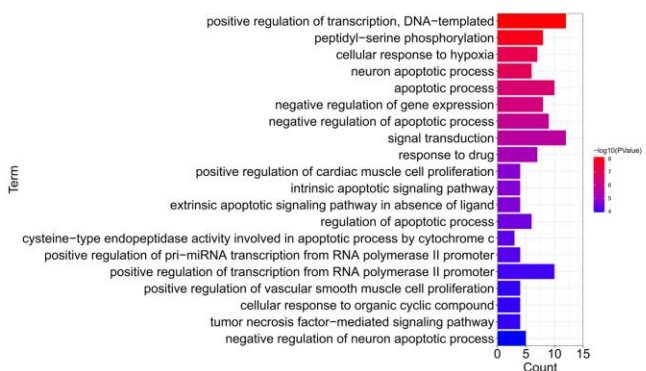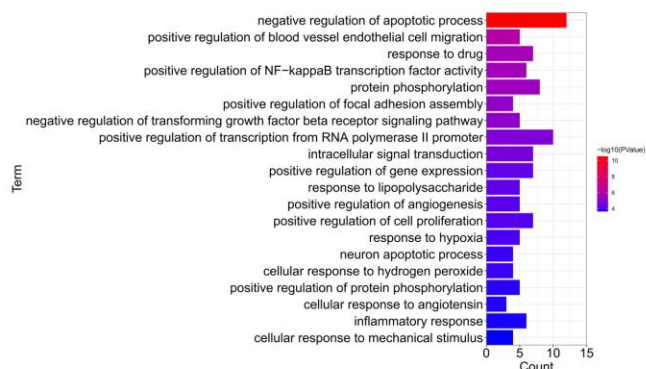

C

Module 1

Module 2

Module 3

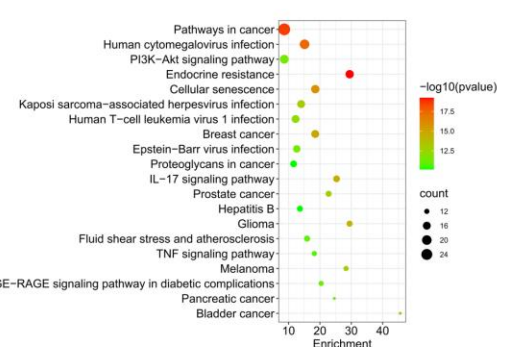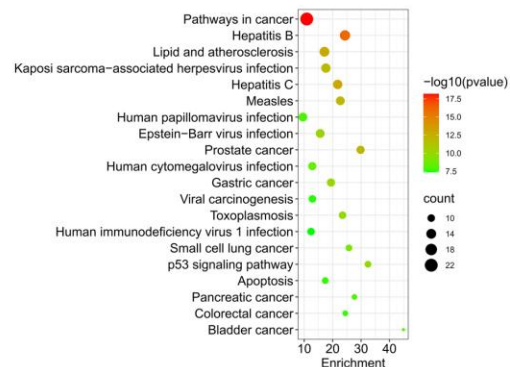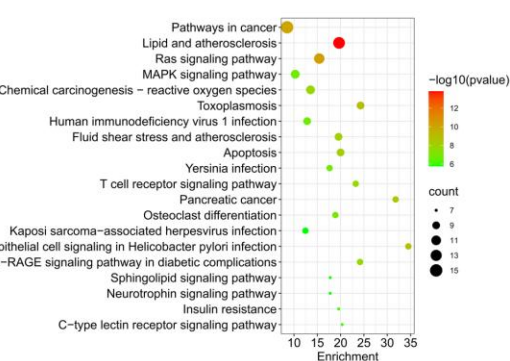

Supplement: Supplementary 1 — Supplementary Figure 1: cluster analysis for putative genes of YYD against NSCLC. (A) Cluster analysis of PPI network. (B) The GO-BP analysis for core targets in different modules. (C) The KEGG pathway enrichment analysis for hub genes in different modules. [file 4967544.f1.pdf]
