# Supplementary material for: Integrating Network Pharmacology and Experimental Validation to Elucidate the Mechanism of Yiqi Yangyin Decoction in Suppressing Non-Small-Cell Lung Cancer
Source: Biomed Res Int. 2023 Feb 20;2023:4967544. doi: 10.1155/2023/4967544 (PMC9980286; doi:10.1155/2023/4967544)
Supplement: Supplementary 2 — Supplementary Table 1: target genes of 40 bioactive components in YYD. [file 4967544.f2.pdf]

Supplementay Table 1: Target genes of 40 bioactive components in YYD

Number	Protein name	Gene name
1	Progesterone receptor	PGR
2	Mineralocorticoid receptor	NR3C2
3	Nuclear receptor coactivator 2	NCOA2
4	Alcohol dehydrogenase 1C	ADH1C
5	Ig gamma-1 chain C region	IGHG1
6	Retinoic acid receptor RXR-alpha	RXRA
7	Nuclear receptor coactivator 1	NCOA1
8	Prostaglandin G/H synthase 1	PTGS1
9	Prostaglandin G/H synthase 2	PTGS2
10	Alpha-2A adrenergic receptor	ADRA2A
11	Sodium-dependent noradrenaline transporter	SLC6A2
12	Sodium-dependent dopamine transporter	SLC6A3
13	Beta-2 adrenergic receptor	ADRB2
14	Aldose reductase	AKR1B1
15	Urokinase-type plasminogen activator	PLAU
16	Leukotriene A-4 hydrolase	LTA4H
17	Amine oxidase [flavin-containing] B	MAOB
18	Amine oxidase [flavin-containing] A	MAOA
19	mRNA of PKA Catalytic Subunit C-alpha	PRKACA
20	Chymotrypsinogen B	CTRB1
21	Muscarinic acetylcholine receptor M3	CHRM3
22	Muscarinic acetylcholine receptor M1	CHRM1
23	Beta-1 adrenergic receptor	ADRB1
24	Sodium channel protein type 5 subunit alpha	SCN5A
25	5-hydroxytryptamine 2A receptor	HTR2A
26	Alpha-1A adrenergic receptor	ADRA1A
27	Gamma-aminobutyric-acid receptor alpha-3 subunit	GABRA3
28	Muscarinic acetylcholine receptor M2	CHRM2
29	Alpha-1B adrenergic receptor	ADRA1B
30	Gamma-aminobutyric acid receptor subunit alpha-1	GABRA1
31	Neuronal acetylcholine receptor protein, alpha-7 chain	CHRNA7
32	Cytochrome P450 51 (by homology)	CYP51A1
33	HMG-CoA reductase	HMGCR
34	LXR-alpha	NR1H3
35	Niemann-Pick C1-like protein 1	NPC1L1
36	Cytochrome P450 17A1	CYP17A1
37	3-oxo-5-alpha-steroid 4-dehydrogenase 2	SRD5A2
38	Estrogen receptor	ESR1
39	Androgen receptor	AR
40	3-oxo-5-alpha-steroid 4-dehydrogenase 1	SRD5A1
41	Glucocorticoid receptor	NR3C1
42	G-protein coupled bile acid receptor 1	GPBAR1
43	M-phase inducer phosphatase 2	CDC25B
44	5-hydroxytryptamine receptor 1E	HTR1E

45	Arachidonate 15-lipoxygenase	ALOX15
46	Estrogen receptor beta	ESR2
47	Aromatase	CYP19A1
48	Kappa-type opioid receptor	OPRK1
49	Phospholipase A2	PLA2G1B
50	Aldo-keto reductase family 1 member C2	AKR1C2
51	MAP kinase-activated protein kinase 2	MAPKAPK2
52	Bone morphogenetic protein 2	BMP2
53	Proto-oncogene serine/threonine-protein kinase Pim-1	PIM1
54	Steryl-sulfatase	STS
55	Serum albumin	ALB
56	Apolipoprotein A-II	APOA2
57	Thyroid hormone receptor beta	THRB
58	Caspase-7	CASP7
59	Carbonic anhydrase 2	CA2
60	Nuclear receptor ROR-alpha	RORA
61	Kinesin-like protein KIF11	KIF11
62	Estradiol 17-beta-dehydrogenase 11	HSD17B11
63	Vitamin D-binding protein	GC
64	Mitogen-activated protein kinase 1	MAPK1
65	Transthyretin	TTR
66	SEC14-like protein 2	SEC14L2
67	Collagenase 3	MMP13
68	Estradiol 17-beta-dehydrogenase 1	HSD17B1
69	Integrin alpha-L	ITGAL
70	Aldo-keto reductase family 1 member C3	AKR1C3
71	Bile acid receptor	NR1H4
72	Corticosteroid 11-beta-dehydrogenase isozyme 1	HSD11B1
73	Cell division protein kinase 2	CDK2
74	Proto-oncogene tyrosine-protein kinase Src	SRC
75	cAMP-specific 3,5-cyclic phosphodiesterase 4B	PDE4B
76	ADAM 17	ADAM17
77	Caspase-3	CASP3
78	Coagulation factor X	F10
79	Tyrosine-protein phosphatase non-receptor type 11	PTPN11
80	Peroxisome proliferator-activated receptor gamma	PPARG
81	Bile salt sulfotransferase	SULT2A1
82	Epidermal growth factor receptor	EGFR
83	Mitogen-activated protein kinase 8	MAPK8
84	Mitogen-activated protein kinase 14	MAPK14
85	Annexin A5	ANXA5
86	Dihydroorotate dehydrogenase, mitochondrial	DHODH
87	Glutathione reductase, mitochondrial	GSR
88	Peptidyl-prolyl cis-trans isomerase A	PPIA
89	S-methyl-5-thioadenosine phosphorylase	MTAP
90	Beta-secretase 1	BACE1

91	B-Raf proto-oncogene serine/threonine-protein kinase	BRAF
92	Serine/threonine-protein phosphatase 5	PPP5C
93	Sex hormone-binding globulin	SHBG
94	Nitric oxide synthase, endothelial	NOS3
95	Vascular endothelial growth factor receptor 2	KDR
96	Dihydrofolate reductase	DHFR
97	Troponin C, slow skeletal and cardiac muscles	TNNC1
98	Heat shock protein HSP 90	HSP90AA1
99	Phosphatidylinositol-4,5-bisphosphate 3-kinase catalytic subunit, gamn	PIK3CG
100	Potassium voltage-gated channel subfamily H member 2	KCNH2
101	Dopamine D1 receptor	DRD5
102	Gamma-aminobutyric-acid receptor alpha-2 subunit	GABRA2
103	Muscarinic acetylcholine receptor M4	CHRM4
104	CGMP-inhibited 3',5'-cyclic phosphodiesterase A	PDE3A
105	Gamma-aminobutyric-acid receptor alpha-5 subunit	GABRA5
106	Neuronal acetylcholine receptor subunit alpha-2	CHRNA2
107	Sodium-dependent serotonin transporter	SLC6A4
108	Mu-type opioid receptor	OPRM1
109	Apoptosis regulator Bcl-2	BCL2
110	Apoptosis regulator BAX	BAX
111	Caspase-9	CASP9
112	Transcription factor AP-1	JUN
113	Caspase-8	CASP8
114	Protein kinase C alpha type	PRKCA
115	Transforming growth factor beta-1	TGFB1
116	Serum paraoxonase/arylesterase 1	PON1
117	Microtubule-associated protein 2	MAP2
118	Acetylcholinesterase	ACHE
119	Receptor-interacting serine/threonine-protein kinase 2	RIPK2
120	Prothrombin	F2
121	Histone deacetylase 8	HDAC8
122	Wiskott-Aldrich syndrome protein	WAS
123	Phenylethanolamine N-methyltransferase	PNMT
124	3-phosphoinositide-dependent protein kinase 1	PDPK1
125	Liver carboxylesterase 1	CES1
126	Serine/threonine-protein kinase Chk1	CHEK1
127	Trafficking protein particle complex subunit 3	TRAPPC3
128	TGF-beta receptor type-1	TGFBR1
129	Nitric oxide synthase, inducible	NOS2
130	Dipeptidyl peptidase IV	DPP4
131	Trypsin-1	PRSS1
132	DNA topoisomerase II	TOP2A
133	Coagulation factor VII	F7
134	Calmodulin	CAMKKA
135	Transcription factor p65	RELA
136	Inhibitor of nuclear factor kappa-B kinase subunit beta	IKBKB

137	RAC-alpha serine/threonine-protein kinase	AKT1
138	Tumor necrosis factor	TNF
139	Activator of 90 kDa heat shock protein ATPase homolog 1	AHSA1
140	Xanthine dehydrogenase/oxidase	XDH
141	Interstitial collagenase	MMP1
142	Signal transducer and activator of transcription 1-alpha/beta	STAT1
143	Cell division control protein 2 homolog	CDK1
144	Heme oxygenase 1	HMOX1
145	Cytochrome P450 3A4	CYP3A4
146	Cytochrome P450 1A2	CYP1A2
147	Cytochrome P450 1A1	CYP1A1
148	Intercellular adhesion molecule 1	ICAM1
149	E-selectin	SELE
150	Vascular cell adhesion protein 1	VCAM1
151	Nuclear receptor subfamily 1 group I member 2	NR1I2
152	Cytochrome P450 1B1	CYP1B1
153	Arachidonate 5-lipoxygenase	ALOX5
154	Hyaluronan synthase 2	HAS2
155	Glutathione S-transferase P	GSTP1
156	Aryl hydrocarbon receptor	AHR
157	26S proteasome non-ATPase regulatory subunit 3	PSMD3
158	Solute carrier family 2, facilitated glucose transporter member 4	SLC2A4
159	Nuclear receptor subfamily 1 group I member 3	NR1I3
160	Insulin receptor	INSR
161	Type I iodothyronine deiodinase	DIO1
162	Serine/threonine-protein phosphatase 2B catalytic subunit alpha isofor	PPP3CA
163	Glutathione S-transferase Mu 1	GSTM1
164	Glutathione S-transferase Mu 2	GSTM2
165	Antileukoproteinase	SLPI
166	NADPH oxidase 4	NOX4
167	Tyrosinase	TYR
168	Tyrosine-protein kinase receptor FLT3	FLT3
169	Carbonic anhydrase VII	CA7
170	Estradiol 17-beta-dehydrogenase 2	HSD17B2
171	Multidrug resistance-associated protein 1	ABCC1
172	Carbonic anhydrase XII	CA12
173	Estrogen-related receptor alpha	ESRRA
174	P-glycoprotein 1	ABCB1
175	ATP-binding cassette sub-family G member 2	ABCG2
176	Adenosine A1 receptor (by homology)	ADORA1
177	Carbonic anhydrase IV	CA4
178	Glyoxalase I	GLO1
179	Tyrosine-protein kinase SYK	SYK
180	Glycogen synthase kinase-3 beta	GSK3B
181	Matrix metalloproteinase 9	MMP9
182	Matrix metalloproteinase 2	MMP2

183	Arachidonate 12-lipoxygenase	ALOX12
184	Receptor-type tyrosine-protein phosphatase S	PTPRS
185	Adenosine A2a receptor (by homology)	ADORA2A
186	Cyclin-dependent kinase 5	CDK5
187	CDK5 activator 1	CDK5R1
188	cyclin B3	CCNB3
189	cyclin B1	CCNB1
190	cyclin B2	CCNB2
191	Arginase-1 (by homology)	ARG1
192	G-protein coupled receptor 35	GPR35
193	Death-associated protein kinase 1	DAPK1
194	DNA-3-methyladenine glycosylase	MPG
195	Solute carrier family 22 member 12	SLC22A12
196	Receptor-type tyrosine-protein phosphatase C	PTPRC
197	Tyrosine-protein phosphatase non-receptor type 22	PTPN22
198	Induced myeloid leukemia cell differentiation protein Mcl-1	MCL1
199	Dual specificity protein phosphatase 3	DUSP3
200	Carbonic anhydrase 5A, mitochondrial	CA5A
201	Macrophage migration inhibitory factor	MIF
202	Polyphenol oxidase 2	PPO2
203	Cocaine esterase	CES2
204	Bcl-2-related protein A1	BCL2A1
205	Ribosomal protein S6 kinase alpha-3	RPS6KA3
206	Carbonic anhydrase 13	CA13
207	Telomerase reverse transcriptase	TERT
208	Carbonic anhydrase 5B, mitochondrial	CA5B
209	Carbonic anhydrase 6	CA6
210	Alkaline phosphatase, tissue-nonspecific isozyme	ALPL
211	Toll-like receptor 9	TLR9
212	Testosterone 17-beta-dehydrogenase 3	HSD17B3
213	Carbonic anhydrase 9	CA9
214	DNA (cytosine-5)-methyltransferase 1	DNMT1
215	Carbonic anhydrase 14	CA14
216	Tubulin beta-2B chain	TUBB2B
217	Carboxy-terminal domain RNA polymerase II polypeptide A small pho	CTDSP1
218	Tubulin alpha-1A chain	TUBA1A
219	Tyrosine-protein phosphatase non-receptor type 1	PTPN1
220	Glycogen synthase kinase-3 alpha	GSK3A
221	High affinity cAMP-specific 3',5'-cyclic phosphodiesterase 7A	PDE7A
222	Hepatocyte nuclear factor 4-alpha	HNF4A
223	Ras-related C3 botulinum toxin substrate 1	RAC1
224	Mitogen-activated protein kinase 10	MAPK10
225	Cytochrome P450 2C9	CYP2C9
226	Protein kinase C epsilon type	PRKCE
227	Glutamate receptor ionotropic, NMDA 2B	GRIN2B
228	Thyroid hormone receptor alpha	THRA

229	Hydroxycarboxylic acid receptor 2	HCAR2
230	Amyloid beta A4 protein	APP
231	Tyrosine-protein kinase HCK	HCK
232	Cholinesterase	BCHE
233	Glucosylceramidase	GBA
234	Cell division protein kinase 6	CDK6
235	Angiogenin	ANG
236	Branched-chain-amino-acid aminotransferase, mitochondrial	BCAT2
237	Heat shock cognate 71 kDa protein	HSPA8
238	Complement factor B	CFB
239	Oxysterols receptor LXR-beta	NR1H2
240	NAD(P)H dehydrogenase [quinone] 1	NQO1
241	Carbonic anhydrase 1	CA1
242	Complement C1r subcomponent	C1R
243	Serine/threonine-protein kinase 6	AURKA
244	Retinoic acid receptor beta	RARB
245	Retinoic acid receptor gamma	RARG
246	Retinoic acid receptor RXR-beta	RXRB
247	Retinoic acid receptor RXR-gamma	RXRG
248	Retinoic acid receptor alpha	RARA
249	Protein kinase C gamma type	PRKCG
250	Potassium voltage-gated channel subfamily A member 3	KCNA3
251	Peptidyl-prolyl cis-trans isomerase FKBP1A	FKBP1A
252	Stromelysin-1	MMP3
253	cAMP-specific 3,5-cyclic phosphodiesterase 4D	PDE4D
254	Cyclin-A2	CCNA2
255	Flavin reductase	BLVRB
256	Ribosyldihydronicotinamide dehydrogenase [quinone]	NQO2
257	Ephrin type-B receptor 4	EPHB4
258	E3 ubiquitin-protein ligase Mdm2	MDM2
259	Alpha-tocopherol transfer protein	TTPA
260	Aldo-keto reductase family 1 member C1	AKR1C1
261	Receptor tyrosine-protein kinase erbB-4	ERBB4
262	Dual specificity protein phosphatase 6	DUSP6
263	Cathepsin K	CTSK
264	Peroxisome proliferator-activated receptor delta	PPARD
265	Proto-oncogene tyrosine-protein kinase LCK	LCK
266	Poly [ADP-ribose] polymerase 1	PARP1
267	Protein farnesyltransferase/geranylgeranyltransferase type-1 subunit alp	FNTA
268	Methionine aminopeptidase 2	METAP2
269	Purine nucleoside phosphorylase	PNP
270	Serine/threonine-protein kinase PLK1	PLK1
271	Adenosine kinase	ADK
272	Insulin-like growth factor 1 receptor	IGF1R
273	Fatty acid-binding protein, adipocyte	FABP4
274	Complement factor D	CFD

275	Estrogen-related receptor gamma	ESRRG
276	Thymidylate synthase	TYMS
277	Glutathione S-transferase A1	GSTA1
278	Alcohol dehydrogenase 1B	ADH1B
279	Alcohol dehydrogenase class-3	ADH5
280	Fatty acid-binding protein, heart	FABP3
281	Prolyl endopeptidase	PREP
282	CHRNA7-FAM7A fusion protein	CHRFAM7A
283	Trace amine-associated receptor 1	TAAR1
284	Smoothened homolog	SMO
285	COUP transcription factor 2	NR2F2
286	Neuronal acetylcholine receptor subunit alpha-4	CHRNA4
287	Voltage-dependent N-type calcium channel subunit alpha-1B	CACNA1B
288	Interleukin-1 receptor-associated kinase 4	IRAK4
289	Histamine H3 receptor	HRH3
290	Putative ATP-dependent Clp protease proteolytic subunit, mitochondri	CLPP
291	Muscarinic acetylcholine receptor M5	CHRM5
292	Perilipin-1	PLIN1
293	P-selectin	SELP
294	Probable ATP-dependent RNA helicase DDX6	DDX6
295	Triggering receptor expressed on myeloid cells 1	TREM1
296	Cathepsin D	CTSD
297	Alpha-amylase 1	AMY1A
298	Tryptase beta-2	TPSB2
299	Chymase	CMA1
300	SPARC	SPARC
301	Seprase	FAP
302	Glutaminyl-peptide cyclotransferase	QPCT
303	Neutrophil gelatinase-associated lipocalin	LCN2
304	Lanosterol synthase	LSS
305	Cathepsin S	CTSS
306	Suppressor of tumorigenicity protein 14	ST14
307	Galectin-7	LGALS7
308	Carnitine O-acetyltransferase	CRAT
309	Serum amyloid P-component	APCS
310	Phenylalanine-4-hydroxylase	PAH
311	C-1-tetrahydrofolate synthase, cytoplasmic	MTHFD1
312	C-X-C chemokine receptor type 2	CXCR2
313	C-X-C chemokine receptor type 1	CXCR1
314	Glutamate carboxypeptidase 2	FOLH1
315	Dual specificity mitogen-activated protein kinase kinase 1	MAP2K1
316	Glycogen phosphorylase, liver form	PYGL
317	Prostaglandin E synthase	PTGES
318	Transient receptor potential cation channel subfamily V member 1	TRPV1
319	Cytochrome P450 2C19	CYP2C19
320	Tyrosine-protein kinase Fyn	FYN

321	Monoglyceride lipase	MGLL
322	Sphingosine 1-phosphate receptor 4	S1PR4
323	Photoreceptor-specific nuclear receptor	NR2E3
324	Tyrosine-protein phosphatase non-receptor type 7	PTPN7
325	DNA dC->dU-editing enzyme APOBEC-3A	APOBEC3A
326	Thrombopoietin receptor	MPL
327	Ribonuclease UK114	RIDA
328	Peroxiredoxin-5, mitochondrial	PRDX5
329	Glycine amidinotransferase, mitochondrial	GATM
330	Galactosylgalactosylxylosylprotein 3-beta-glucuronosyltransferase 1	B3GAT1
331	Kinesin heavy chain	KIF5B
332	Phosphoserine phosphatase	PSPH
333	Casein kinase II subunit alpha	CSNK2A1
334	ADP-ribose pyrophosphatase, mitochondrial	NUDT9
335	NAD-dependent malic enzyme, mitochondrial	ME2
336	Intercellular adhesion molecule 2	ICAM2
337	TGF-beta receptor type-2	TGFBR2
338	U1 small nuclear ribonucleoprotein A	SNRPA
339	Methionine aminopeptidase 1	METAP1
340	ADP-ribosylation factor 1	ARF1
341	Apolipoprotein(a)	LPA
342	Heparin-binding growth factor 1	FGF1
343	Glutaredoxin-1	GLRX
344	UDP-glucose 4-epimerase	GALE
345	Pyruvate dehydrogenase E1 component subunit beta, mitochondrial	PDHB
346	Ornithine carbamoyltransferase, mitochondrial	OTC
347	L-lactate dehydrogenase B chain	LDHB
348	Carboxypeptidase B	CPB1
349	Thymidine phosphorylase	TYMP
350	Renin	REN
351	Reticulon-4 receptor	RTN4R
352	Triosephosphate isomerase	TPI1
353	Phosphoenolpyruvate carboxykinase, cytosolic [GTP]	PCK1
354	Inositol monophosphatase	IMPA1
355	Bone morphogenetic protein 7	BMP7
356	Lactotransferrin	LTF
357	Cathepsin B	CTSB
358	Basic fibroblast growth factor receptor 1	FGFR1
359	cGMP-specific 3,5-cyclic phosphodiesterase	PDE5A
360	Peroxisome proliferator-activated receptor alpha	PPARA
361	Leukotriene B4 receptor 1	LTB4R
362	Sphingosine 1-phosphate receptor 1	S1PR1
363	Cannabinoid receptor 2	CNR2
364	Prostacyclin receptor	PTGIR
365	Prostaglandin F2-alpha receptor	PTGFR
366	Sterol O-acyltransferase 1	SOAT1

367	Prostaglandin D2 receptor	PTGDR
368	Phospholipase A2, membrane associated	PLA2G2A
369	Sphingosine 1-phosphate receptor 5	S1PR5
370	Prostaglandin E2 receptor EP2 subtype	PTGER2
371	Histone deacetylase 4	HDAC4
372	Sphingosine 1-phosphate receptor 2	S1PR2
373	Fatty-acid amide hydrolase 1	FAAH
374	Thromboxane A2 receptor	TBXA2R
375	Thromboxane-A synthase	TBXAS1
376	Free fatty acid receptor 1	FFAR1
377	Galanin receptor type 3	GALR3
378	Sphingosine 1-phosphate receptor 3	S1PR3
379	Farnesyl pyrophosphate synthase	FDPS
380	NAD-dependent protein deacetylase sirtuin-2	SIRT2
381	Prostaglandin E2 receptor EP4 subtype	PTGER4
382	Histone deacetylase 3	HDAC3
383	Cannabinoid receptor 1	CNR1
384	Cathepsin G	CTSG
385	DNA dC->dU-editing enzyme APOBEC-3G	APOBEC3G
386	Bifunctional epoxide hydrolase 2	EPHX2
387	Histone deacetylase 6	HDAC6
388	Angiotensin-converting enzyme	ACE
389	Tyrosine-protein phosphatase non-receptor type 2	PTPN2
390	Prostaglandin D2 receptor 2	PTGDR2
391	Glutathione-requiring prostaglandin D synthase	HPGDS
392	Kynurenine--oxoglutarate transaminase 1	KYAT1
393	Cytochrome P450 11B2, mitochondrial	CYP11B2
394	Serine/threonine-protein kinase pim-2	PIM2
395	Perilipin-5	PLIN5
396	Cytochrome P450 11B1, mitochondrial	CYP11B1
397	Cell division cycle 7-related protein kinase	CDC7
398	Serine/threonine-protein kinase pim-3	PIM3
399	5-hydroxytryptamine receptor 3A	HTR3A
400	Capsid scaffolding protein	UL80
401	Dual specificity protein kinase CLK1	CLK1
402	Alpha-2C adrenergic receptor	ADRA2C
403	Histamine H4 receptor	HRH4
404	Voltage-dependent T-type calcium channel subunit alpha-1H	CACNA1H
405	NF-kappa-B inhibitor alpha	NFKBIA
406	Interleukin-1 beta	IL1B
407	Caspase-1	CASP1
408	Interferon gamma	IFNG
409	Pituitary adenylate cyclase-activating polypeptide	ADCYAP1
410	Proteasome assembly chaperone 1	PSMG1
411	Dual specificity mitogen-activated protein kinase kinase 4	MAP2K4
412	Corticosteroid 11-beta-dehydrogenase isozyme 2	HSD11B2

413	Sodium/glucose cotransporter 2	SLC5A2
414	Sodium/glucose cotransporter 1	SLC5A1
415	Eosinophil lysophospholipase	CLC
416	cGMP-inhibited 3,5-cyclic phosphodiesterase B	PDE3B
417	Immunoglobulin alpha Fc receptor	FCAR
418	Cathepsin L2	CTSV
419	Chitotriosidase-1	CHIT1
420	Gastrotropin	FABP6
421	Neutrophil collagenase	MMP8
422	Group 10 secretory phospholipase A2	PLA2G10
423	5-hydroxytryptamine receptor 5A	HTR5A
424	Dual specificity tyrosine-phosphorylation-regulated kinase 1A	DYRK1A
425	Proto-oncogene tyrosine-protein kinase receptor Ret	RET
426	Protein kinase C beta type	PRKCB
427	Alpha-2B adrenergic receptor	ADRA2B
428	D(1A) dopamine receptor	DRD1
429	Macrophage metalloelastase	MMP12
430	Serine/threonine-protein kinase PAK 7	PAK5
431	Superoxide dismutase [Mn], mitochondrial	SOD2
432	Fibroblast growth factor receptor 2	FGFR2
433	Sorbitol dehydrogenase	SORD
434	Cytidine deaminase	CDA
435	Calcium-activated potassium channel subunit alpha 1	KCNMA1
436	Alpha-1D adrenergic receptor	ADRA1D
437	Endothelin-1 receptor	EDNRA
438	Endothelin B receptor	EDNRB
439	Dual specificity protein kinase CLK4	CLK4
440	Pyridoxine-5-phosphate oxidase	PNPO
441	Delta-type opioid receptor	OPRD1
442	Voltage-dependent L-type calcium channel subunit alpha-1S	CACNA1S
443	Type IV phosphodiesterase	PDE4
444	5-hydroxytryptamine receptor 1A	HTR1A
445	D(2) dopamine receptor	DRD2
446	Histamine H1 receptor	HRH1
447	5-hydroxytryptamine receptor 2C	HTR2C
448	5-hydroxytryptamine receptor 1B	HTR1B
449	Plasminogen	PLG
450	Sigma non-opioid intracellular receptor 1	SIGMAR1
451	Retinol-binding protein 4	RBP4
452	Beta-lactamase	DPEP1
453	cAMP-dependent protein kinase inhibitor alpha	PKIA
454	Vascular endothelial growth factor A	VEGFA
455	Cyclin-dependent kinase inhibitor 1	CDKN1A
456	Cellular tumor antigen p53	TP53
457	Fatty acid synthase	FASN
458	Superoxide dismutase [Cu-Zn]	SOD1

459	Catalase	CAT
460	Hypoxia-inducible factor 1-alpha	HIF1A
461	Cytosolic phospholipase A2	PLA2G4A
462	Canalicular multispecific organic anion transporter 1	ABCC2
463	Serine/threonine-protein kinase mTOR	MTOR
464	Catenin alpha-1	CTNNA1
465	Non-receptor tyrosine-protein kinase TYK2	TYK2
466	Glutamate receptor 2	GRIA2
467	Estrogen receptor	CCND1
468	Proto-oncogene c-Fos	FOS
469	Fos-related antigen 1	FOSL1
470	Fos-related antigen 2	FOSL2
471	Myeloperoxidase	MPO
472	Insulin-like growth factor II	IGF2
473	Cytochrome c	CYCS
474	Nuclear factor of activated T-cells, cytoplasmic 1	NFATC1
475	Tudor domain-containing protein 7	TDRD7
476	Egl nine homolog 1	EGLN1
477	NADPH oxidase 5	NOX5
478	Fatty acid-binding protein, epidermal	FABP5
479	Apolipoprotein D	APOD
480	Lysine-specific demethylase 4D-like	KDM4E
481	G protein-coupled receptor kinase 6	GRK6
482	Inosine-5'-monophosphate dehydrogenase 2	IMPDH2
483	Carbonyl reductase [NADPH] 1	CBR1
484	Bcl-2-like protein 1	BCL2L1
485	Interleukin-10	IL10
486	Retinoblastoma-associated protein	RB1
487	Cell division protein kinase 4	CDK4
488	Interleukin-6	IL6
489	DNA topoisomerase 1	TOP1
490	Proliferating cell nuclear antigen	PCNA
491	Receptor tyrosine-protein kinase erbB-2	ERBB2
492	Baculoviral IAP repeat-containing protein 5	BIRC5
493	Interleukin-2	IL2
494	Interleukin-4	IL4
495	Baculoviral IAP repeat-containing protein 4	BIRC4
496	CD40 ligand	CD40LG
497	Kinetochore protein Nuf2	NUF2
498	Adenylate cyclase type 2	ADCY2
499	Hepatocyte growth factor receptor	MET
500	Lymphocyte differentiation antigen CD38	CD38
501	Aldo-keto reductase family 1 member B10	AKR1B10
502	Tankyrase-2	TNKS2
503	Tankyrase-1	TNKS
504	Serine/threonine-protein kinase 6	STK6

505	Casein kinase I isoform gamma-2	CSNK1G2
506	Eukaryotic translation initiation factor 6	EIF6
507	Pro-epidermal growth factor	EGF
508	Cyclin-dependent kinase inhibitor 2A, isoforms 1/2/3	CDKN2A
509	ETS domain-containing protein Elk-1	ELK1
510	NADPH--cytochrome P450 reductase	POR
511	Ornithine decarboxylase	ODC1
512	RAF proto-oncogene serine/threonine-protein kinase	RAF1
513	Protein CBFA2T1	RUNX1T1
514	Probable E3 ubiquitin-protein ligase HERC5	HERC5
515	78 kDa glucose-regulated protein	HSPA5
516	Acetyl-CoA carboxylase 1	ACACA
517	Caveolin-1	CAV1
518	Myc proto-oncogene protein	MYC
519	Tissue factor	F3
520	Gap junction alpha-1 protein	GJA1
521	C-C motif chemokine 2	CCL2
522	Prostaglandin E2 receptor EP3 subtype	PTGER3
523	Interleukin-8	CXCL8
524	Dual oxidase 2	DUOX2
525	Heat shock protein beta-1	HSPB1
526	Estrogen sulfotransferase	SULT1E1
527	Maltase-glucoamylase, intestinal	MGAM
528	Tissue-type plasminogen activator	PLAT
529	Thrombomodulin	THBD
530	Plasminogen activator inhibitor 1	SERPINE1
531	Collagen alpha-1(I) chain	COL1A1
532	Phosphatidylinositol-3,4,5-trisphosphate 3-phosphatase and dual-speci	PTEN
533	Interleukin-1 alpha	IL1A
534	Neutrophil cytosol factor 1	NCF1
535	Nuclear factor erythroid 2-related factor 2	NFE2L2
536	Collagen alpha-1(III) chain	COL8A1
537	DNA gyrase subunit B	gyrB
538	C-X-C motif chemokine 11	CXCL11
539	C-X-C motif chemokine 2	CXCL2
540	DDB1- and CUL4-associated factor 5	DCAF5
541	Serine/threonine-protein kinase Chk2	CHEK2
542	Claudin-4	CLDN4
543	Heat shock factor protein 1	HSF1
544	C-reactive protein	CRP
545	C-X-C motif chemokine 10	CXCL10
546	Inhibitor of nuclear factor kappa-B kinase subunit alpha	CHUK
547	Osteopontin	SPP1
548	Runt-related transcription factor 2	RUNX2
549	Ras association domain-containing protein 1	RASSF1
550	Transcription factor E2F1	E2F1

551	Transcription factor E2F2	E2F2
552	Prostatic acid phosphatase	ACP3
553	Insulin-like growth factor-binding protein 3	IGFBP3
554	Interferon regulatory factor 1	IRF1
555	Receptor tyrosine-protein kinase erbB-3	ERBB3
556	Procollagen C-endopeptidase enhancer 1	PCOLCE
557	Puromycin-sensitive aminopeptidase	NPEPPS
558	Hexokinase-2	HK2
559	Homeobox protein Nkx-3.1	NKX3-1
560	Ras GTPase-activating protein 1	RASA1
561	Vasopressin V2 receptor	AVPR2
562	Serine/threonine-protein kinase Aurora-B	AURKB
563	Dopamine D4 receptor	DRD4
564	PI3-kinase p85-alpha subunit	PIK3R1
565	Focal adhesion kinase 1	PTK2
566	Carbonic anhydrase III	CA3
567	Protein kinase N1	PKN1
568	Serine/threonine-protein kinase NEK2	NEK2
569	CaM kinase II beta	CAMK2B
570	ALK tyrosine kinase receptor	ALK
571	Serine/threonine-protein kinase NEK6	NEK6
572	Tyrosine-protein kinase receptor UFO	AXL
573	NUAK family SNF1-like kinase 1	NUAK1
574	Aldo-keto reductase family 1 member C4 (by homology)	AKR1C4
575	Aldehyde reductase (by homology)	AKR1A1
576	Microtubule-associated protein tau	MAPT
577	Myosin light chain kinase, smooth muscle	MYLK
578	DNA-(apurinic or apyrimidinic site) lyase	APEX1
579	Platelet-activating factor receptor	PTAFR
580	Histone deacetylase 2	HDAC2
