# Supplementary material for: Integrating Network Pharmacology and Experimental Validation to Elucidate the Mechanism of Yiqi Yangyin Decoction in Suppressing Non-Small-Cell Lung Cancer
Source: Biomed Res Int. 2023 Feb 20;2023:4967544. doi: 10.1155/2023/4967544 (PMC9980286; doi:10.1155/2023/4967544)
Supplement: Supplementary 3 — Supplementary Table 2: targets associated with NSCLC. [file 4967544.f3.pdf]

Supplementay Table 2: NSCLC-related targets

Number	Protein name	Gene name
1	Tumor Protein P53	TP53
2	Epidermal Growth Factor Receptor	EGFR
3	BRCA1 DNA Repair Associated	BRCA1
4	BRCA2 DNA Repair Associated	BRCA2
5	KRAS Proto-Oncogene, GTPase	KRAS
6	Phosphatase And Tensin Homolog	PTEN
7	MET Proto-Oncogene, Receptor Tyrosine Kinase	MET
8	Phosphatidylinositol-4,5-Bisphosphate 3-Kinase Catalytic Subunit Al	PIK3CA
9	Erb-B2 Receptor Tyrosine Kinase 2	ERBB2
10	ATM Serine/Threonine Kinase	ATM
11	B-Raf Proto-Oncogene, Serine/Threonine Kinase	BRAF
12	AKT Serine/Threonine Kinase 1	AKT1
13	Cyclin Dependent Kinase Inhibitor 2A	CDKN2A
14	Cadherin 1	CDH1
15	ALK Receptor Tyrosine Kinase	ALK
16	Checkpoint Kinase 2	CHEK2
17	APC Regulator Of WNT Signaling Pathway	APC
18	MutL Homolog 1	MLH1
19	Caspase 8	CASP8
20	NRAS Proto-Oncogene, GTPase	NRAS
21	MutS Homolog 2	MSH2
22	Catenin Beta 1	CTNNB1
23	RB Transcriptional Corepressor 1	RB1
24	MutS Homolog 6	MSH6
25	Serine/Threonine Kinase 11	STK11
26	Cyclin D1	CCND1
27	Nibrin	NBN
28	Fas Ligand	FASLG
29	Mitogen-Activated Protein Kinase Kinase 1	MAP2K1
30	HRas Proto-Oncogene, GTPase	HRAS
31	KIT Proto-Oncogene, Receptor Tyrosine Kinase	KIT
32	Telomerase Reverse Transcriptase	TERT
33	PMS1 Homolog 2, Mismatch Repair System Component	PMS2
34	Fibroblast Growth Factor Receptor 3	FGFR3
35	SMAD Family Member 4	SMAD4
36	Ret Proto-Oncogene	RET
37	ROS Proto-Oncogene 1, Receptor Tyrosine Kinase	ROS1
38	MYC Proto-Oncogene, BHLH Transcription Factor	MYC
39	Signal Transducer And Activator Of Transcription 3	STAT3
40	BCL2 Apoptosis Regulator	BCL2
41	Chromosome 11 Open Reading Frame 65	C11orf65
42	Interleukin 6	IL6
43	Interferon Regulatory Factor 1	IRF1
44	Transforming Growth Factor Beta 1	TGFB1

45	Estrogen Receptor 1	ESR1
46	DNA Polymerase Epsilon, Catalytic Subunit	POLE
47	Parkin RBR E3 Ubiquitin Protein Ligase	PRKN
48	Protein Tyrosine Phosphatase Receptor Type C	PTPRC
49	Tumor Necrosis Factor	TNF
50	Vascular Endothelial Growth Factor A	VEGFA
51	Fibroblast Growth Factor Receptor 2	FGFR2
52	BRCA1 Associated RING Domain 1	BARD1
53	BCL2 Associated X, Apoptosis Regulator	BAX
54	Dicer 1, Ribonuclease III	DICER1
55	Mechanistic Target Of Rapamycin Kinase	MTOR
56	ERCC Excision Repair 6, Chromatin Remodeling Factor	ERCC6
57	Epithelial Cell Adhesion Molecule	EPCAM
58	MutY DNA Glycosylase	MUTYH
59	Solute Carrier Family 22 Member 18	SLC22A18
60	Transforming Growth Factor Beta Receptor 2	TGFBR2
61	Cyclin Dependent Kinase Inhibitor 1B	CDKN1B
62	NK2 Homeobox 1	NKX2-1
63	Protein Phosphatase 2 Scaffold Subunit Abeta	PPP2R1B
64	Neurofibromin 1	NF1
65	Cyclin Dependent Kinase 4	CDK4
66	Patched 1	PTCH1
67	Adenosine Deaminase	ADA
68	RAD50 Double Strand Break Repair Protein	RAD50
69	Interleukin 1 Beta	IL1B
70	MDM2 Proto-Oncogene	MDM2
71	SWI/SNF Related, Matrix Associated, Actin Dependent Regulator Of	SMARCA4
72	Fas Cell Surface Death Receptor	FAS
73	RAD51 Paralog C	RAD51C
74	Cyclin Dependent Kinase Inhibitor 1A	CDKN1A
75	CD274 Molecule	CD274
76	TSC Complex Subunit 2	TSC2
77	Cytochrome P450 Family 2 Subfamily A Member 6	CYP2A6
78	SRC Proto-Oncogene, Non-Receptor Tyrosine Kinase	SRC
79	Von Hippel-Lindau Tumor Suppressor	VHL
80	Peroxisome Proliferator Activated Receptor Gamma	PPARG
81	Mitogen-Activated Protein Kinase 1	MAPK1
82	TSC Complex Subunit 1	TSC1
83	Androgen Receptor	AR
84	Phosphoinositide-3-Kinase Regulatory Subunit 1	PIK3R1
85	Interferon Gamma	IFNG
86	Mitogen-Activated Protein Kinase Kinase Kinase 8	MAP3K8
87	Interleukin 10	IL10
88	Leucine Rich Repeat Containing 56	LRRC56
89	Matrix Remodeling Associated 5	MXRA5
90	C-X-C Motif Chemokine Ligand 8	CXCL8

91	Mucin 1, Cell Surface Associated	MUC1
92	Caspase 3	CASP3
93	RAD51 Recombinase	RAD51
94	Axin 2	AXIN2
95	Notch Receptor 1	NOTCH1
96	C-X-C Motif Chemokine Receptor 4	CXCR4
97	MRE11 Homolog, Double Strand Break Repair Nuclease	MRE11
98	Epidermal Growth Factor	EGF
99	E1A Binding Protein P300	EP300
100	Tumor Protein P73	TP73
101	Fibroblast Growth Factor Receptor 1	FGFR1
102	Cytotoxic T-Lymphocyte Associated Protein 4	CTLA4
103	Interleukin 7 Receptor	IL7R
104	Hypoxia Inducible Factor 1 Subunit Alpha	HIF1A
105	ERCC Excision Repair 2, TFIIH Core Complex Helicase Subunit	ERCC2
106	BCL10 Immune Signaling Adaptor	BCL10
107	Protein Tyrosine Phosphatase Non-Receptor Type 11	PTPN11
108	Kinase Insert Domain Receptor	KDR
109	Matrix Metallopeptidase 9	MMP9
110	F-Box And WD Repeat Domain Containing 7	FBXW7
111	Janus Kinase 2	JAK2
112	BRCA1 Associated Protein 1	BAP1
113	Matrix Metallopeptidase 2	MMP2
114	Prostaglandin-Endoperoxide Synthase 2	PTGS2
115	ATP Binding Cassette Subfamily B Member 1	ABCB1
116	Fragile Histidine Triad Diadenosine Triphosphatase	FHIT
117	Nuclear Factor Kappa B Subunit 1	NFKB1
118	Insulin Like Growth Factor 2	IGF2
119	CD44 Molecule (Indian Blood Group)	CD44
120	Programmed Cell Death 1	PDCD1
121	TNF Receptor Superfamily Member 10b	TNFRSF10B
122	SET Domain Containing 2, Histone Lysine Methyltransferase	SETD2
123	Sarcolemma Associated Protein	SLMAP
124	PiggyBac Transposable Element Derived 3	PGBD3
125	Recombination Activating 2	RAG2
126	ERCC Excision Repair 1, Endonuclease Non-Catalytic Subunit	ERCC1
127	Menin 1	MEN1
128	Interleukin 2	IL2
129	Catenin Alpha 1	CTNNA1
130	Baculoviral IAP Repeat Containing 5	BIRC5
131	Signal Transducer And Activator Of Transcription 1	STAT1
132	MutS Homolog 3	MSH3
133	Jun Proto-Oncogene, AP-1 Transcription Factor Subunit	JUN
134	X-Ray Repair Cross Complementing 3	XRCC3
135	NFE2 Like BZIP Transcription Factor 2	NFE2L2
136	Aurora Kinase A	AURKA

137	Integrin Subunit Beta 1	ITGB1
138	Kruppel Like Factor 6	KLF6
139	RUNX Family Transcription Factor 1	RUNX1
140	Platelet Derived Growth Factor Receptor Alpha	PDGFRA
141	Insulin Like Growth Factor 1 Receptor	IGF1R
142	Raf-1 Proto-Oncogene, Serine/Threonine Kinase	RAF1
143	Janus Kinase 3	JAK3
144	Tumor Protein P63	TP63
145	Fibroblast Growth Factor 2	FGF2
146	BCL2 Like 1	BCL2L1
147	CEA Cell Adhesion Molecule 5	CEACAM5
148	Bone Morphogenetic Protein Receptor Type 1A	BMPR1A
149	Major Histocompatibility Complex, Class II, DR Beta 1	HLA-DRB1
150	Interleukin 4	IL4
151	X-Ray Repair Cross Complementing 1	XRCC1
152	BCL6 Transcription Repressor	BCL6
153	Caveolin 1	CAV1
154	Colony Stimulating Factor 3	CSF3
155	SRY-Box Transcription Factor 9	SOX9
156	8-Oxoguanine DNA Glycosylase	OGG1
157	Proliferating Cell Nuclear Antigen	PCNA
158	NFKB Inhibitor Alpha	NFKBIA
159	Hepatocyte Growth Factor	HGF
160	MutL Homolog 3	MLH3
161	Surfactant Protein A1	SFTPA1
162	Cytochrome P450 Family 1 Subfamily A Member 1	CYP1A1
163	ABL Proto-Oncogene 1, Non-Receptor Tyrosine Kinase	ABL1
164	Mitogen-Activated Protein Kinase 8	MAPK8
165	Discoidin Domain Receptor Tyrosine Kinase 2	DDR2
166	KIT Ligand	KITLG
167	C-X-C Motif Chemokine Ligand 12	CXCL12
168	Cyclin Dependent Kinase 2	CDK2
169	Glutathione S-Transferase Pi 1	GSTP1
170	Insulin Like Growth Factor 1	IGF1
171	X-Linked Inhibitor Of Apoptosis	XIAP
172	Intercellular Adhesion Molecule 1	ICAM1
173	Secreted Phosphoprotein 1	SPP1
174	RELA Proto-Oncogene, NF-KB Subunit	RELA
175	Platelet Derived Growth Factor Receptor Beta	PDGFRB
176	Neural Cell Adhesion Molecule 1	NCAM1
177	SWI/SNF Related, Matrix Associated, Actin Dependent Regulator Of	SMARCB1
178	Erb-B2 Receptor Tyrosine Kinase 3	ERBB3
179	Ras Homolog Family Member A	RHOA
180	Enhancer Of Zeste 2 Polycomb Repressive Complex 2 Subunit	EZH2
181	SMAD Family Member 3	SMAD3
182	WT1 Transcription Factor	WT1

183	Isocitrate Dehydrogenase (NADP(+)) 1	IDH1
184	X-Ray Repair Cross Complementing 2	XRCC2
185	Fibronectin 1	FN1
186	MCL1 Apoptosis Regulator, BCL2 Family Member	MCL1
187	CD4 Molecule	CD4
188	Poly(ADP-Ribose) Polymerase 1	PARP1
189	Colony Stimulating Factor 2	CSF2
190	Glutathione S-Transferase Mu 1	GSTM1
191	Protein Tyrosine Kinase 2	PTK2
192	O-6-Methylguanine-DNA Methyltransferase	MGMT
193	Caspase 9	CASP9
194	Forkhead Box P3	FOXP3
195	Interleukin 2 Receptor Subunit Alpha	IL2RA
196	Homeobox B13	HOXB13
197	Enolase 2	ENO2
198	Toll Like Receptor 2	TLR2
199	Estrogen Receptor 2	ESR2
200	Keratin 19	KRT19
201	Cyclin Dependent Kinase 6	CDK6
202	Cyclin Dependent Kinase 1	CDK1
203	Matrix Metallopeptidase 1	MMP1
204	Retinoic Acid Receptor Beta	RARB
205	Inhibitor Of Growth Family Member 1	ING1
206	TNF Superfamily Member 10	TNFSF10
207	Cyclin Dependent Kinase Inhibitor 2B	CDKN2B
208	Surfactant Protein C	SFTPC
209	Transforming Growth Factor Beta Receptor 1	TGFBR1
210	Rac Family Small GTPase 1	RAC1
211	Phosphatidylinositol-4,5-Bisphosphate 3-Kinase Catalytic Subunit Ga	PIK3CG
212	Marker Of Proliferation Ki-67	MKI67
213	Fms Related Receptor Tyrosine Kinase 1	FLT1
214	Mitogen-Activated Protein Kinase 14	MAPK14
215	SRY-Box Transcription Factor 2	SOX2
216	Mitogen-Activated Protein Kinase 3	MAPK3
217	ATP Binding Cassette Subfamily G Member 2 (Junior Blood Group)	ABCG2
218	AKT Serine/Threonine Kinase 2	AKT2
219	Ras Association Domain Family Member 1	RASSF1
220	Thymidylate Synthetase	TYMS
221	BUB1 Mitotic Checkpoint Serine/Threonine Kinase B	BUB1B
222	C-C Motif Chemokine Ligand 2	CCL2
223	Transforming Growth Factor Alpha	TGFA
224	Plasminogen Activator, Urokinase	PLAU
225	GATA Binding Protein 2	GATA2
226	ATP Binding Cassette Subfamily C Member 1	ABCC1
227	DNA Methyltransferase 1	DNMT1
228	MYCN Proto-Oncogene, BHLH Transcription Factor	MYCN

229	Nucleophosmin 1	NPM1
230	CF Transmembrane Conductance Regulator	CFTR
231	CD40 Molecule	CD40
232	E2F Transcription Factor 1	E2F1
233	POU Class 5 Homeobox 1	POU5F1
234	Fms Related Receptor Tyrosine Kinase 3	FLT3
235	Endoglin	ENG
236	Keratin 7	KRT7
237	Cell Adhesion Molecule 1	CADM1
238	Toll Like Receptor 4	TLR4
239	Prominin 1	PROM1
240	Erb-B2 Receptor Tyrosine Kinase 4	ERBB4
241	Cytochrome C, Somatic	CYCS
242	Solute Carrier Family 2 Member 1	SLC2A1
243	Integrin Subunit Alpha 3	ITGA3
244	DNA Methyltransferase 3 Alpha	DNMT3A
245	Platelet And Endothelial Cell Adhesion Molecule 1	PECAM1
246	Caspase 10	CASP10
247	Melanocyte Inducing Transcription Factor	MITF
248	EPH Receptor B2	EPHB2
249	Cell Division Cycle 42	CDC42
250	Hepatitis A Virus Cellular Receptor 2	HAVCR2
251	AT-Rich Interaction Domain 1A	ARID1A
252	Sp1 Transcription Factor	SP1
253	Yes1 Associated Transcriptional Regulator	YAP1
254	Vimentin	VIM
255	Heat Shock Protein 90 Alpha Family Class A Member 1	HSP90AA1
256	DLEC1 Cilia And Flagella Associated Protein	DLEC1
257	Protein Kinase C Alpha	PRKCA
258	Kelch Like ECH Associated Protein 1	KEAP1
259	CAMP Responsive Element Binding Protein 1	CREB1
260	Chromogranin A	CHGA
261	Cyclin B1	CCNB1
262	DNA Topoisomerase II Alpha	TOP2A
263	Major Histocompatibility Complex, Class I, B	HLA-B
264	Cyclin A2	CCNA2
265	Beta-2-Microglobulin	B2M
266	Interleukin 3	IL3
267	Zeta Chain Of T Cell Receptor Associated Protein Kinase 70	ZAP70
268	Vascular Endothelial Growth Factor C	VEGFC
269	Fms Related Receptor Tyrosine Kinase 4	FLT4
270	Major Histocompatibility Complex, Class I, G	HLA-G
271	Retinoic Acid Receptor Alpha	RARA
272	Platelet Derived Growth Factor Subunit B	PDGFB
273	Hemoglobin Subunit Beta	HBB
274	Serpin Family A Member 1	SERPINA1

275	Heme Oxygenase 1	HMOX1
276	Zinc Finger E-Box Binding Homeobox 1	ZEB1
277	Cadherin 2	CDH2
278	Insulin Like Growth Factor Binding Protein 3	IGFBP3
279	DNA Polymerase Kappa	POLK
280	Macrophage Scavenger Receptor 1	MSR1
281	NAD(P)H Quinone Dehydrogenase 1	NQO1
282	WW Domain Containing Oxidoreductase	WWOX
283	GNAS Complex Locus	GNAS
284	Protein Kinase C Delta	PRKCD
285	Surfactant Protein A2	SFTPA2
286	NF2, Moesin-Ezrin-Radixin Like (MERLIN) Tumor Suppressor	NF2
287	Albumin	ALB
288	Keratin 18	KRT18
289	Mucin 5B, Oligomeric Mucus/Gel-Forming	MUC5B
290	C-C Motif Chemokine Receptor 6	CCR6
291	XPA, DNA Damage Recognition And Repair Factor	XPA
292	DNA Topoisomerase I	TOP1
293	Interleukin 1 Receptor Antagonist	IL1RN
294	Baculoviral IAP Repeat Containing 3	BIRC3
295	Checkpoint Kinase 1	CHEK1
296	ERCC Excision Repair 4, Endonuclease Catalytic Subunit	ERCC4
297	CD28 Molecule	CD28
298	Matrix Metallopeptidase 14	MMP14
299	SMAD Family Member 2	SMAD2
300	High Mobility Group Box 1	HMGB1
301	Fos Proto-Oncogene, AP-1 Transcription Factor Subunit	FOS
302	Isocitrate Dehydrogenase (NADP(+)) 2	IDH2
303	GLI Family Zinc Finger 1	GLI1
304	Plasminogen Activator, Urokinase Receptor	PLAUR
305	Gap Junction Protein Alpha 1	GJA1
306	DNA Methyltransferase 3 Beta	DNMT3B
307	Mitotic Arrest Deficient 1 Like 1	MAD1L1
308	Synaptophysin	SYP
309	C-C Motif Chemokine Ligand 5	CCL5
310	Cyclin Dependent Kinase Inhibitor 3	CDKN3
311	Twist Family BHLH Transcription Factor 1	TWIST1
312	TIMP Metallopeptidase Inhibitor 1	TIMP1
313	Nitric Oxide Synthase 2	NOS2
314	Spleen Associated Tyrosine Kinase	SYK
315	Polo Like Kinase 1	PLK1
316	Cytochrome P450 Family 1 Subfamily B Member 1	CYP1B1
317	Progesterone Receptor	PGR
318	Superoxide Dismutase 2	SOD2
319	Thymidine Phosphorylase	TYMP
320	Interleukin 7	IL7

321	Thrombospondin 1	THBS1
322	NME/NM23 Nucleoside Diphosphate Kinase 1	NME1
323	CD34 Molecule	CD34
324	CASP8 And FADD Like Apoptosis Regulator	CFLAR
325	Mucin 16, Cell Surface Associated	MUC16
326	DLC1 Rho GTPase Activating Protein	DLC1
327	Sonic Hedgehog Signaling Molecule	SHH
328	Macrophage Migration Inhibitory Factor	MIF
329	CREB Binding Protein	CREBBP
330	Inhibitor Of Nuclear Factor Kappa B Kinase Subunit Beta	IKBKB
331	Desmoplakin	DSP
332	Matrix Metallopeptidase 7	MMP7
333	Phosphatidylinositol-4,5-Bisphosphate 3-Kinase Catalytic Subunit De	PIK3CD
334	BUB1 Mitotic Checkpoint Serine/Threonine Kinase	BUB1
335	Protein Phosphatase, Mg2+/Mn2+ Dependent 1D	PPM1D
336	Insulin	INS
337	EPH Receptor A2	EPHA2
338	Methylenetetrahydrofolate Reductase	MTHFR
339	Snail Family Transcriptional Repressor 1	SNAI1
340	MYC Associated Factor X	MAX
341	Heat Shock Protein Family B (Small) Member 1	HSPB1
342	Major Histocompatibility Complex, Class I, A	HLA-A
343	Gastrin Releasing Peptide	GRP
344	Interleukin 17A	IL17A
345	ATR Serine/Threonine Kinase	ATR
346	Prohibitin 1	PHB1
347	Cyclin D3	CCND3
348	Snail Family Transcriptional Repressor 2	SNAI2
349	PMS1 Homolog 1, Mismatch Repair System Component	PMS1
350	Protection Of Telomeres 1	POT1
351	ETS Variant Transcription Factor 6	ETV6
352	BCL2 Associated Agonist Of Cell Death	BAD
353	Zinc Finger Homeobox 3	ZFHX3
354	Smoothened, Frizzled Class Receptor	SMO
355	Thy-1 Cell Surface Antigen	THY1
356	Histone Deacetylase 1	HDAC1
357	Neuregulin 1	NRG1
358	MYB Proto-Oncogene, Transcription Factor	MYB
359	Protein Kinase D1	PRKD1
360	BCR Activator Of RhoGEF And GTPase	BCR
361	Receptor Interacting Serine/Threonine Kinase 1	RIPK1
362	Actin Alpha 2, Smooth Muscle	ACTA2
363	BCL2 Antagonist/Killer 1	BAK1
364	Interleukin 13	IL13
365	FA Complementation Group D2	FANCD2
366	Damage Specific DNA Binding Protein 2	DDB2

367	Bone Morphogenetic Protein 6	BMP6
368	Ribosomal Protein S6 Kinase B1	RPS6KB1
369	Cbl Proto-Oncogene	CBL
370	BCL2 Like 11	BCL2L11
371	Basigin (Ok Blood Group)	BSG
372	Paired Box 5	PAX5
373	CD8a Molecule	CD8A
374	SOS Ras/Rac Guanine Nucleotide Exchange Factor 1	SOS1
375	Abraxas 1, BRCA1 A Complex Subunit	ABRAXAS1
376	Transferrin Receptor	TFRC
377	Heat Shock Protein Family A (Hsp70) Member 5	HSPA5
378	Cell Division Cycle 25A	CDC25A
379	Annexin A5	ANXA5
380	Amphiregulin	AREG
381	Keratin 8	KRT8
382	Protein Tyrosine Kinase 2 Beta	PTK2B
383	Bone Morphogenetic Protein 2	BMP2
384	AKT Serine/Threonine Kinase 3	AKT3
385	Neuropilin 1	NRP1
386	Cancer/Testis Antigen 1B	CTAG1B
387	Epithelial Cell Transforming 2	ECT2
388	Serpin Family E Member 1	SERPINE1
389	SMAD Family Member 7	SMAD7
390	Homeostatic Iron Regulator	HFE
391	Parathyroid Hormone Like Hormone	PTHLH
392	C-C Motif Chemokine Receptor 7	CCR7
393	Keratin 5	KRT5
394	Cyclin E1	CCNE1
395	CD36 Molecule	CD36
396	Integrin Subunit Alpha 6	ITGA6
397	S-Phase Kinase Associated Protein 2	SKP2
398	Dihydrofolate Reductase	DHFR
399	CEA Cell Adhesion Molecule 3	CEACAM3
400	Surfactant Protein B	SFTPB
401	Interleukin 1 Alpha	IL1A
402	Catenin Delta 1	CTNND1
403	U2 Small Nuclear RNA Auxiliary Factor 1	U2AF1
404	Histone Deacetylase 9	HDAC9
405	Forkhead Box O3	FOXO3
406	Interleukin 2 Receptor Subunit Gamma	IL2RG
407	AXL Receptor Tyrosine Kinase	AXL
408	TIMP Metallopeptidase Inhibitor 2	TIMP2
409	Insulin Receptor Substrate 1	IRS1
410	Neurotrophic Receptor Tyrosine Kinase 1	NTRK1
411	Podoplanin	PDPN
412	Minichromosome Maintenance Complex Component 4	MCM4

413	Cell Division Cycle 25C	CDC25C
414	Integrin Subunit Alpha V	ITGAV
415	Transforming Growth Factor Beta 2	TGFB2
416	WD Repeat Containing Antisense To TP53	WRAP53
417	Polybromo 1	PBRM1
418	Forkhead Box M1	FOXM1
419	Nitric Oxide Synthase 3	NOS3
420	Apurinic/Apyrimidinic Endodeoxyribonuclease 1	APEX1
421	Hyaluronan Mediated Motility Receptor	HMMR
422	Leptin	LEP
423	Component Of Inhibitor Of Nuclear Factor Kappa B Kinase Complex	CHUK
424	SHC Adaptor Protein 1	SHC1
425	CD40 Ligand	CD40LG
426	Endothelin 1	EDN1
427	Carbonic Anhydrase 9	CA9
428	Keratin 20	KRT20
429	Cyclin Dependent Kinase Inhibitor 1C	CDKN1C
430	Secreted Protein Acidic And Cysteine Rich	SPARC
431	Fibroblast Growth Factor Receptor 4	FGFR4
432	Major Vault Protein	MVP
433	Myeloperoxidase	MPO
434	Lysyl Oxidase	LOX
435	TNF Receptor Superfamily Member 10a	TNFRSF10A
436	RAS P21 Protein Activator 1	RASA1
437	Cathepsin B	CTSB
438	Serpin Family A Member 3	SERPINA3
439	Major Histocompatibility Complex, Class II, DQ Beta 1	HLA-DQB1
440	EMAP Like 4	EML4
441	C-Reactive Protein	CRP
442	Death Associated Protein Kinase 1	DAPK1
443	Phospholipase A2 Group IIA	PLA2G2A
444	Integrin Subunit Beta 3	ITGB3
445	Nuclear Factor Kappa B Subunit 2	NFKB2
446	ATP Binding Cassette Subfamily C Member 2	ABCC2
447	Heat Shock Protein Family A (Hsp70) Member 4	HSPA4
448	Angiopoietin 2	ANGPT2
449	BH3 Interacting Domain Death Agonist	BID
450	Gremlin 1, DAN Family BMP Antagonist	GREM1
451	Cellular Communication Network Factor 2	CCN2
452	Paxillin	PXN
453	Glypican 3	GPC3
454	Forkhead Box O1	FOXO1
455	Membrane Metalloendopeptidase	MME
456	Signal Transducer And Activator Of Transcription 5B	STAT5B
457	Protein Kinase C Iota	PRKCI
458	Laminin Subunit Gamma 2	LAMC2

459	CD24 Molecule	CD24
460	Colony Stimulating Factor 1	CSF1
461	Mucin 4, Cell Surface Associated	MUC4
462	DNA Ligase 4	LIG4
463	Interleukin 4 Receptor	IL4R
464	Interferon Alpha 1	IFNA1
465	Matrix Metallopeptidase 3	MMP3
466	Dihydropyrimidine Dehydrogenase	DPYD
467	Cyclin D2	CCND2
468	Protein Kinase CAMP-Dependent Type I Regulatory Subunit Alpha	PRKAR1A
469	Fibroblast Growth Factor 7	FGF7
470	Vitamin D Receptor	VDR
471	Programmed Cell Death 4	PDCD4
472	YY1 Transcription Factor	YY1
473	A-Raf Proto-Oncogene, Serine/Threonine Kinase	ARAF
474	Calreticulin	CALR
475	RUNX Family Transcription Factor 3	RUNX3
476	Integrin Subunit Alpha 5	ITGA5
477	Junction Plakoglobin	JUP
478	Cytochrome P450 Family 19 Subfamily A Member 1	CYP19A1
479	Phosphatidylinositol-4,5-Bisphosphate 3-Kinase Catalytic Subunit Bet	PIK3CB
480	Bone Morphogenetic Protein 4	BMP4
481	Inducible T Cell Costimulator Ligand	ICOSLG
482	Notch Receptor 3	NOTCH3
483	Surfactant Protein D	SFTPD
484	Taurine Up-Regulated 1	TUG1
485	RB1 Inducible Coiled-Coil 1	RB1CC1
486	SWI/SNF Related, Matrix Associated, Actin Dependent Regulator Of	SMARCE1
487	Phospholipase C Gamma 1	PLCG1
488	TIMP Metallopeptidase Inhibitor 3	TIMP3
489	High Mobility Group AT-Hook 2	HMGA2
490	MCC Regulator Of WNT Signaling Pathway	MCC
491	Killer Cell Lectin Like Receptor K1	KLRK1
492	Interleukin 15	IL15
493	Thyroid Hormone Receptor Interactor 13	TRIP13
494	Interferon Alpha Inducible Protein 27	IFI27
495	Periostin	POSTN
496	Claudin 7	CLDN7
497	Fascin Actin-Bundling Protein 1	FSCN1
498	Transcription Factor 7 Like 2	TCF7L2
499	CEA Cell Adhesion Molecule 6	CEACAM6
500	XPC Complex Subunit, DNA Damage Recognition And Repair Facto	XPC
501	Collagen Type I Alpha 1 Chain	COL1A1
502	Enolase 1	ENO1
503	Cyclin Dependent Kinase Inhibitor 2C	CDKN2C
504	MYD88 Innate Immune Signal Transduction Adaptor	MYD88

505	Drosha Ribonuclease III	DROSHA
506	Early Growth Response 1	EGR1
507	Glycogen Synthase Kinase 3 Beta	GSK3B
508	X-Ray Repair Cross Complementing 5	XRCC5
509	Insulin Like Growth Factor 2 Receptor	IGF2R
510	Cytochrome P450 Family 3 Subfamily A Member 4	CYP3A4
511	Aldehyde Dehydrogenase 1 Family Member A1	ALDH1A1
512	C-X-C Motif Chemokine Receptor 2	CXCR2
513	LCK Proto-Oncogene, Src Family Tyrosine Kinase	LCK
514	Glyceraldehyde-3-Phosphate Dehydrogenase	GAPDH
515	S100 Calcium Binding Protein A4	S100A4
516	Axin 1	AXIN1
517	Growth Arrest And DNA Damage Inducible Alpha	GADD45A
518	Perforin 1	PRF1
519	Erythropoietin	EPO
520	Interleukin 6 Receptor	IL6R
521	Cadherin 13	CDH13
522	Aurora Kinase B	AURKB
523	N-Acetyltransferase 2	NAT2
524	Superoxide Dismutase 1	SOD1
525	Inhibitor Of DNA Binding 1, HLH Protein	ID1
526	Achaete-Scute Family BHLH Transcription Factor 1	ASCL1
527	Mitogen-Activated Protein Kinase Kinase 2	MAP2K2
528	TNF Receptor Superfamily Member 1B	TNFRSF1B
529	Ribonucleotide Reductase Catalytic Subunit M1	RRM1
530	Cytochrome P450 Family 2 Subfamily D Member 6	CYP2D6
531	Tyrosine 3-Monooxygenase/Tryptophan 5-Monooxygenase Activatior	YWHAE
532	Galectin 3	LGALS3
533	Platelet Derived Growth Factor Receptor Like	PDGFRL
534	Inducible T Cell Costimulator	ICOS
535	BMI1 Proto-Oncogene, Polycomb Ring Finger	BMI1
536	Colony Stimulating Factor 1 Receptor	CSF1R
537	Toll Like Receptor 9	TLR9
538	Baculoviral IAP Repeat Containing 2	BIRC2
539	Beclin 1	BECN1
540	Elastase, Neutrophil Expressed	ELANE
541	Notch Receptor 2	NOTCH2
542	Caspase 7	CASP7
543	Cytochrome P450 Family 2 Subfamily E Member 1	CYP2E1
544	Integrin Subunit Alpha 2	ITGA2
545	Somatostatin	SST
546	RB Binding Protein 8, Endonuclease	RBBP8
547	Integrin Linked Kinase	ILK
548	Transketolase	TKT
549	Fatty Acid Synthase	FASN
550	Zinc Finger E-Box Binding Homeobox 2	ZEB2

551	X-Ray Repair Cross Complementing 6	XRCC6
552	GATA Binding Protein 3	GATA3
553	Angiotensin I Converting Enzyme	ACE
554	Angiopoietin 1	ANGPT1
555	Ezrin	EZR
556	Thioredoxin	TXN
557	Galectin 1	LGALS1
558	TNF Receptor Superfamily Member 1A	TNFRSF1A
559	Vascular Endothelial Growth Factor D	VEGFD
560	Lamin A/C	LMNA
561	E2F Transcription Factor 3	E2F3
562	Nucleotide Binding Oligomerization Domain Containing 2	NOD2
563	Eukaryotic Translation Initiation Factor 4E	EIF4E
564	Tubulin Beta Class I	TUBB
565	Y-Box Binding Protein 1	YBX1
566	Clusterin	CLU
567	IL2 Inducible T Cell Kinase	ITK
568	ETS Proto-Oncogene 1, Transcription Factor	ETS1
569	Glutathione S-Transferase Theta 1	GSTT1
570	TNF Superfamily Member 11	TNFSF11
571	MPL Proto-Oncogene, Thrombopoietin Receptor	MPL
572	CD247 Molecule	CD247
573	Kruppel Like Factor 4	KLF4
574	RUNX Family Transcription Factor 2	RUNX2
575	Nuclear Receptor Binding SET Domain Protein 1	NSD1
576	Lysine Methyltransferase 2D	KMT2D
577	Retinoid X Receptor Alpha	RXRA
578	Alanyl Aminopeptidase, Membrane	ANPEP
579	Aldolase, Fructose-Bisphosphate A	ALDOA
580	Insulin Receptor	INSR
581	Mitochondrially Encoded Cytochrome C Oxidase I	MT-CO1
582	Serpin Family B Member 5	SERPINB5
583	Alpha Fetoprotein	AFP
584	Kallikrein Related Peptidase 3	KLK3
585	Interleukin 5	IL5
586	TNF Receptor Associated Factor 6	TRAF6
587	TEK Receptor Tyrosine Kinase	TEK
588	Secretoglobin Family 1A Member 1	SCGB1A1
589	Cholinergic Receptor Nicotinic Alpha 3 Subunit	CHRNA3
590	Cholinergic Receptor Nicotinic Alpha 5 Subunit	CHRNA5
591	Glial Cell Derived Neurotrophic Factor	GDNF
592	Keratin 14	KRT14
593	H2A Clustered Histone 18	H2AC18
594	Janus Kinase 1	JAK1
595	CD9 Molecule	CD9
596	Sirtuin 1	SIRT1

597	Endothelial PAS Domain Protein 1	EPAS1
598	Cadherin 5	CDH5
599	Fibroblast Growth Factor 1	FGF1
600	Mucin 5AC, Oligomeric Mucus/Gel-Forming	MUC5AC
601	Activated Leukocyte Cell Adhesion Molecule	ALCAM
602	Interleukin 18	IL18
603	C-C Motif Chemokine Ligand 3	CCL3
604	Nuclear Receptor Subfamily 3 Group C Member 1	NR3C1
605	Stathmin 1	STMN1
606	Thrombopoietin	THPO
607	Aryl Hydrocarbon Receptor	AHR
608	Serine/Threonine Kinase 4	STK4
609	Matrix Metallopeptidase 13	MMP13
610	Dyskerin Pseudouridine Synthase 1	DKC1
611	Rho Associated Coiled-Coil Containing Protein Kinase 1	ROCK1
612	Brain Derived Neurotrophic Factor	BDNF
613	Paired Box 8	PAX8
614	Heat Shock Protein Family A (Hsp70) Member 8	HSPA8
615	Cathepsin D	CTSD
616	CD27 Molecule	CD27
617	Granzyme B	GZMB
618	Transcription Factor AP-2 Alpha	TFAP2A
619	ERCC Excision Repair 5, Endonuclease	ERCC5
620	Protein Phosphatase 2 Scaffold Subunit Aalpha	PPP2R1A
621	Receptor For Activated C Kinase 1	RACK1
622	Signal Transducer And Activator Of Transcription 5A	STAT5A
623	Annexin A1	ANXA1
624	Apoptotic Peptidase Activating Factor 1	APAF1
625	CEA Cell Adhesion Molecule 1	CEACAM1
626	Leucine Zipper Tumor Suppressor 1	LZTS1
627	C-X-C Motif Chemokine Ligand 10	CXCL10
628	Growth Differentiation Factor 15	GDF15
629	Apolipoprotein E	APOE
630	CD3 Gamma Subunit Of T-Cell Receptor Complex	CD3G
631	Fas Associated Via Death Domain	FADD
632	Protein Tyrosine Phosphatase Non-Receptor Type 12	PTPN12
633	Lysine Demethylase 4C	KDM4C
634	Interleukin 2 Receptor Subunit Beta	IL2RB
635	Reversion Inducing Cysteine Rich Protein With Kazal Motifs	RECK
636	C-X-C Motif Chemokine Receptor 3	CXCR3
637	Dual Specificity Phosphatase 1	DUSP1
638	Protein Kinase, DNA-Activated, Catalytic Subunit	PRKDC
639	Wnt Family Member 5A	WNT5A
640	Endothelin Receptor Type A	EDNRA
641	Von Willebrand Factor	VWF
642	Annexin A2	ANXA2

643	Mitogen-Activated Protein Kinase Kinase 4	MAP2K4
644	Cyclin H	CCNH
645	Neurotrophic Receptor Tyrosine Kinase 3	NTRK3
646	Phosphoinositide-3-Kinase Regulatory Subunit 2	PIK3R2
647	Interleukin 6 Cytokine Family Signal Transducer	IL6ST
648	Ubiquitin C-Terminal Hydrolase L1	UCHL1
649	Protein Kinase C Beta	PRKCB
650	Tyrosine Kinase 2	TYK2
651	CCAAT Enhancer Binding Protein Alpha	CEBPA
652	15-Hydroxyprostaglandin Dehydrogenase	HPGD
653	Suppressor Of Cytokine Signaling 1	SOCS1
654	PML Nuclear Body Scaffold	PML
655	Solute Carrier Family 19 Member 1	SLC19A1
656	Transcription Factor Binding To IGHM Enhancer 3	TFE3
657	Ras Homolog Family Member B	RHOB
658	Arachidonate 5-Lipoxygenase	ALOX5
659	ASXL Transcriptional Regulator 1	ASXL1
660	Deleted In Malignant Brain Tumors 1	DMBT1
661	Integrin Subunit Beta 2	ITGB2
662	Splicing Factor 3b Subunit 1	SF3B1
663	Baculoviral IAP Repeat Containing 7	BIRC7
664	Transcription Factor 3	TCF3
665	Nanog Homeobox	NANOG
666	Prostaglandin E Receptor 4	PTGER4
667	Diablo IAP-Binding Mitochondrial Protein	DIABLO
668	ERCC Excision Repair 3, TFIIH Core Complex Helicase Subunit	ERCC3
669	E2F Transcription Factor 2	E2F2
670	Stromal Interaction Molecule 1	STIM1
671	UDP Glucuronosyltransferase Family 1 Member A1	UGT1A1
672	C-X-C Motif Chemokine Receptor 5	CXCR5
673	CEA Cell Adhesion Molecule 7	CEACAM7
674	Major Histocompatibility Complex, Class I, C	HLA-C
675	Epoxide Hydrolase 1	EPHX1
676	CD3 Epsilon Subunit Of T-Cell Receptor Complex	CD3E
677	Selectin E	SELE
678	GATA Binding Protein 6	GATA6
679	EPH Receptor B4	EPHB4
680	Exportin 1	XPO1
681	Laminin Subunit Beta 3	LAMB3
682	Advanced Glycosylation End-Product Specific Receptor	AGER
683	ETS Transcription Factor ERG	ERG
684	Lymphoid Enhancer Binding Factor 1	LEF1
685	Toll Like Receptor 3	TLR3
686	Programmed Cell Death 1 Ligand 2	PDCD1LG2
687	Prostate Stem Cell Antigen	PSCA
688	Erythropoietin Receptor	EPOR

689	MYCL Proto-Oncogene, BHLH Transcription Factor	MYCL
690	Cyclin Dependent Kinase 5	CDK5
691	Caspase 1	CASP1
692	Sphingomyelin Phosphodiesterase 1	SMPD1
693	Bone Morphogenetic Protein Receptor Type 2	BMPR2
694	WEE1 G2 Checkpoint Kinase	WEE1
695	Fc Gamma Receptor IIa	FCGR2A
696	Sequestosome 1	SQSTM1
697	Hyaluronan Binding Protein 2	HABP2
698	Catalase	CAT
699	Dipeptidyl Peptidase 4	DPP4
700	Deoxycytidine Kinase	DCK
701	Pyruvate Kinase M1/2	PKM
702	Transforming Growth Factor Beta 3	TGFB3
703	Nerve Growth Factor	NGF
704	CD80 Molecule	CD80
705	Catechol-O-Methyltransferase	COMT
706	Delta Like Non-Canonical Notch Ligand 1	DLK1
707	Glial Fibrillary Acidic Protein	GFAP
708	Discoidin Domain Receptor Tyrosine Kinase 1	DDR1
709	Caudal Type Homeobox 2	CDX2
710	Coagulation Factor III, Tissue Factor	F3
711	Protein Kinase AMP-Activated Catalytic Subunit Alpha 1	PRKAA1
712	Thrombomodulin	THBD
713	Cyclin Dependent Kinase 12	CDK12
714	Nestin	NES
715	Cortactin	CTTN
716	Hepatocyte Nuclear Factor 4 Alpha	HNF4A
717	Cell Division Cycle 6	CDC6
718	TNF Receptor Associated Factor 2	TRAF2
719	Napsin A Aspartic Peptidase	NAPSA
720	Growth Factor Receptor Bound Protein 2	GRB2
721	Platelet Derived Growth Factor Subunit A	PDGFA
722	Calcitonin Related Polypeptide Alpha	CALCA
723	CD22 Molecule	CD22
724	Nth Like DNA Glycosylase 1	NTHL1
725	Cholecystokinin B Receptor	CCKBR
726	Filamin A	FLNA
727	Histone Deacetylase 2	HDAC2
728	Transcription Factor 7	TCF7
729	Membrane Spanning 4-Domains A1	MS4A1
730	Forkhead Box E1	FOXE1
731	Elastin	ELN
732	Adrenoceptor Beta 2	ADRB2
733	Integrin Subunit Alpha 2b	ITGA2B
734	Signal Transducer And Activator Of Transcription 6	STAT6

735	Gastrin Releasing Peptide Receptor	GRPR
736	Gastrin	GAST
737	Hydroxysteroid 17-Beta Dehydrogenase 1	HSD17B1
738	Inhibitor Of Nuclear Factor Kappa B Kinase Regulatory Subunit Gam	IKBKG
739	Jagged Canonical Notch Ligand 1	JAG1
740	Collagen Type XVIII Alpha 1 Chain	COL18A1
741	Zinc Finger And BTB Domain Containing 16	ZBTB16
742	GATA Binding Protein 4	GATA4
743	Ribosomal Protein SA	RPSA
744	LYN Proto-Oncogene, Src Family Tyrosine Kinase	LYN
745	Sterol Regulatory Element Binding Transcription Factor 1	SREBF1
746	Nuclear Receptor Corepressor 1	NCOR1
747	Forkhead Box P1	FOXP1
748	Integrin Subunit Alpha M	ITGAM
749	ATP Binding Cassette Subfamily C Member 3	ABCC3
750	MAGE Family Member A3	MAGEA3
751	Integrin Subunit Beta 4	ITGB4
752	NADH:Ubiquinone Oxidoreductase Subunit A13	NDUFA13
753	Heat Shock Transcription Factor 1	HSF1
754	Sprouty RTK Signaling Antagonist 4	SPRY4
755	ELAV Like RNA Binding Protein 1	ELAVL1
756	Desmin	DES
757	Interferon Regulatory Factor 4	IRF4
758	Matrix Metallopeptidase 12	MMP12
759	SRY-Box Transcription Factor 4	SOX4
760	Somatostatin Receptor 2	SSTR2
761	RAB, Member Of RAS Oncogene Family Like 3	RABL3
762	BCAR1 Scaffold Protein, Cas Family Member	BCAR1
763	Roundabout Guidance Receptor 1	ROBO1
764	Cyclin E2	CCNE2
765	Suppressor Of Cytokine Signaling 3	SOCS3
766	Neurotrophic Receptor Tyrosine Kinase 2	NTRK2
767	Actin Beta	ACTB
768	Tripartite Motif Containing 28	TRIM28
769	FA Complementation Group A	FANCA
770	Myosin Light Chain Kinase	MYLK
771	Phosphoinositide-3-Kinase Regulatory Subunit 3	PIK3R3
772	Paired Box 6	PAX6
773	Thyroglobulin	TG
774	Transcription Factor 4	TCF4
775	Interleukin 12B	IL12B
776	Interleukin 24	IL24
777	Semaphorin 3B	SEMA3B
778	Eukaryotic Translation Initiation Factor 2 Alpha Kinase 2	EIF2AK2
779	CD81 Molecule	CD81
780	Insulin Like Growth Factor Binding Protein 2	IGFBP2

781	Heparin Binding EGF Like Growth Factor	HBEGF
782	RELB Proto-Oncogene, NF-KB Subunit	RELB
783	ATRX Chromatin Remodeler	ATRX
784	Tight Junction Protein 1	TJP1
785	Wnt Family Member 4	WNT4
786	Retinoid X Receptor Beta	RXRB
787	Protein Tyrosine Phosphatase Non-Receptor Type 13	PTPN13
788	Calbindin 2	CALB2
789	LIF Interleukin 6 Family Cytokine	LIF
790	C-C Motif Chemokine Receptor 3	CCR3
791	C-C Motif Chemokine Ligand 11	CCL11
792	BPI Fold Containing Family A Member 1	BPIFA1
793	P21 (RAC1) Activated Kinase 1	PAK1
794	Selectin P	SELP
795	S-Phase Kinase Associated Protein 1	SKP1
796	MAGE Family Member A4	MAGEA4
797	Rac Family Small GTPase 2	RAC2
798	RNA Binding Motif Protein 6	RBM6
799	Cytochrome P450 Family 17 Subfamily A Member 1	CYP17A1
800	ADAM Metallopeptidase Domain 12	ADAM12
801	CD86 Molecule	CD86
802	Actin Alpha Cardiac Muscle 1	ACTC1
803	Nuclear Receptor Coactivator 3	NCOA3
804	Coagulation Factor II, Thrombin	F2
805	Dickkopf WNT Signaling Pathway Inhibitor 1	DKK1
806	BAG Cochaperone 1	BAG1
807	Ribonucleotide Reductase Regulatory Subunit M2	RRM2
808	Peroxiredoxin 1	PRDX1
809	Interferon Alpha 2	IFNA2
810	Purinergic Receptor P2X 7	P2RX7
811	B Cell Linker	BLNK
812	Caspase 2	CASP2
813	Interleukin 11	IL11
814	S100 Calcium Binding Protein B	S100B
815	Laminin Subunit Alpha 5	LAMA5
816	Adiponectin, C1Q And Collagen Domain Containing	ADIPOQ
817	Erythrocyte Membrane Protein Band 4.1 Like 3	EPB41L3
818	Transcriptional And Immune Response Regulator	TCIM
819	MDM4 Regulator Of P53	MDM4
820	Leptin Receptor	LEPR
821	TNF Receptor Associated Factor 3	TRAF3
822	WNT Inhibitory Factor 1	WIF1
823	CCAAT Enhancer Binding Protein Beta	CEBPB
824	Nectin Cell Adhesion Molecule 4	NECTIN4
825	Ceruloplasmin	CP
826	C-X-C Motif Chemokine Ligand 1	CXCL1

827	C-X-C Motif Chemokine Receptor 1	CXCR1
828	CXADR Ig-Like Cell Adhesion Molecule	CXADR
829	Recoverin	RCVRN
830	Gelsolin	GSN
831	Indoleamine 2,3-Dioxygenase 1	IDO1
832	Phospholipase C Gamma 2	PLCG2
833	Phosphatidylethanolamine Binding Protein 1	PEBP1
834	Cyclin A1	CCNA1
835	TNF Receptor Superfamily Member 10d	TNFRSF10D
836	Thymidine Kinase 1	TK1
837	Myosin XVIIIB	MYO18B
838	S100 Calcium Binding Protein A2	S100A2
839	N-Myc Downstream Regulated 1	NDRG1
840	CD55 Molecule (Cromer Blood Group)	CD55
841	Cathepsin L	CTSL
842	Protein Kinase CAMP-Activated Catalytic Subunit Alpha	PRKACA
843	Mesothelin	MSLN
844	Wnt Family Member 1	WNT1
845	Protein Kinase C Epsilon	PRKCE
846	Collagen Type IV Alpha 3 Chain	COL4A3
847	Prolactin	PRL
848	Nuclear Mitotic Apparatus Protein 1	NUMA1
849	CD63 Molecule	CD63
850	Lymphotoxin Alpha	LTA
851	Fli-1 Proto-Oncogene, ETS Transcription Factor	FLI1
852	TNF Receptor Associated Factor 1	TRAF1
853	Casein Kinase 1 Alpha 1	CSNK1A1
854	Bone Gamma-Carboxyglutamate Protein	BGLAP
855	Glutathione S-Transferase Mu 3	GSTM3
856	Metadherin	MTDH
857	GLI Family Zinc Finger 2	GLI2
858	Toll Like Receptor 5	TLR5
859	L1 Cell Adhesion Molecule	L1CAM
860	DEAD-Box Helicase 5	DDX5
861	Kruppel Like Factor 5	KLF5
862	Integrin Subunit Alpha L	ITGAL
863	MAGE Family Member A1	MAGEA1
864	FA Complementation Group G	FANCG
865	Lactate Dehydrogenase A	LDHA
866	Cytochrome P450 Family 1 Subfamily A Member 2	CYP1A2
867	Lysine Methyltransferase 2A	KMT2A
868	Exonuclease 1	EXO1
869	Melanoma Cell Adhesion Molecule	MCAM
870	Macrophage Stimulating 1 Receptor	MST1R
871	Laminin Subunit Alpha 1	LAMA1
872	Ryanodine Receptor 1	RYR1

873	Cadherin 3	CDH3
874	Dipeptidyl Peptidase 9	DPP9
875	Leucine Zipper Like Transcription Regulator 1	LZTR1
876	Minichromosome Maintenance Complex Component 3	MCM3
877	Laminin Subunit Gamma 1	LAMC1
878	PR/SET Domain 14	PRDM14
879	Cell Division Cycle 20	CDC20
880	Fatty Acid Binding Protein 4	FABP4
881	Interleukin 1 Receptor Type 1	IL1R1
882	Cancer/Testis Antigen 2	CTAG2
883	TPX2 Microtubule Nucleation Factor	TPX2
884	Class II Major Histocompatibility Complex Transactivator	CIITA
885	Gap Junction Protein Beta 2	GJB2
886	Nicotinamide N-Methyltransferase	NNMT
887	G Protein-Coupled Estrogen Receptor 1	GPER1
888	IKAROS Family Zinc Finger 3	IKZF3
889	Proopiomelanocortin	POMC
890	Mitogen-Activated Protein Kinase 10	MAPK10
891	RecQ Like Helicase	RECQL
892	Secreted Frizzled Related Protein 1	SFRP1
893	Ubiquitin Specific Peptidase 8	USP8
894	Neural Precursor Cell Expressed, Developmentally Down-Regulated 9	NEDD9
895	Protein Tyrosine Phosphatase Non-Receptor Type 22	PTPN22
896	Wnt Family Member 3	WNT3
897	Granulin Precursor	GRN
898	V-Set Domain Containing T Cell Activation Inhibitor 1	VTCN1
899	Interferon Regulatory Factor 8	IRF8
900	Galactosylceramidase	GALC
901	Coagulation Factor II Thrombin Receptor	F2R
902	HOP Homeobox	HOPX
903	Arginyl-TRNA Synthetase 1	RARS1
904	Serpin Family B Member 3	SERPINB3
905	Forkhead Box A1	FOXA1
906	DEAD-Box Helicase 3 X-Linked	DDX3X
907	RE1 Silencing Transcription Factor	REST
908	Protein Phosphatase 2 Regulatory Subunit Balpha	PPP2R2A
909	C-C Motif Chemokine Ligand 4	CCL4
910	3-Phosphoinositide Dependent Protein Kinase 1	PDPK1
911	Ornithine Decarboxylase 1	ODC1
912	Serine And Arginine Rich Splicing Factor 2	SRSF2
913	Spermidine/Spermine N1-Acetyltransferase 1	SAT1
914	Cancer/Testis Antigen 83	CT83
915	Microtubule Associated Protein Tau	MAPT
916	Metastasis Associated 1	MTA1
917	G Protein-Coupled Receptor Class C Group 5 Member A	GPRC5A
918	Lamin B Receptor	LBR

919	Interleukin 12A	IL12A
920	Acetylcholinesterase (Cartwright Blood Group)	ACHE
921	Nuclear Receptor Subfamily 1 Group H Member 2	NR1H2
922	FUS RNA Binding Protein	FUS
923	TNF Receptor Associated Factor 4	TRAF4
924	Activin A Receptor Like Type 1	ACVRL1
925	CD226 Molecule	CD226
926	H2A.X Variant Histone	H2AX
927	Bromodomain Containing 4	BRD4
928	Heterogeneous Nuclear Ribonucleoprotein A2/B1	HNRNPA2B1
929	Tissue Factor Pathway Inhibitor 2	TFPI2
930	Signal Peptide, CUB Domain And EGF Like Domain Containing 3	SCUBE3
931	Bone Morphogenetic Protein 7	BMP7
932	CD209 Molecule	CD209
933	Fibrinogen Alpha Chain	FGA
934	Wnt Family Member 7B	WNT7B
935	TNF Alpha Induced Protein 3	TNFAIP3
936	Selectin L	SELL
937	ADAM Metallopeptidase Domain 17	ADAM17
938	Eukaryotic Translation Initiation Factor 4E Binding Protein 1	EIF4EBP1
939	Killer Cell Immunoglobulin Like Receptor, Three Ig Domains And Lo	KIR3DL1
940	Ras Association Domain Family Member 5	RASSF5
941	GLI Family Zinc Finger 3	GLI3
942	Methylthioadenosine Phosphorylase	MTAP
943	CD82 Molecule	CD82
944	CD47 Molecule	CD47
945	CD2 Molecule	CD2
946	TTK Protein Kinase	TTK
947	Plasminogen	PLG
948	Succinate Dehydrogenase Complex Assembly Factor 2	SDHAF2
949	Pyruvate Dehydrogenase Kinase 1	PDK1
950	Casein Kinase 2 Alpha 1	CSNK2A1
951	Trefoil Factor 1	TFF1
952	Interleukin 12 Receptor Subunit Beta 1	IL12RB1
953	Sirtuin 3	SIRT3
954	NLR Family Pyrin Domain Containing 3	NLRP3
955	TNF Receptor Superfamily Member 11a	TNFRSF11A
956	Integrin Subunit Alpha E	ITGAE
957	X-Box Binding Protein 1	XBP1
958	Moesin	MSN
959	Collagen Type IV Alpha 2 Chain	COL4A2
960	Apoptosis Inducing Factor Mitochondria Associated 1	AIFM1
961	Transcription Factor EB	TFEB
962	Ectonucleotide Pyrophosphatase/Phosphodiesterase 2	ENPP2
963	BAF Chromatin Remodeling Complex Subunit BCL11A	BCL11A
964	Interleukin 9	IL9

965	DNA Damage Inducible Transcript 3	DDIT3
966	Prostaglandin-Endoperoxide Synthase 1	PTGS1
967	Amyloid Beta Precursor Protein	APP
968	Sphingosine Kinase 1	SPHK1
969	Protein Tyrosine Phosphatase Non-Receptor Type 1	PTPN1
970	Pim-1 Proto-Oncogene, Serine/Threonine Kinase	PIM1
971	5'-Nucleotidase Ecto	NT5E
972	Nerve Growth Factor Receptor	NGFR
973	Transglutaminase 2	TGM2
974	SIX Homeobox 1	SIX1
975	Timeless Circadian Regulator	TIMELESS
976	Linker For Activation Of T Cells	LAT
977	Vitronectin	VTN
978	LDL Receptor Related Protein 1B	LRP1B
979	Acid Phosphatase 5, Tartrate Resistant	ACP5
980	Transmembrane Serine Protease 2	TMPRSS2
981	Tumor Suppressor 2, Mitochondrial Calcium Regulator	TUSC2
982	ADAM Metallopeptidase Domain 15	ADAM15
983	Killer Cell Lectin Like Receptor C1	KLRC1
984	Microtubule Associated Scaffold Protein 1	MTUS1
985	LIM Domain Containing 1	LIMD1
986	Cell Division Cycle 25B	CDC25B
987	Chitinase 3 Like 1	CHI3L1
988	Nodal Growth Differentiation Factor	NODAL
989	Cell Adhesion Molecule L1 Like	CHL1
990	C-C Motif Chemokine Receptor 1	CCR1
991	Glutamate-Cysteine Ligase Catalytic Subunit	GCLC
992	S100 Calcium Binding Protein A9	S100A9
993	CD46 Molecule	CD46
994	Iron Responsive Element Binding Protein 2	IREB2
995	Forkhead Box F1	FOXF1
996	Ribosomal Oxygenase 2	RIOX2
997	Insulin Receptor Substrate 2	IRS2
998	Histone Deacetylase 4	HDAC4
999	protein phosphatase 1 regulatory subunit 12C	PPP1R12C
1000	Cas scaffold protein family member 4	CASS4
1001	proline rich protein HaeIII subfamily 1	PRH1
1002	proline rich protein HaeIII subfamily 2	PRH2
1003	mucolipin 1	MCOLN1
1004	prohibitin	PHB
1005	myocardial infarction associated transcript	MIAT
1006	matrix metallopeptidase 19	MMP19
1007	midkine	MDK
1008	cyclin dependent kinase 14	CDK14
1009	SIX homeobox 4	SIX4
1010	NADPH oxidase 4	NOX4

1011	zinc finger SWIM-type containing 5	ZSWIM5
1012	chondroitin polymerizing factor	CHPF
1013	Cbl proto-oncogene like 1	CBLL1
1014	LIM domain containing 2	LIMD2
1015	VANGL planar cell polarity protein 1	VANGL1
1016	forkhead box N1	FOXN1
1017	interferon induced transmembrane protein 1	IFITM1
1018	Rho guanine nucleotide exchange factor 7	ARHGEF7
1019	CBFA2/RUNX1 partner transcriptional co-repressor 2	CBFA2T2
1020	Pvt1 oncogene	PVT1
1021	pyrroline-5-carboxylate reductase 1	PYCR1
1022	golgi phosphoprotein 3	GOLPH3
1023	SOS Ras/Rho guanine nucleotide exchange factor 2	SOS2
1024	tetraspanin 7	TSPAN7
1025	thymopoietin	TMPO
1026	lon peptidase 1, mitochondrial	LONP1
1027	family with sequence similarity 83 member F	FAM83F
1028	ankyrin repeat domain 22	ANKRD22
1029	cytochrome P450 family 2 subfamily C member 9	CYP2C9
1030	death associated protein	DAP
1031	decorin	DCN
1032	RAD52 motif containing 1	RDM1
1033	ETS variant transcription factor 4	ETV4
1034	hepatic and glial cell adhesion molecule	HEPACAM
1035	fibroblast growth factor 9	FGF9
1036	cholinergic receptor nicotinic alpha 4 subunit	CHRNA4
1037	CD300a molecule	CD300A
1038	TOP1 binding arginine/serine rich protein	TOPORS
1039	flotillin 1	FLOT1
1040	deleted in lymphocytic leukemia 1	DLEU1
1041	Kruppel like factor 2	KLF2
1042	late endosomal/lysosomal adaptor, MAPK and MTOR activator 5	LAMTOR5
1043	C-X-C motif chemokine ligand 13	CXCL13
1044	CUGBP Elav-like family member 1	CELF1
1045	leukocyte immunoglobulin like receptor B1	LILRB1
1046	KCNQ1 opposite strand/antisense transcript 1	KCNQ1OT1
1047	ubiquitin conjugating enzyme E2 C	UBE2C
1048	protein phosphatase 1 regulatory subunit 13B	PPP1R13B
1049	interleukin 12 receptor subunit beta 2	IL12RB2
1050	interleukin enhancer binding factor 2	ILF2
1051	opioid growth factor receptor pseudogene 1	OGFRP1
1052	GA binding protein transcription factor subunit alpha	GABPA
1053	prostate androgen-regulated transcript 1	PART1
1054	chromodomain helicase DNA binding protein 5	CHD5
1055	large tumor suppressor kinase 2	LATS2
1056	nuclear paraspeckle assembly transcript 1	NEAT1

1057	HIC ZBTB transcriptional repressor 1	HIC1
1058	homeobox A11	HOXA11
1059	homeobox B5	HOXB5
1060	Bile salt export pump	ABCB11
1061	Cholinesterase	BCHE
1062	Cocaine esterase	CES2
1063	Cytochrome P450 2B6	CYP2B6
1064	Cytochrome P450 2C8	CYP2C8
1065	Cytochrome P450 3A5	CYP3A5
1066	Cytochrome P450 3A7	CYP3A7
1067	Cytosolic phospholipase A2	PLA2G4A
1068	DNA topoisomerase 2-beta	TOP2B
1069	DNA topoisomerase I, mitochondrial	TOP1MT
1070	Liver carboxylesterase 1	CES1
1071	Microtubule-associated protein 2	MAP2
1072	Microtubule-associated protein 4	MAP4
1073	Multidrug resistance-associated protein 6	ABCC6
1074	Multidrug resistance-associated protein 7	ABCC10
1075	NADPH--cytochrome P450 reductase	POR
1076	Nuclear receptor subfamily 1 group I member 2	NR1I2
1077	Solute carrier family 22 member 1	SLC22A1
1078	Solute carrier family 22 member 3	SLC22A3
1079	Solute carrier organic anion transporter family member 1B1	SLCO1B1
1080	Solute carrier organic anion transporter family member 1B3	SLCO1B3
1081	Tubulin alpha-1A chain	TUBA1A
1082	Tubulin beta-1 chain	TUBB1
1083	Tubulin delta chain	TUBD1
1084	Tubulin epsilon chain	TUBE1
1085	Tubulin gamma-1 chain	TUBG1
1086	UDP-glucuronosyltransferase 1-9	UGT1A9
1087	UDP-glucuronosyltransferase 2B7	UGT2B7
