# Supplementary material for: Integrating Network Pharmacology and Experimental Validation to Elucidate the Mechanism of Yiqi Yangyin Decoction in Suppressing Non-Small-Cell Lung Cancer
Source: Biomed Res Int. 2023 Feb 20;2023:4967544. doi: 10.1155/2023/4967544 (PMC9980286; doi:10.1155/2023/4967544)
Supplement: Supplementary 4 — Supplementary Table 3: the overlapping genes for compound targets and NSCLC-related targets. [file 4967544.f4.pdf]

Supplementay Table 3: The overlapping genes for compound targets and NSCLC-related targets

Number	Protein name	Gene name
1	Progesterone receptor	PGR
2	Prostaglandin G/H synthase 1	PTGS1
3	Prostaglandin G/H synthase 2	PTGS2
4	Heat shock protein HSP 90	HSP90AA1
5	Phosphatidylinositol-4,5-bisphosphate 3-kinase catalytic subunit, gamma isoform	PIK3CG
6	mRNA of PKA Catalytic Subunit C-alpha	PRKACA
7	Beta-2 adrenergic receptor	ADRB2
8	Apoptosis regulator Bcl-2	BCL2
9	Apoptosis regulator BAX	BAX
10	Caspase-9	CASP9
11	Transcription factor AP-1	JUN
12	Caspase-3	CASP3
13	Caspase-8	CASP8
14	Protein kinase C alpha type	PRKCA
15	Transforming growth factor beta-1	TGFB1
16	Microtubule-associated protein 2	MAP2
17	Androgen receptor	AR
18	Cytochrome P450 17A1	CYP17A1
19	Estrogen receptor	ESR1
20	Aromatase	CYP19A1
21	Estrogen receptor beta	ESR2
22	Glucocorticoid receptor	NR3C1
23	M-phase inducer phosphatase 2	CDC25B
24	Acetylcholinesterase	ACHE
25	Bone morphogenetic protein 2	BMP2
26	Proto-oncogene serine/threonine-protein kinase Pim-1	PIM1
27	Serum albumin	ALB
28	Estradiol 17-beta-dehydrogenase 1	HSD17B1
29	Caspase-7	CASP7
30	Mitogen-activated protein kinase 1	MAPK1
31	Collagenase 3	MMP13
32	Prothrombin	F2
33	Vascular endothelial growth factor receptor 2	KDR
34	Peroxisome proliferator-activated receptor gamma	PPARG
35	Cell division protein kinase 2	CDK2
36	Mitogen-activated protein kinase 14	MAPK14
37	Mitogen-activated protein kinase 8	MAPK8
38	Nitric oxide synthase, endothelial	NOS3
39	Epidermal growth factor receptor	EGFR
40	ADAM 17	ADAM17
41	Annexin A5	ANXA5
42	Proto-oncogene tyrosine-protein kinase Src	SRC

43	3-phosphoinositide-dependent protein kinase 1	PDPK1
44	Tyrosine-protein phosphatase non-receptor type 11	PTPN11
45	Liver carboxylesterase 1	CES1
46	Serine/threonine-protein kinase Chk1	CHEK1
47	TGF-beta receptor type-1	TGFBR1
48	Integrin alpha-L	ITGAL
49	Arachidonate 5-lipoxygenase	ALOX5
50	Phospholipase A2, membrane associated	PLA2G2A
51	Histone deacetylase 4	HDAC4
52	Cocaine esterase	CES2
53	Toll-like receptor 9	TLR9
54	Histone deacetylase 2	HDAC2
55	Macrophage migration inhibitory factor	MIF
56	Retinoic acid receptor beta	RARB
57	Aryl hydrocarbon receptor	AHR
58	Glutathione S-transferase P	GSTP1
59	Ephrin type-B receptor 4	EPHB4
60	Mitogen-activated protein kinase 10	MAPK10
61	Cholinesterase	BCHE
62	Cyclin-A2	CCNA2
63	B-Raf proto-oncogene serine/threonine-protein kinase	BRAF
64	Insulin-like growth factor 1 receptor	IGF1R
65	Proto-oncogene tyrosine-protein kinase LCK	LCK
66	Retinoic acid receptor RXR-alpha	RXRA
67	NAD(P)H dehydrogenase [quinone] 1	NQO1
68	Thymidylate synthase	TYMS
69	Cathepsin B	CTSB
70	Prostaglandin E2 receptor EP4 subtype	PTGER4
71	Tyrosine-protein phosphatase non-receptor type 22	PTPN22
72	Stromelysin-1	MMP3
73	Oxysterols receptor LXR-beta	NR1H2
74	Dipeptidyl peptidase IV	DPP4
75	Tyrosine-protein phosphatase non-receptor type 1	PTPN1
76	DNA (cytosine-5)-methyltransferase 1	DNMT1
77	Heat shock cognate 71 kDa protein	HSPA8
78	Nitric oxide synthase, inducible	NOS2
79	DNA topoisomerase II	TOP2A
80	Transcription factor p65	RELA
81	Inhibitor of nuclear factor kappa-B kinase subunit beta	IKBKB
82	RAC-alpha serine/threonine-protein kinase	AKT1
83	Tumor necrosis factor	TNF
84	Interstitial collagenase	MMP1
85	Signal transducer and activator of transcription 1-alpha/beta	STAT1
86	Cell division control protein 2 homolog	CDK1
87	Heme oxygenase 1	HMOX1
88	Cytochrome P450 3A4	CYP3A4

89	Cytochrome P450 1A2	CYP1A2
90	Cytochrome P450 1A1	CYP1A1
91	Intercellular adhesion molecule 1	ICAM1
92	E-selectin	SELE
93	Nuclear receptor subfamily 1 group I member 2	NR1I2
94	Cytochrome P450 1B1	CYP1B1
95	Insulin receptor	INSR
96	Glutathione S-transferase Mu 1	GSTM1
97	NADPH oxidase 4	NOX4
98	Tyrosine-protein kinase receptor FLT3	FLT3
99	Multidrug resistance-associated protein 1	ABCC1
100	P-glycoprotein 1	ABCB1
101	ATP-binding cassette sub-family G member 2	ABCG2
102	Tyrosine-protein kinase SYK	SYK
103	Glycogen synthase kinase-3 beta	GSK3B
104	Matrix metalloproteinase 9	MMP9
105	Matrix metalloproteinase 2	MMP2
106	Cyclin-dependent kinase 5	CDK5
107	cyclin B1	CCNB1
108	Death-associated protein kinase 1	DAPK1
109	Receptor-type tyrosine-protein phosphatase C	PTPRC
110	1	MCL1
111	Telomerase reverse transcriptase	TERT
112	Carbonic anhydrase 9	CA9
113	Tubulin alpha-1A chain	TUBA1A
114	Hepatocyte nuclear factor 4-alpha	HNF4A
115	Ras-related C3 botulinum toxin substrate 1	RAC1
116	Cytochrome P450 2C9	CYP2C9
117	Protein kinase C epsilon type	PRKCE
118	Amyloid beta A4 protein	APP
119	Cell division protein kinase 6	CDK6
120	Dihydrofolate reductase	DHFR
121	Serine/threonine-protein kinase 6	AURKA
122	Vascular endothelial growth factor A	VEGFA
123	Estrogen receptor	CCND1
124	Bcl-2-like protein 1	BCL2L1
125	Cyclin-dependent kinase inhibitor 1	CDKN1A
126	Interleukin-10	IL10
127	Retinoblastoma-associated protein	RB1
128	Cell division protein kinase 4	CDK4
129	Interleukin-6	IL6
130	Cellular tumor antigen p53	TP53
131	NF-kappa-B inhibitor alpha	NFKBIA
132	DNA topoisomerase 1	TOP1
133	E3 ubiquitin-protein ligase Mdm2	MDM2
134	Proliferating cell nuclear antigen	PCNA

135	Receptor tyrosine-protein kinase erbB-2	ERBB2
136	Baculoviral IAP repeat-containing protein 5	BIRC5
137	Interleukin-2	IL2
138	Interferon gamma	IFNG
139	Interleukin-4	IL4
140	CD40 ligand	CD40LG
141	Hepatocyte growth factor receptor	MET
142	Poly [ADP-ribose] polymerase 1	PARP1
143	Macrophage metalloelastase	MMP12
144	Cathepsin D	CTSD
145	Proto-oncogene c-Fos	FOS
146	Urokinase-type plasminogen activator	PLAU
147	Pro-epidermal growth factor	EGF
148	Cyclin-dependent kinase inhibitor 2A, isoforms 1/2/3	CDKN2A
149	NADPH--cytochrome P450 reductase	POR
150	Ornithine decarboxylase	ODC1
151	RAF proto-oncogene serine/threonine-protein kinase	RAF1
152	Superoxide dismutase [Cu-Zn]	SOD1
153	Hypoxia-inducible factor 1-alpha	HIF1A
154	78 kDa glucose-regulated protein	HSPA5
155	Caveolin-1	CAV1
156	Myc proto-oncogene protein	MYC
157	Tissue factor	F3
158	Gap junction alpha-1 protein	GJA1
159	Interleukin-1 beta	IL1B
160	C-C motif chemokine 2	CCL2
161	Interleukin-8	CXCL8
162	Protein kinase C beta type	PRKCB
163	Heat shock protein beta-1	HSPB1
164	Thrombomodulin	THBD
165	Plasminogen activator inhibitor 1	SERPINE1
166	Collagen alpha-1(I) chain	COL1A1
167	Phosphatidylinositol-3,4,5-trisphosphate 3-phosphatase and dual-specificity protein phosphatase PTEN	PTEN
168	Interleukin-1 alpha	IL1A
169	Myeloperoxidase	MPO
170	Nuclear factor erythroid 2-related factor 2	NFE2L2
171	Serine/threonine-protein kinase Chk2	CHEK2
172	Heat shock factor protein 1	HSF1
173	C-reactive protein	CRP
174	C-X-C motif chemokine 10	CXCL10
175	Inhibitor of nuclear factor kappa-B kinase subunit alpha	CHUK
176	Osteopontin	SPP1
177	Runt-related transcription factor 2	RUNX2
178	Ras association domain-containing protein 1	RASSF1
179	Transcription factor E2F1	E2F1

180	Transcription factor E2F2	E2F2
181	Insulin-like growth factor-binding protein 3	IGFBP3
182	Insulin-like growth factor II	IGF2
183	Interferon regulatory factor 1	IRF1
184	Receptor tyrosine-protein kinase erbB-3	ERBB3
185	Ras GTPase-activating protein 1	RASA1
186	Serine/threonine-protein kinase Aurora-B	AURKB
187	PI3-kinase p85-alpha subunit	PIK3R1
188	Focal adhesion kinase 1	PTK2
189	Serine/threonine-protein kinase PLK1	PLK1
190	Casein kinase II subunit alpha	CSNK2A1
191	C-X-C chemokine receptor type 1	CXCR1
192	ALK tyrosine kinase receptor	ALK
193	Tyrosine-protein kinase receptor UFO	AXL
194	Microtubule-associated protein tau	MAPT
195	Myosin light chain kinase, smooth muscle	MYLK
196	DNA-(apurinic or apyrimidinic site) lyase	APEX1
197	Basic fibroblast growth factor receptor 1	FGFR1
198	Bone morphogenetic protein 7	BMP7
199	S-methyl-5-thioadenosine phosphorylase	MTAP
200	Retinoic acid receptor RXR-beta	RXRB
201	Retinoic acid receptor alpha	RARA
202	Receptor tyrosine-protein kinase erbB-4	ERBB4
203	Fatty acid-binding protein, adipocyte	FABP4
204	Smoothened homolog	SMO
205	Neuronal acetylcholine receptor subunit alpha-4	CHRNA4
206	P-selectin	SELP
207	SPARC	SPARC
208	C-X-C chemokine receptor type 2	CXCR2
209	Dual specificity mitogen-activated protein kinase kinase 1	MAP2K1
210	Thrombopoietin receptor	MPL
211	TGF-beta receptor type-2	TGFBR2
212	Heparin-binding growth factor 1	FGF1
213	Thymidine phosphorylase	TYMP
214	Angiotensin-converting enzyme	ACE
215	Caspase-1	CASP1
216	Dual specificity mitogen-activated protein kinase kinase 4	MAP2K4
217	Proto-oncogene tyrosine-protein kinase receptor Ret	RET
218	Superoxide dismutase [Mn], mitochondrial	SOD2
219	Fibroblast growth factor receptor 2	FGFR2
220	Endothelin-1 receptor	EDNRA
221	Plasminogen	PLG
222	Fatty acid synthase	FASN
223	Catalase	CAT
224	Cytosolic phospholipase A2	PLA2G4A
225	Canalicular multispecific organic anion transporter 1	ABCC2

226	Serine/threonine-protein kinase mTOR	MTOR
227	Catenin alpha-1	CTNNA1
228	Non-receptor tyrosine-protein kinase TYK2	TYK2
229	Cytochrome c	CYCS
