# Supplementary material for: Integrating Network Pharmacology and Experimental Validation to Elucidate the Mechanism of Yiqi Yangyin Decoction in Suppressing Non-Small-Cell Lung Cancer
Source: Biomed Res Int. 2023 Feb 20;2023:4967544. doi: 10.1155/2023/4967544 (PMC9980286; doi:10.1155/2023/4967544)
Supplement: Supplementary 5 — Supplementary Table 4: parameters of the core genes in PPI network. [file 4967544.f5.pdf]

Supplementary Table 4 Parameters of the core genes in the PPI Network

Number	Gene	DC	BC	CC
1	AKT1	33	47.02234	0.88372093
2	SRC	30	34.479958	0.82608694
3	JUN	30	32.362465	0.82608694
4	TP53	30	32.02045	0.82608694
5	EGFR	29	27.65256	0.80851066
6	MYC	28	28.91983	0.7916667
7	STAT1	27	27.851223	0.7755102
8	ESR1	26	24.202396	0.76
9	HSP90AA1	26	26.196888	0.76
10	CASP3	26	26.26666	0.76
11	EGF	26	23.183254	0.76
12	MAPK1	26	22.012373	0.76
13	VEGFA	25	22.767511	0.74509805
14	IL6	23	15.678348	0.7169811
15	RELA	23	17.538237	0.7169811
16	HIF1A	22	12.680949	0.7037037
17	TNF	22	14.453227	0.7037037
18	PTEN	22	12.434376	0.7037037
19	MAPK14	22	11.511389	0.7037037
20	ERBB2	21	15.617214	0.6909091
21	PIK3R1	21	17.144556	0.6909091
22	CCND1	21	9.324182	0.6909091
23	MAPK8	20	12.671127	0.6785714
24	FOS	20	9.305122	0.6785714
25	PTK2	19	10.488007	0.6666667
26	IL2	19	10.71837	0.6666667
27	PTPN11	18	10.135038	0.6551724
28	BCL2L1	18	10.404648	0.6551724
29	MAP2K1	17	13.036323	0.6440678
30	IL1B	17	6.963662	0.6440678
31	NFKBIA	17	7.34223	0.6440678
32	CAV1	16	9.695935	0.6333333
33	LCK	16	5.6131086	0.6333333
34	PPARG	16	7.70334	0.6333333
35	MDM2	15	3.9239686	0.6229508
36	PRKACA	14	8.503685	0.61290324
37	CDKN1A	14	2.8582258	0.61290324
38	ALB	14	4.2802677	0.61290324
39	GSK3B	13	5.0365524	0.6031746
