# Supplementary material for: Integrating Network Pharmacology and Experimental Validation to Elucidate the Mechanism of Yiqi Yangyin Decoction in Suppressing Non-Small-Cell Lung Cancer
Source: Biomed Res Int. 2023 Feb 20;2023:4967544. doi: 10.1155/2023/4967544 (PMC9980286; doi:10.1155/2023/4967544)
Supplement: Supplementary 6 — Supplementary Table 5: the detailed information for GO terms with p < 0.05 and count ≥ 2. [file 4967544.f6.pdf]

Supplementay Table 5 The detailed information for GO terms with *P* < 0.05 and Count ⩾ 2

Category	ID	Count	PValue	%
GOTERM_BP_DIRECT	GO:0010628	20	1.89E-20	51.28205
GOTERM_BP_DIRECT	GO:0043066	19	1.06E-18	48.71795
GOTERM_BP_DIRECT	GO:0045893	18	5.68E-15	46.15385
GOTERM_BP_DIRECT	GO:0042493	14	6.96E-15	35.89744
GOTERM_BP_DIRECT	GO:0045944	20	1.26E-13	51.28205
GOTERM_BP_DIRECT	GO:1902895	8	4.51E-12	20.51282
GOTERM_BP_DIRECT	GO:0071456	10	5.97E-12	25.64103
GOTERM_BP_DIRECT	GO:0008284	14	2.01E-11	35.89744
GOTERM_BP_DIRECT	GO:0009410	10	7.71E-10	25.64103
GOTERM_BP_DIRECT	GO:0001934	9	6.23E-09	23.07692
GOTERM_BP_DIRECT	GO:0034614	6	9.48E-09	15.38462
GOTERM_BP_DIRECT	GO:0043406	7	1.06E-08	17.94872
GOTERM_BP_DIRECT	GO:0031663	6	1.25E-08	15.38462
GOTERM_BP_DIRECT	GO:0071276	6	1.62E-08	15.38462
GOTERM_BP_DIRECT	GO:0019221	8	2.05E-08	20.51282
GOTERM_BP_DIRECT	GO:0033138	7	2.30E-08	17.94872
GOTERM_BP_DIRECT	GO:0006468	11	3.19E-08	28.20513
GOTERM_BP_DIRECT	GO:0007173	6	4.08E-08	15.38462
GOTERM_BP_DIRECT	GO:0035994	5	6.11E-08	12.82051
GOTERM_BP_DIRECT	GO:0032869	7	6.82E-08	17.94872
GOTERM_BP_DIRECT	GO:0060252	5	1.11E-07	12.82051
GOTERM_BP_DIRECT	GO:0051091	7	1.15E-07	17.94872
GOTERM_BP_DIRECT	GO:0010629	9	1.26E-07	23.07692
GOTERM_BP_DIRECT	GO:0051726	9	1.53E-07	23.07692
GOTERM_BP_DIRECT	GO:0051897	7	1.78E-07	17.94872
GOTERM_BP_DIRECT	GO:0043065	9	2.04E-07	23.07692
GOTERM_BP_DIRECT	GO:0010573	4	2.47E-07	10.25641
GOTERM_BP_DIRECT	GO:0048661	6	2.51E-07	15.38462
GOTERM_BP_DIRECT	GO:0018107	6	2.70E-07	15.38462
GOTERM_BP_DIRECT	GO:0070374	8	2.75E-07	20.51282
GOTERM_BP_DIRECT	GO:0043627	6	2.89E-07	15.38462
GOTERM_BP_DIRECT	GO:0018108	7	2.90E-07	17.94872
GOTERM_BP_DIRECT	GO:0007165	14	4.68E-07	35.89744
GOTERM_BP_DIRECT	GO:0001525	8	5.40E-07	20.51282
GOTERM_BP_DIRECT	GO:0030335	8	6.00E-07	20.51282
GOTERM_BP_DIRECT	GO:0010888	4	8.43E-07	10.25641
GOTERM_BP_DIRECT	GO:0031295	5	1.24E-06	12.82051
GOTERM_BP_DIRECT	GO:0018105	7	1.37E-06	17.94872
GOTERM_BP_DIRECT	GO:0046777	7	1.37E-06	17.94872
GOTERM_BP_DIRECT	GO:0097421	5	1.37E-06	12.82051
GOTERM_BP_DIRECT	GO:1903800	4	1.54E-06	10.25641
GOTERM_BP_DIRECT	GO:0000122	12	1.67E-06	30.76923
GOTERM_BP_DIRECT	GO:0071222	7	1.98E-06	17.94872

GOTERM_BP_DIRECT	GO:0006915	10	2.08E-06	25.64103
GOTERM_BP_DIRECT	GO:0071364	5	2.16E-06	12.82051
GOTERM_BP_DIRECT	GO:0045429	5	2.56E-06	12.82051
GOTERM_BP_DIRECT	GO:0034097	5	3.01E-06	12.82051
GOTERM_BP_DIRECT	GO:0090398	5	3.80E-06	12.82051
GOTERM_BP_DIRECT	GO:0007507	7	4.01E-06	17.94872
GOTERM_BP_DIRECT	GO:0033209	5	4.09E-06	12.82051
GOTERM_BP_DIRECT	GO:0048146	5	4.72E-06	12.82051
GOTERM_BP_DIRECT	GO:0000165	6	5.05E-06	15.38462
GOTERM_BP_DIRECT	GO:0060749	4	5.67E-06	10.25641
GOTERM_BP_DIRECT	GO:0007249	5	6.21E-06	12.82051
GOTERM_BP_DIRECT	GO:2000811	4	6.72E-06	10.25641
GOTERM_BP_DIRECT	GO:2000573	4	6.72E-06	10.25641
GOTERM_BP_DIRECT	GO:0046827	4	6.72E-06	10.25641
GOTERM_BP_DIRECT	GO:0006366	7	6.96E-06	17.94872
GOTERM_BP_DIRECT	GO:0009612	5	7.06E-06	12.82051
GOTERM_BP_DIRECT	GO:0010165	4	7.90E-06	10.25641
GOTERM_BP_DIRECT	GO:0007611	5	8.51E-06	12.82051
GOTERM_BP_DIRECT	GO:0043154	5	1.02E-05	12.82051
GOTERM_BP_DIRECT	GO:0070141	3	1.14E-05	7.692308
GOTERM_BP_DIRECT	GO:0070301	5	1.34E-05	12.82051
GOTERM_BP_DIRECT	GO:0032091	5	1.42E-05	12.82051
GOTERM_BP_DIRECT	GO:0006974	7	1.54E-05	17.94872
GOTERM_BP_DIRECT	GO:0001938	5	1.57E-05	12.82051
GOTERM_BP_DIRECT	GO:0051000	4	1.79E-05	10.25641
GOTERM_BP_DIRECT	GO:0014068	5	2.12E-05	12.82051
GOTERM_BP_DIRECT	GO:1990646	3	2.27E-05	7.692308
GOTERM_BP_DIRECT	GO:0035556	8	2.45E-05	20.51282
GOTERM_BP_DIRECT	GO:0071260	5	2.55E-05	12.82051
GOTERM_BP_DIRECT	GO:0048010	4	2.77E-05	10.25641
GOTERM_BP_DIRECT	GO:0071480	4	3.07E-05	10.25641
GOTERM_BP_DIRECT	GO:0046677	4	3.38E-05	10.25641
GOTERM_BP_DIRECT	GO:0050731	5	3.61E-05	12.82051
GOTERM_BP_DIRECT	GO:0007568	6	3.78E-05	15.38462
GOTERM_BP_DIRECT	GO:0035924	4	4.07E-05	10.25641
GOTERM_BP_DIRECT	GO:0097192	4	4.44E-05	10.25641
GOTERM_BP_DIRECT	GO:0007179	5	4.59E-05	12.82051
GOTERM_BP_DIRECT	GO:0006606	5	4.95E-05	12.82051
GOTERM_BP_DIRECT	GO:0061419	3	5.66E-05	7.692308
GOTERM_BP_DIRECT	GO:2001240	4	5.70E-05	10.25641
GOTERM_BP_DIRECT	GO:0046326	4	6.17E-05	10.25641
GOTERM_BP_DIRECT	GO:0050729	5	6.41E-05	12.82051
GOTERM_BP_DIRECT	GO:0032355	5	6.87E-05	12.82051
GOTERM_BP_DIRECT	GO:0030316	4	7.18E-05	10.25641
GOTERM_BP_DIRECT	GO:0042593	5	7.36E-05	12.82051
GOTERM_BP_DIRECT	GO:0042307	4	8.88E-05	10.25641

GOTERM_BP_DIRECT	GO:0014065	4	9.50E-05	10.25641
GOTERM_BP_DIRECT	GO:0042981	6	1.04E-04	15.38462
GOTERM_BP_DIRECT	GO:0038134	3	1.05E-04	7.692308
GOTERM_BP_DIRECT	GO:0007595	4	1.08E-04	10.25641
GOTERM_BP_DIRECT	GO:0051591	4	1.15E-04	10.25641
GOTERM_BP_DIRECT	GO:0045737	4	1.23E-04	10.25641
GOTERM_BP_DIRECT	GO:0007169	5	1.30E-04	12.82051
GOTERM_BP_DIRECT	GO:2000641	3	1.35E-04	7.692308
GOTERM_BP_DIRECT	GO:0043491	4	1.55E-04	10.25641
GOTERM_BP_DIRECT	GO:0070371	4	1.73E-04	10.25641
GOTERM_BP_DIRECT	GO:1904707	4	1.73E-04	10.25641
GOTERM_BP_DIRECT	GO:0071407	4	1.73E-04	10.25641
GOTERM_BP_DIRECT	GO:0071356	5	1.77E-04	12.82051
GOTERM_BP_DIRECT	GO:1903078	4	1.93E-04	10.25641
GOTERM_BP_DIRECT	GO:0060020	3	2.06E-04	7.692308
GOTERM_BP_DIRECT	GO:0009651	3	2.06E-04	7.692308
GOTERM_BP_DIRECT	GO:1903799	3	2.06E-04	7.692308
GOTERM_BP_DIRECT	GO:0032495	3	2.47E-04	7.692308
GOTERM_BP_DIRECT	GO:0008285	7	2.57E-04	17.94872
GOTERM_BP_DIRECT	GO:0043525	4	2.59E-04	10.25641
GOTERM_BP_DIRECT	GO:0030168	4	2.72E-04	10.25641
GOTERM_BP_DIRECT	GO:0043410	5	2.72E-04	12.82051
GOTERM_BP_DIRECT	GO:0032757	4	2.85E-04	10.25641
GOTERM_BP_DIRECT	GO:0051974	3	2.92E-04	7.692308
GOTERM_BP_DIRECT	GO:0048011	3	2.92E-04	7.692308
GOTERM_BP_DIRECT	GO:0035690	4	3.11E-04	10.25641
GOTERM_BP_DIRECT	GO:0006006	4	3.11E-04	10.25641
GOTERM_BP_DIRECT	GO:0030154	8	3.22E-04	20.51282
GOTERM_BP_DIRECT	GO:1904996	3	3.40E-04	7.692308
GOTERM_BP_DIRECT	GO:0006357	12	3.50E-04	30.76923
GOTERM_BP_DIRECT	GO:1901224	4	3.54E-04	10.25641
GOTERM_BP_DIRECT	GO:0007623	4	3.54E-04	10.25641
GOTERM_BP_DIRECT	GO:0050679	4	3.85E-04	10.25641
GOTERM_BP_DIRECT	GO:1904385	3	3.92E-04	7.692308
GOTERM_BP_DIRECT	GO:0051402	4	4.17E-04	10.25641
GOTERM_BP_DIRECT	GO:0071300	4	4.17E-04	10.25641
GOTERM_BP_DIRECT	GO:0001666	5	4.43E-04	12.82051
GOTERM_BP_DIRECT	GO:0034116	3	4.47E-04	7.692308
GOTERM_BP_DIRECT	GO:0051146	3	4.47E-04	7.692308
GOTERM_BP_DIRECT	GO:0048511	4	4.69E-04	10.25641
GOTERM_BP_DIRECT	GO:0008283	5	4.81E-04	12.82051
GOTERM_BP_DIRECT	GO:0048143	3	5.06E-04	7.692308
GOTERM_BP_DIRECT	GO:0045727	4	5.84E-04	10.25641
GOTERM_BP_DIRECT	GO:0030224	3	6.35E-04	7.692308
GOTERM_BP_DIRECT	GO:0071391	3	6.35E-04	7.692308
GOTERM_BP_DIRECT	GO:2000060	3	6.35E-04	7.692308

GOTERM_BP_DIRECT	GO:0010039	3	6.35E-04	7.692308
GOTERM_BP_DIRECT	GO:0098586	4	6.48E-04	10.25641
GOTERM_BP_DIRECT	GO:0009636	4	6.70E-04	10.25641
GOTERM_BP_DIRECT	GO:0051412	3	7.05E-04	7.692308
GOTERM_BP_DIRECT	GO:0071375	3	7.05E-04	7.692308
GOTERM_BP_DIRECT	GO:1902004	3	7.78E-04	7.692308
GOTERM_BP_DIRECT	GO:0030307	4	7.89E-04	10.25641
GOTERM_BP_DIRECT	GO:0038083	3	8.54E-04	7.692308
GOTERM_BP_DIRECT	GO:0001836	3	8.54E-04	7.692308
GOTERM_BP_DIRECT	GO:0006977	3	8.54E-04	7.692308
GOTERM_BP_DIRECT	GO:0045892	7	9.66E-04	17.94872
GOTERM_BP_DIRECT	GO:0050768	3	0.001018	7.692308
GOTERM_BP_DIRECT	GO:0051247	3	0.001018	7.692308
GOTERM_BP_DIRECT	GO:0042060	4	0.001094	10.25641
GOTERM_BP_DIRECT	GO:0032755	4	0.001094	10.25641
GOTERM_BP_DIRECT	GO:0006954	6	0.001215	15.38462
GOTERM_BP_DIRECT	GO:0090263	4	0.001289	10.25641
GOTERM_BP_DIRECT	GO:0010875	3	0.00129	7.692308
GOTERM_BP_DIRECT	GO:2001243	3	0.001388	7.692308
GOTERM_BP_DIRECT	GO:0070372	3	0.001489	7.692308
GOTERM_BP_DIRECT	GO:1900017	3	0.001489	7.692308
GOTERM_BP_DIRECT	GO:0045840	3	0.001593	7.692308
GOTERM_BP_DIRECT	GO:0010575	3	0.001593	7.692308
GOTERM_BP_DIRECT	GO:0042327	3	0.001701	7.692308
GOTERM_BP_DIRECT	GO:0045740	3	0.001812	7.692308
GOTERM_BP_DIRECT	GO:0045736	3	0.001926	7.692308
GOTERM_BP_DIRECT	GO:0043388	3	0.001926	7.692308
GOTERM_BP_DIRECT	GO:0045648	3	0.002044	7.692308
GOTERM_BP_DIRECT	GO:0048144	3	0.002044	7.692308
GOTERM_BP_DIRECT	GO:0051973	3	0.002044	7.692308
GOTERM_BP_DIRECT	GO:0048147	3	0.002044	7.692308
GOTERM_BP_DIRECT	GO:2000379	3	0.002165	7.692308
GOTERM_BP_DIRECT	GO:0030010	3	0.002165	7.692308
GOTERM_BP_DIRECT	GO:0045471	4	0.002277	10.25641
GOTERM_BP_DIRECT	GO:0010975	3	0.002418	7.692308
GOTERM_BP_DIRECT	GO:0032570	3	0.002418	7.692308
GOTERM_BP_DIRECT	GO:2001244	3	0.002418	7.692308
GOTERM_BP_DIRECT	GO:0045907	3	0.002549	7.692308
GOTERM_BP_DIRECT	GO:0048870	3	0.002549	7.692308
GOTERM_BP_DIRECT	GO:0001890	3	0.002683	7.692308
GOTERM_BP_DIRECT	GO:0010634	3	0.002683	7.692308
GOTERM_BP_DIRECT	GO:0001937	3	0.002683	7.692308
GOTERM_BP_DIRECT	GO:0070555	3	0.002821	7.692308
GOTERM_BP_DIRECT	GO:0071479	3	0.002962	7.692308
GOTERM_BP_DIRECT	GO:0009409	3	0.003106	7.692308
GOTERM_BP_DIRECT	GO:0030217	3	0.003106	7.692308

GOTERM_BP_DIRECT	GO:0001541	3	0.003405	7.692308
GOTERM_BP_DIRECT	GO:0032722	3	0.003559	7.692308
GOTERM_BP_DIRECT	GO:1904706	3	0.003559	7.692308
GOTERM_BP_DIRECT	GO:0043407	3	0.003559	7.692308
GOTERM_BP_DIRECT	GO:0032496	4	0.003574	10.25641
GOTERM_BP_DIRECT	GO:0043524	4	0.003704	10.25641
GOTERM_BP_DIRECT	GO:0034198	3	0.003716	7.692308
GOTERM_BP_DIRECT	GO:0051092	4	0.003837	10.25641
GOTERM_BP_DIRECT	GO:2000272	3	0.003876	7.692308
GOTERM_BP_DIRECT	GO:0050796	3	0.003876	7.692308
GOTERM_BP_DIRECT	GO:0003151	3	0.00404	7.692308
GOTERM_BP_DIRECT	GO:0042127	4	0.004112	10.25641
GOTERM_BP_DIRECT	GO:0050673	3	0.004206	7.692308
GOTERM_BP_DIRECT	GO:0043536	3	0.004376	7.692308
GOTERM_BP_DIRECT	GO:0008630	3	0.004376	7.692308
GOTERM_BP_DIRECT	GO:0048013	3	0.004549	7.692308
GOTERM_BP_DIRECT	GO:1900087	3	0.004549	7.692308
GOTERM_BP_DIRECT	GO:0030182	4	0.00485	10.25641
GOTERM_BP_DIRECT	GO:0034605	3	0.004904	7.692308
GOTERM_BP_DIRECT	GO:0042542	3	0.004904	7.692308
GOTERM_BP_DIRECT	GO:0031334	3	0.005272	7.692308
GOTERM_BP_DIRECT	GO:0016241	3	0.005272	7.692308
GOTERM_BP_DIRECT	GO:0014823	3	0.005461	7.692308
GOTERM_BP_DIRECT	GO:0031281	2	0.005909	5.128205
GOTERM_BP_DIRECT	GO:0060559	2	0.005909	5.128205
GOTERM_BP_DIRECT	GO:0086098	2	0.005909	5.128205
GOTERM_BP_DIRECT	GO:0021697	2	0.005909	5.128205
GOTERM_BP_DIRECT	GO:0071230	3	0.006044	7.692308
GOTERM_BP_DIRECT	GO:0034644	3	0.006044	7.692308
GOTERM_BP_DIRECT	GO:0045785	3	0.006044	7.692308
GOTERM_BP_DIRECT	GO:0007254	3	0.006245	7.692308
GOTERM_BP_DIRECT	GO:0008286	3	0.006245	7.692308
GOTERM_BP_DIRECT	GO:0051592	3	0.006655	7.692308
GOTERM_BP_DIRECT	GO:0045860	3	0.006865	7.692308
GOTERM_BP_DIRECT	GO:0002931	3	0.007078	7.692308
GOTERM_BP_DIRECT	GO:0071466	3	0.007294	7.692308
GOTERM_BP_DIRECT	GO:0042752	3	0.007512	7.692308
GOTERM_BP_DIRECT	GO:0000320	2	0.007871	5.128205
GOTERM_BP_DIRECT	GO:0033590	2	0.007871	5.128205
GOTERM_BP_DIRECT	GO:0090400	2	0.007871	5.128205
GOTERM_BP_DIRECT	GO:0038127	2	0.007871	5.128205
GOTERM_BP_DIRECT	GO:0090170	2	0.007871	5.128205
GOTERM_BP_DIRECT	GO:0072717	2	0.007871	5.128205
GOTERM_BP_DIRECT	GO:0030730	2	0.007871	5.128205
GOTERM_BP_DIRECT	GO:0051384	3	0.007958	7.692308
GOTERM_BP_DIRECT	GO:0010595	3	0.007958	7.692308

GOTERM_BP_DIRECT	GO:0030163	3	0.007958	7.692308
GOTERM_BP_DIRECT	GO:0042531	3	0.008186	7.692308
GOTERM_BP_DIRECT	GO:0001701	4	0.008468	10.25641
GOTERM_BP_DIRECT	GO:0033674	3	0.009124	7.692308
GOTERM_BP_DIRECT	GO:0050821	4	0.009247	10.25641
GOTERM_BP_DIRECT	GO:0000082	3	0.009366	7.692308
GOTERM_BP_DIRECT	GO:0051222	2	0.009829	5.128205
GOTERM_BP_DIRECT	GO:0060574	2	0.009829	5.128205
GOTERM_BP_DIRECT	GO:0031622	2	0.009829	5.128205
GOTERM_BP_DIRECT	GO:0072584	2	0.009829	5.128205
GOTERM_BP_DIRECT	GO:1905278	2	0.009829	5.128205
GOTERM_BP_DIRECT	GO:0007584	3	0.009858	7.692308
GOTERM_BP_DIRECT	GO:0051607	4	0.010553	10.25641
GOTERM_BP_DIRECT	GO:0032729	3	0.010618	7.692308
GOTERM_BP_DIRECT	GO:0032436	3	0.011138	7.692308
GOTERM_BP_DIRECT	GO:0006919	3	0.011403	7.692308
GOTERM_BP_DIRECT	GO:0010632	2	0.011784	5.128205
GOTERM_BP_DIRECT	GO:0071347	3	0.013616	7.692308
GOTERM_BP_DIRECT	GO:0007265	3	0.013616	7.692308
GOTERM_BP_DIRECT	GO:0045732	3	0.013616	7.692308
GOTERM_BP_DIRECT	GO:1903721	2	0.013734	5.128205
GOTERM_BP_DIRECT	GO:0071494	2	0.013734	5.128205
GOTERM_BP_DIRECT	GO:0021955	2	0.013734	5.128205
GOTERM_BP_DIRECT	GO:0060440	2	0.013734	5.128205
GOTERM_BP_DIRECT	GO:0060355	2	0.013734	5.128205
GOTERM_BP_DIRECT	GO:0071455	2	0.013734	5.128205
GOTERM_BP_DIRECT	GO:0010742	2	0.013734	5.128205
GOTERM_BP_DIRECT	GO:0031647	3	0.014491	7.692308
GOTERM_BP_DIRECT	GO:0007172	2	0.015682	5.128205
GOTERM_BP_DIRECT	GO:0071312	2	0.015682	5.128205
GOTERM_BP_DIRECT	GO:0030512	3	0.015693	7.692308
GOTERM_BP_DIRECT	GO:0071346	3	0.01631	7.692308
GOTERM_BP_DIRECT	GO:0046825	2	0.017625	5.128205
GOTERM_BP_DIRECT	GO:0071316	2	0.017625	5.128205
GOTERM_BP_DIRECT	GO:0050999	2	0.017625	5.128205
GOTERM_BP_DIRECT	GO:0032886	2	0.017625	5.128205
GOTERM_BP_DIRECT	GO:0070431	2	0.017625	5.128205
GOTERM_BP_DIRECT	GO:1902966	2	0.017625	5.128205
GOTERM_BP_DIRECT	GO:1903140	2	0.017625	5.128205
GOTERM_BP_DIRECT	GO:0032760	3	0.019213	7.692308
GOTERM_BP_DIRECT	GO:0045899	2	0.019564	5.128205
GOTERM_BP_DIRECT	GO:0043068	2	0.019564	5.128205
GOTERM_BP_DIRECT	GO:0033598	2	0.019564	5.128205
GOTERM_BP_DIRECT	GO:0044346	2	0.019564	5.128205
GOTERM_BP_DIRECT	GO:1901522	2	0.019564	5.128205
GOTERM_BP_DIRECT	GO:0045792	2	0.019564	5.128205

GOTERM_BP_DIRECT	GO:0032872	2	0.019564	5.128205
GOTERM_BP_DIRECT	GO:0010907	2	0.019564	5.128205
GOTERM_BP_DIRECT	GO:0007229	3	0.019886	7.692308
GOTERM_BP_DIRECT	GO:0009615	3	0.020225	7.692308
GOTERM_BP_DIRECT	GO:0071803	2	0.0215	5.128205
GOTERM_BP_DIRECT	GO:0006983	2	0.0215	5.128205
GOTERM_BP_DIRECT	GO:1903715	2	0.0215	5.128205
GOTERM_BP_DIRECT	GO:0097011	2	0.0215	5.128205
GOTERM_BP_DIRECT	GO:0071223	2	0.0215	5.128205
GOTERM_BP_DIRECT	GO:0031642	2	0.0215	5.128205
GOTERM_BP_DIRECT	GO:0007166	4	0.022061	10.25641
GOTERM_BP_DIRECT	GO:0006979	3	0.023394	7.692308
GOTERM_BP_DIRECT	GO:1990314	2	0.023432	5.128205
GOTERM_BP_DIRECT	GO:0070417	2	0.023432	5.128205
GOTERM_BP_DIRECT	GO:0060333	2	0.023432	5.128205
GOTERM_BP_DIRECT	GO:0006935	3	0.024493	7.692308
GOTERM_BP_DIRECT	GO:0048546	2	0.025361	5.128205
GOTERM_BP_DIRECT	GO:0033628	2	0.025361	5.128205
GOTERM_BP_DIRECT	GO:0060411	2	0.025361	5.128205
GOTERM_BP_DIRECT	GO:0060396	2	0.025361	5.128205
GOTERM_BP_DIRECT	GO:0032026	2	0.025361	5.128205
GOTERM_BP_DIRECT	GO:0010918	2	0.025361	5.128205
GOTERM_BP_DIRECT	GO:0061029	2	0.025361	5.128205
GOTERM_BP_DIRECT	GO:0031571	2	0.025361	5.128205
GOTERM_BP_DIRECT	GO:0010745	2	0.025361	5.128205
GOTERM_BP_DIRECT	GO:0030308	3	0.02599	7.692308
GOTERM_BP_DIRECT	GO:0050830	3	0.026753	7.692308
GOTERM_BP_DIRECT	GO:0006978	2	0.027285	5.128205
GOTERM_BP_DIRECT	GO:0010659	2	0.027285	5.128205
GOTERM_BP_DIRECT	GO:0070102	2	0.027285	5.128205
GOTERM_BP_DIRECT	GO:0045667	2	0.027285	5.128205
GOTERM_BP_DIRECT	GO:0007260	2	0.027285	5.128205
GOTERM_BP_DIRECT	GO:0051149	2	0.029206	5.128205
GOTERM_BP_DIRECT	GO:0002687	2	0.029206	5.128205
GOTERM_BP_DIRECT	GO:0032270	2	0.029206	5.128205
GOTERM_BP_DIRECT	GO:0046685	2	0.029206	5.128205
GOTERM_BP_DIRECT	GO:0045087	5	0.029358	12.82051
GOTERM_BP_DIRECT	GO:0090399	2	0.031123	5.128205
GOTERM_BP_DIRECT	GO:0019395	2	0.031123	5.128205
GOTERM_BP_DIRECT	GO:0030278	2	0.031123	5.128205
GOTERM_BP_DIRECT	GO:0032966	2	0.031123	5.128205
GOTERM_BP_DIRECT	GO:0010763	2	0.031123	5.128205
GOTERM_BP_DIRECT	GO:0050862	2	0.031123	5.128205
GOTERM_BP_DIRECT	GO:0008637	2	0.033037	5.128205
GOTERM_BP_DIRECT	GO:0050995	2	0.033037	5.128205
GOTERM_BP_DIRECT	GO:0030857	2	0.033037	5.128205

GOTERM_BP_DIRECT	GO:0051403	2	0.033037	5.128205
GOTERM_BP_DIRECT	GO:0070848	2	0.033037	5.128205
GOTERM_BP_DIRECT	GO:0060065	2	0.033037	5.128205
GOTERM_BP_DIRECT	GO:0046325	2	0.033037	5.128205
GOTERM_BP_DIRECT	GO:0048009	2	0.033037	5.128205
GOTERM_BP_DIRECT	GO:0071380	2	0.033037	5.128205
GOTERM_BP_DIRECT	GO:0090090	3	0.033172	7.692308
GOTERM_BP_DIRECT	GO:0030949	2	0.034947	5.128205
GOTERM_BP_DIRECT	GO:0035234	2	0.034947	5.128205
GOTERM_BP_DIRECT	GO:0051044	2	0.034947	5.128205
GOTERM_BP_DIRECT	GO:0051895	2	0.034947	5.128205
GOTERM_BP_DIRECT	GO:1901222	2	0.034947	5.128205
GOTERM_BP_DIRECT	GO:0010759	2	0.036853	5.128205
GOTERM_BP_DIRECT	GO:0031668	2	0.036853	5.128205
GOTERM_BP_DIRECT	GO:0010243	2	0.036853	5.128205
GOTERM_BP_DIRECT	GO:0030220	2	0.036853	5.128205
GOTERM_BP_DIRECT	GO:0001892	2	0.038756	5.128205
GOTERM_BP_DIRECT	GO:0007171	2	0.038756	5.128205
GOTERM_BP_DIRECT	GO:0051899	2	0.038756	5.128205
GOTERM_BP_DIRECT	GO:0030889	2	0.038756	5.128205
GOTERM_BP_DIRECT	GO:0060716	2	0.038756	5.128205
GOTERM_BP_DIRECT	GO:2000010	2	0.038756	5.128205
GOTERM_BP_DIRECT	GO:0010666	2	0.038756	5.128205
GOTERM_BP_DIRECT	GO:0002042	2	0.040655	5.128205
GOTERM_BP_DIRECT	GO:0038096	2	0.040655	5.128205
GOTERM_BP_DIRECT	GO:0033198	2	0.040655	5.128205
GOTERM_BP_DIRECT	GO:1902894	2	0.040655	5.128205
GOTERM_BP_DIRECT	GO:0060324	2	0.040655	5.128205
GOTERM_BP_DIRECT	GO:0070498	2	0.040655	5.128205
GOTERM_BP_DIRECT	GO:0045766	3	0.041493	7.692308
GOTERM_BP_DIRECT	GO:0006809	2	0.04255	5.128205
GOTERM_BP_DIRECT	GO:0090201	2	0.04255	5.128205
GOTERM_BP_DIRECT	GO:0043254	2	0.04255	5.128205
GOTERM_BP_DIRECT	GO:0031929	2	0.04255	5.128205
GOTERM_BP_DIRECT	GO:0045780	2	0.04255	5.128205
GOTERM_BP_DIRECT	GO:0006355	6	0.042975	15.38462
GOTERM_BP_DIRECT	GO:0002053	2	0.044442	5.128205
GOTERM_BP_DIRECT	GO:2000378	2	0.046329	5.128205
GOTERM_BP_DIRECT	GO:0046628	2	0.046329	5.128205
GOTERM_BP_DIRECT	GO:0038061	2	0.046329	5.128205
GOTERM_BP_DIRECT	GO:0014911	2	0.046329	5.128205
GOTERM_BP_DIRECT	GO:0045672	2	0.046329	5.128205
GOTERM_BP_DIRECT	GO:0045821	2	0.046329	5.128205
GOTERM_BP_DIRECT	GO:0045861	2	0.046329	5.128205
GOTERM_BP_DIRECT	GO:0051276	2	0.046329	5.128205
GOTERM_BP_DIRECT	GO:0031954	2	0.046329	5.128205

GOTERM_BP_DIRECT	GO:0097194	2	0.048214	5.128205
GOTERM_BP_DIRECT	GO:0035902	2	0.048214	5.128205
GOTERM_CC_DIRECT	GO:0032991	16	6.98E-13	41.02564
GOTERM_CC_DIRECT	GO:0005829	30	2.63E-10	76.92308
GOTERM_CC_DIRECT	GO:0005634	29	1.49E-08	74.35897
GOTERM_CC_DIRECT	GO:0005654	24	2.44E-08	61.53846
GOTERM_CC_DIRECT	GO:0005737	26	7.66E-07	66.66667
GOTERM_CC_DIRECT	GO:0005739	14	9.80E-07	35.89744
GOTERM_CC_DIRECT	GO:0048471	10	8.16E-06	25.64103
GOTERM_CC_DIRECT	GO:0005667	6	6.49E-05	15.38462
GOTERM_CC_DIRECT	GO:0090575	5	7.34E-05	12.82051
GOTERM_CC_DIRECT	GO:0045121	6	9.60E-05	15.38462
GOTERM_CC_DIRECT	GO:0017053	4	2.17E-04	10.25641
GOTERM_CC_DIRECT	GO:0031234	4	2.72E-04	10.25641
GOTERM_CC_DIRECT	GO:0000785	9	6.02E-04	23.07692
GOTERM_CC_DIRECT	GO:0005886	20	6.75E-04	51.28205
GOTERM_CC_DIRECT	GO:0005925	6	0.001064	15.38462
GOTERM_CC_DIRECT	GO:0005815	4	0.003234	10.25641
GOTERM_CC_DIRECT	GO:0005576	11	0.004439	28.20513
GOTERM_CC_DIRECT	GO:0016324	5	0.004956	12.82051
GOTERM_CC_DIRECT	GO:0000791	3	0.006101	7.692308
GOTERM_CC_DIRECT	GO:0031093	3	0.006875	7.692308
GOTERM_CC_DIRECT	GO:0005901	3	0.008993	7.692308
GOTERM_CC_DIRECT	GO:0035976	2	0.009247	5.128205
GOTERM_CC_DIRECT	GO:0016323	4	0.009699	10.25641
GOTERM_CC_DIRECT	GO:0045202	5	0.012408	12.82051
GOTERM_CC_DIRECT	GO:0044294	2	0.018412	5.128205
GOTERM_CC_DIRECT	GO:1904813	3	0.022195	7.692308
GOTERM_CC_DIRECT	GO:0016604	4	0.024763	10.25641
GOTERM_CC_DIRECT	GO:0030424	4	0.029102	10.25641
GOTERM_CC_DIRECT	GO:0005770	3	0.030793	7.692308
GOTERM_CC_DIRECT	GO:0098978	4	0.03163	10.25641
GOTERM_CC_DIRECT	GO:0044214	2	0.032903	5.128205
GOTERM_CC_DIRECT	GO:0043197	3	0.038806	7.692308
GOTERM_CC_DIRECT	GO:0043025	4	0.0394	10.25641
GOTERM_CC_DIRECT	GO:0016020	10	0.040032	25.64103
GOTERM_MF_DIRECT	GO:0019899	17	1.23E-17	43.58974
GOTERM_MF_DIRECT	GO:0019901	16	2.24E-14	41.02564
GOTERM_MF_DIRECT	GO:0019903	10	1.72E-13	25.64103
GOTERM_MF_DIRECT	GO:0042802	21	9.21E-12	53.84615
GOTERM_MF_DIRECT	GO:0031625	12	1.39E-11	30.76923
GOTERM_MF_DIRECT	GO:0008134	10	2.68E-10	25.64103
GOTERM_MF_DIRECT	GO:0004672	10	4.84E-08	25.64103
GOTERM_MF_DIRECT	GO:0005515	39	1.99E-07	100
GOTERM_MF_DIRECT	GO:0030235	4	6.34E-07	10.25641
GOTERM_MF_DIRECT	GO:0004713	6	3.07E-06	15.38462

GOTERM_MF_DIRECT	GO:0004714	6	3.49E-06	15.38462
GOTERM_MF_DIRECT	GO:0042826	6	6.57E-06	15.38462
GOTERM_MF_DIRECT	GO:0003700	9	1.21E-05	23.07692
GOTERM_MF_DIRECT	GO:0044877	8	1.66E-05	20.51282
GOTERM_MF_DIRECT	GO:0005524	13	3.23E-05	33.33333
GOTERM_MF_DIRECT	GO:0061629	6	3.63E-05	15.38462
GOTERM_MF_DIRECT	GO:0051117	5	3.63E-05	12.82051
GOTERM_MF_DIRECT	GO:0001221	4	6.11E-05	10.25641
GOTERM_MF_DIRECT	GO:0016301	6	8.70E-05	15.38462
GOTERM_MF_DIRECT	GO:0003690	5	8.91E-05	12.82051
GOTERM_MF_DIRECT	GO:0001223	4	9.51E-05	10.25641
GOTERM_MF_DIRECT	GO:0000976	6	1.11E-04	15.38462
GOTERM_MF_DIRECT	GO:0001784	4	1.16E-04	10.25641
GOTERM_MF_DIRECT	GO:0004674	7	1.24E-04	17.94872
GOTERM_MF_DIRECT	GO:0003677	11	1.74E-04	28.20513
GOTERM_MF_DIRECT	GO:0003682	7	3.19E-04	17.94872
GOTERM_MF_DIRECT	GO:0001228	7	3.41E-04	17.94872
GOTERM_MF_DIRECT	GO:0002039	4	3.95E-04	10.25641
GOTERM_MF_DIRECT	GO:0004707	3	4.68E-04	7.692308
GOTERM_MF_DIRECT	GO:0005125	5	5.92E-04	12.82051
GOTERM_MF_DIRECT	GO:0004708	3	5.96E-04	7.692308
GOTERM_MF_DIRECT	GO:0000979	3	7.38E-04	7.692308
GOTERM_MF_DIRECT	GO:0008022	5	7.99E-04	12.82051
GOTERM_MF_DIRECT	GO:0046982	6	8.54E-04	15.38462
GOTERM_MF_DIRECT	GO:0005158	3	0.001066	7.692308
GOTERM_MF_DIRECT	GO:0070412	3	0.001066	7.692308
GOTERM_MF_DIRECT	GO:0043274	3	0.001158	7.692308
GOTERM_MF_DIRECT	GO:0002020	4	0.001377	10.25641
GOTERM_MF_DIRECT	GO:0004712	3	0.001559	7.692308
GOTERM_MF_DIRECT	GO:0047485	4	0.001647	10.25641
GOTERM_MF_DIRECT	GO:0019904	5	0.001897	12.82051
GOTERM_MF_DIRECT	GO:0051059	3	0.002017	7.692308
GOTERM_MF_DIRECT	GO:0051721	3	0.002267	7.692308
GOTERM_MF_DIRECT	GO:0000978	9	0.002437	23.07692
GOTERM_MF_DIRECT	GO:0097718	3	0.002531	7.692308
GOTERM_MF_DIRECT	GO:0042169	3	0.0031	7.692308
GOTERM_MF_DIRECT	GO:0000981	9	0.00349	23.07692
GOTERM_MF_DIRECT	GO:0030331	3	0.003724	7.692308
GOTERM_MF_DIRECT	GO:0004715	3	0.003889	7.692308
GOTERM_MF_DIRECT	GO:0005178	4	0.004168	10.25641
GOTERM_MF_DIRECT	GO:0001227	5	0.004337	12.82051
GOTERM_MF_DIRECT	GO:0070888	3	0.004402	7.692308
GOTERM_MF_DIRECT	GO:0008083	4	0.004539	10.25641
GOTERM_MF_DIRECT	GO:1990782	3	0.004579	7.692308
GOTERM_MF_DIRECT	GO:0004879	3	0.005322	7.692308
GOTERM_MF_DIRECT	GO:0035033	2	0.006048	5.128205

GOTERM_MF_DIRECT	GO:0097110	3	0.008561	7.692308
GOTERM_MF_DIRECT	GO:0043125	2	0.010061	5.128205
GOTERM_MF_DIRECT	GO:0042803	6	0.013752	15.38462
GOTERM_MF_DIRECT	GO:0004725	3	0.019724	7.692308
GOTERM_MF_DIRECT	GO:0016004	2	0.022004	5.128205
GOTERM_MF_DIRECT	GO:0004861	2	0.023981	5.128205
GOTERM_MF_DIRECT	GO:0043560	2	0.023981	5.128205
GOTERM_MF_DIRECT	GO:0045296	4	0.025685	10.25641
GOTERM_MF_DIRECT	GO:0043565	4	0.028265	10.25641
GOTERM_MF_DIRECT	GO:0005161	2	0.029888	5.128205
GOTERM_MF_DIRECT	GO:0030297	2	0.029888	5.128205
GOTERM_MF_DIRECT	GO:0001216	2	0.031849	5.128205
GOTERM_MF_DIRECT	GO:0044325	3	0.032465	7.692308
GOTERM_MF_DIRECT	GO:0001046	2	0.03576	5.128205
GOTERM_MF_DIRECT	GO:0005102	4	0.045397	10.25641
GOTERM_MF_DIRECT	GO:0043548	2	0.04547	5.128205
GOTERM_MF_DIRECT	GO:0044389	2	0.047401	5.128205
GOTERM_MF_DIRECT	GO:0035035	2	0.049328	5.128205
