# Supplementary material for: Integrating Network Pharmacology and Experimental Validation to Elucidate the Mechanism of Yiqi Yangyin Decoction in Suppressing Non-Small-Cell Lung Cancer
Source: Biomed Res Int. 2023 Feb 20;2023:4967544. doi: 10.1155/2023/4967544 (PMC9980286; doi:10.1155/2023/4967544)
Supplement: Supplementary 7 — Supplementary Table 6: the detailed pathway information of YYD against NSCLC. [file 4967544.f7.pdf]

Supplementary Table 6 The detailed pathway information of YYD against NSCLC

Category	ID	Count	%	PValue
KEGG_PATHWAY	hsa05200	31	79.48718	3.45E-29
KEGG_PATHWAY	hsa05205	22	56.41026	1.76E-24
KEGG_PATHWAY	hsa01522	18	46.15385	1.23E-23
KEGG_PATHWAY	hsa05163	22	56.41026	1.32E-23
KEGG_PATHWAY	hsa05167	21	53.84615	2.84E-23
KEGG_PATHWAY	hsa05135	18	46.15385	5.13E-21
KEGG_PATHWAY	hsa05417	20	51.28205	9.97E-21
KEGG_PATHWAY	hsa05235	16	41.02564	1.35E-20
KEGG_PATHWAY	hsa05166	20	51.28205	1.85E-20
KEGG_PATHWAY	hsa05215	16	41.02564	5.34E-20
KEGG_PATHWAY	hsa05212	15	38.46154	8.46E-20
KEGG_PATHWAY	hsa05161	18	46.15385	9.80E-20
KEGG_PATHWAY	hsa04625	16	41.02564	1.61E-19
KEGG_PATHWAY	hsa05210	15	38.46154	5.42E-19
KEGG_PATHWAY	hsa04151	21	53.84615	5.26E-18
KEGG_PATHWAY	hsa05213	13	33.33333	1.16E-17
KEGG_PATHWAY	hsa05219	12	30.76923	1.30E-17
KEGG_PATHWAY	hsa05162	16	41.02564	1.49E-17
KEGG_PATHWAY	hsa05208	18	46.15385	2.44E-17
KEGG_PATHWAY	hsa04012	14	35.89744	2.85E-17
KEGG_PATHWAY	hsa05224	16	41.02564	3.53E-17
KEGG_PATHWAY	hsa05160	16	41.02564	9.67E-17
KEGG_PATHWAY	hsa04917	13	33.33333	1.33E-16
KEGG_PATHWAY	hsa04380	15	38.46154	1.83E-16
KEGG_PATHWAY	hsa04933	14	35.89744	2.62E-16
KEGG_PATHWAY	hsa05220	13	33.33333	3.81E-16
KEGG_PATHWAY	hsa04620	14	35.89744	4.45E-16
KEGG_PATHWAY	hsa04660	14	35.89744	4.45E-16
KEGG_PATHWAY	hsa05418	15	38.46154	5.97E-16
KEGG_PATHWAY	hsa01521	13	33.33333	6.23E-16
KEGG_PATHWAY	hsa04659	14	35.89744	7.40E-16
KEGG_PATHWAY	hsa05165	19	48.71795	8.69E-16
KEGG_PATHWAY	hsa04668	14	35.89744	1.21E-15
KEGG_PATHWAY	hsa04010	18	46.15385	2.69E-15
KEGG_PATHWAY	hsa04919	14	35.89744	3.50E-15
KEGG_PATHWAY	hsa05131	17	43.58974	3.76E-15
KEGG_PATHWAY	hsa04510	16	41.02564	4.16E-15
KEGG_PATHWAY	hsa05169	16	41.02564	4.47E-15
KEGG_PATHWAY	hsa04657	13	33.33333	5.65E-15
KEGG_PATHWAY	hsa04926	14	35.89744	8.14E-15
KEGG_PATHWAY	hsa05207	16	41.02564	9.30E-15
KEGG_PATHWAY	hsa05142	13	33.33333	1.56E-14
KEGG_PATHWAY	hsa05222	12	30.76923	1.91E-13

KEGG_PATHWAY	hsa04068	13	33.33333	3.36E-13
KEGG_PATHWAY	hsa04210	13	33.33333	5.30E-13
KEGG_PATHWAY	hsa05218	11	28.20513	5.85E-13
KEGG_PATHWAY	hsa05214	11	28.20513	8.95E-13
KEGG_PATHWAY	hsa04066	12	30.76923	1.29E-12
KEGG_PATHWAY	hsa05132	15	38.46154	2.14E-12
KEGG_PATHWAY	hsa04932	13	33.33333	2.56E-12
KEGG_PATHWAY	hsa04218	13	33.33333	2.76E-12
KEGG_PATHWAY	hsa04722	12	30.76923	3.45E-12
KEGG_PATHWAY	hsa05170	14	35.89744	5.23E-12
KEGG_PATHWAY	hsa05171	14	35.89744	1.65E-11
KEGG_PATHWAY	hsa05230	10	25.64103	1.99E-11
KEGG_PATHWAY	hsa05223	10	25.64103	2.59E-11
KEGG_PATHWAY	hsa05226	12	30.76923	4.11E-11
KEGG_PATHWAY	hsa05133	10	25.64103	4.27E-11
KEGG_PATHWAY	hsa05130	13	33.33333	4.41E-11
KEGG_PATHWAY	hsa04935	11	28.20513	1.00E-10
KEGG_PATHWAY	hsa04630	12	30.76923	1.02E-10
KEGG_PATHWAY	hsa05225	12	30.76923	1.52E-10
KEGG_PATHWAY	hsa04658	10	25.64103	2.47E-10
KEGG_PATHWAY	hsa04621	12	30.76923	4.05E-10
KEGG_PATHWAY	hsa04915	11	28.20513	4.38E-10
KEGG_PATHWAY	hsa05231	10	25.64103	4.40E-10
KEGG_PATHWAY	hsa04936	11	28.20513	5.81E-10
KEGG_PATHWAY	hsa05211	9	23.07692	6.91E-10
KEGG_PATHWAY	hsa05120	9	23.07692	7.78E-10
KEGG_PATHWAY	hsa04931	10	25.64103	1.06E-09
KEGG_PATHWAY	hsa01524	9	23.07692	1.10E-09
KEGG_PATHWAY	hsa05203	12	30.76923	1.22E-09
KEGG_PATHWAY	hsa05145	10	25.64103	1.47E-09
KEGG_PATHWAY	hsa05140	9	23.07692	1.69E-09
KEGG_PATHWAY	hsa04071	10	25.64103	2.52E-09
KEGG_PATHWAY	hsa04662	9	23.07692	2.82E-09
KEGG_PATHWAY	hsa05164	11	28.20513	3.59E-09
KEGG_PATHWAY	hsa04014	12	30.76923	5.45E-09
KEGG_PATHWAY	hsa05152	11	28.20513	5.91E-09
KEGG_PATHWAY	hsa04370	8	20.51282	7.62E-09
KEGG_PATHWAY	hsa05206	13	33.33333	8.20E-09
KEGG_PATHWAY	hsa04062	11	28.20513	1.10E-08
KEGG_PATHWAY	hsa05216	7	17.94872	1.42E-08
KEGG_PATHWAY	hsa04912	8	20.51282	1.89E-07
KEGG_PATHWAY	hsa04140	9	23.07692	2.06E-07
KEGG_PATHWAY	hsa05020	11	28.20513	3.09E-07
KEGG_PATHWAY	hsa04914	8	20.51282	3.57E-07
KEGG_PATHWAY	hsa05146	8	20.51282	3.57E-07
KEGG_PATHWAY	hsa04921	9	23.07692	4.07E-07

KEGG_PATHWAY	hsa05321	7	17.94872	4.58E-07
KEGG_PATHWAY	hsa05221	7	17.94872	5.50E-07
KEGG_PATHWAY	hsa04024	10	25.64103	5.51E-07
KEGG_PATHWAY	hsa04664	7	17.94872	6.01E-07
KEGG_PATHWAY	hsa04137	7	17.94872	8.46E-07
KEGG_PATHWAY	hsa04115	7	17.94872	9.19E-07
KEGG_PATHWAY	hsa05168	13	33.33333	1.39E-06
KEGG_PATHWAY	hsa05202	9	23.07692	2.26E-06
KEGG_PATHWAY	hsa04015	9	23.07692	4.25E-06
KEGG_PATHWAY	hsa05134	6	15.38462	5.87E-06
KEGG_PATHWAY	hsa05010	11	28.20513	6.88E-06
KEGG_PATHWAY	hsa04920	6	15.38462	1.51E-05
KEGG_PATHWAY	hsa04650	7	17.94872	2.22E-05
KEGG_PATHWAY	hsa04910	7	17.94872	3.57E-05
KEGG_PATHWAY	hsa05022	11	28.20513	4.48E-05
KEGG_PATHWAY	hsa04550	7	17.94872	4.55E-05
KEGG_PATHWAY	hsa04540	6	15.38462	4.94E-05
KEGG_PATHWAY	hsa04211	6	15.38462	5.22E-05
KEGG_PATHWAY	hsa04072	7	17.94872	5.52E-05
KEGG_PATHWAY	hsa05323	6	15.38462	6.45E-05
KEGG_PATHWAY	hsa04934	7	17.94872	7.14E-05
KEGG_PATHWAY	hsa04150	7	17.94872	7.40E-05
KEGG_PATHWAY	hsa04750	6	15.38462	8.29E-05
KEGG_PATHWAY	hsa04064	6	15.38462	1.10E-04
KEGG_PATHWAY	hsa04310	7	17.94872	1.19E-04
KEGG_PATHWAY	hsa04928	6	15.38462	1.20E-04
KEGG_PATHWAY	hsa04725	6	15.38462	1.63E-04
KEGG_PATHWAY	hsa04613	7	17.94872	2.19E-04
KEGG_PATHWAY	hsa04611	6	15.38462	2.52E-04
KEGG_PATHWAY	hsa04110	6	15.38462	2.71E-04
KEGG_PATHWAY	hsa04622	5	12.82051	2.95E-04
KEGG_PATHWAY	hsa05415	7	17.94872	3.13E-04
KEGG_PATHWAY	hsa04728	6	15.38462	3.36E-04
KEGG_PATHWAY	hsa01523	4	10.25641	3.47E-04
KEGG_PATHWAY	hsa04810	7	17.94872	4.59E-04
KEGG_PATHWAY	hsa05332	4	10.25641	9.45E-04
KEGG_PATHWAY	hsa04530	6	15.38462	0.0010388
KEGG_PATHWAY	hsa04930	4	10.25641	0.0012337
KEGG_PATHWAY	hsa04360	6	15.38462	0.0014463
KEGG_PATHWAY	hsa04623	4	10.25641	0.0030563
KEGG_PATHWAY	hsa04929	4	10.25641	0.0031964
KEGG_PATHWAY	hsa04371	5	12.82051	0.0038049
KEGG_PATHWAY	hsa04520	4	10.25641	0.0042877
KEGG_PATHWAY	hsa05100	4	10.25641	0.0053834
KEGG_PATHWAY	hsa04217	5	12.82051	0.0061297
KEGG_PATHWAY	hsa04215	3	7.692308	0.009601

KEGG_PATHWAY	hsa04666	4	10.25641	0.0101799
KEGG_PATHWAY	hsa04916	4	10.25641	0.0113606
KEGG_PATHWAY	hsa05143	3	7.692308	0.0127048
KEGG_PATHWAY	hsa04670	4	10.25641	0.0157285
KEGG_PATHWAY	hsa04726	4	10.25641	0.0160985
KEGG_PATHWAY	hsa04940	3	7.692308	0.0169271
KEGG_PATHWAY	hsa04152	4	10.25641	0.0180226
KEGG_PATHWAY	hsa05030	3	7.692308	0.0216628
KEGG_PATHWAY	hsa05144	3	7.692308	0.0224999
KEGG_PATHWAY	hsa04114	4	10.25641	0.0226911
KEGG_PATHWAY	hsa04020	5	12.82051	0.0246576
KEGG_PATHWAY	hsa04340	3	7.692308	0.0277969
KEGG_PATHWAY	hsa04923	3	7.692308	0.0277969
KEGG_PATHWAY	hsa04723	4	10.25641	0.0310891
KEGG_PATHWAY	hsa05416	3	7.692308	0.0315798
KEGG_PATHWAY	hsa04261	4	10.25641	0.0321713
KEGG_PATHWAY	hsa04213	3	7.692308	0.0335437
KEGG_PATHWAY	hsa05012	5	12.82051	0.034238
KEGG_PATHWAY	hsa05217	3	7.692308	0.0345433
KEGG_PATHWAY	hsa04720	3	7.692308	0.0386564
KEGG_PATHWAY	hsa05031	3	7.692308	0.0407802
