# Supplementary material for: Integrating Network Pharmacology and Experimental Validation to Elucidate the Mechanism of Yiqi Yangyin Decoction in Suppressing Non-Small-Cell Lung Cancer
Source: Biomed Res Int. 2023 Feb 20;2023:4967544. doi: 10.1155/2023/4967544 (PMC9980286; doi:10.1155/2023/4967544)
Supplement: Supplementary 8 — Supplementary Table 7: cluster analysis for PPI network. [file 4967544.f8.pdf]

Supplementary Table 7 Cluster analysis for PPI network

Cluster	Score	Nodes	Edges	Genes
1	14	50	343	HSP90AA1, CDK4, AKT1, PTK2, AURKA, IL2, CCND1, MMP3, IL1B, ERBB3, CXCL10, E2F2, FOS, CXCL8, ESR2, E2F1, MTOR, PTGS2, JUN, RUNX2, PTEN, CDKN1A, CDKN2A, MYC, MPO, CCL2, IFNG, IL4, ICAM1, PTPRC, CAV1, PCNA, ALB, EGFR, CDK5, CRP, IL6, CDK6, TNF, IGF1R, MAPK14, CHEK1, TYMS, ERBB2, TOP2A, AURKB, BIRC5, PLK1, MDM2, IL1A
2	7.935	32	123	ESR1, IKBKB, HIF1A, MET, MAPK1, NFKBIA, MMP1, CCNB1, IL10, PLG, TP53, CCNA2, BAX, PGR, ANXA5, HSPA8, CYCS, TERT, GSK3B, CTNNA1, CASP9, CDK1, EGF, CASP8, SRC, ERBB4, PTPN1, CDK2, MMP9, RB1, BCL2, STAT1
3	5.923	27	77	PIK3R1, VEGFA, MAPK8, BCL2L1, CASP3, HSPA5, NOS2, MAPK10, PPARG, LCK, PTPN11, HDAC2, APP, PDPK1, ADAM17, RAC1, RASA1, PRKCB, MCL1, CHUK, CD40LG, KDR, NR3C1, CASP1, NFE2L2, AR, RELA
4	5	5	10	CYP3A4, CYP1A2, CYP2C9, GSTM1, GSTP1
5	4	4	6	PTGS1, PLA2G4A, PLA2G2A, ALOX5
6	4	5	8	CYP17A1, CYP1A1, CYP19A1, HSD17B1, CYP1B1
7	3.5	5	7	PRKCA, MAP2K1, MAP2K4, BRAF, RAF1
